# Genome-wide association studies for 30 haematological and blood clinical-biochemical traits in Large White pigs reveal genomic regions affecting intermediate phenotypes

**DOI:** 10.1038/s41598-019-43297-1

**Published:** 2019-05-07

**Authors:** Samuele Bovo, Gianluca Mazzoni, Francesca Bertolini, Giuseppina Schiavo, Giuliano Galimberti, Maurizio Gallo, Stefania Dall’Olio, Luca Fontanesi

**Affiliations:** 1 Department of Agricultural and Food Sciences, Division of Animal Sciences, University of Bologna, Viale G. Fanin 46, 40127 Bologna, Italy; 2 Department of Health Technology, Technical University of Denmark (DTU), Lyngby, 2800 Denmark; 3 National Institute of Aquatic Resources, Technical University of Denmark (DTU), Lyngby, 2800 Denmark; 4 Department of Statistical Sciences “Paolo Fortunati”, University of Bologna, Via delle Belle Arti 41, 40126 Bologna, Italy; 5 Associazione Nazionale Allevatori Suini (ANAS), Via Nizza 53, 00198 Roma, Italy

**Keywords:** Data integration, Agricultural genetics, Functional genomics, Quantitative trait, Homeostasis

## Abstract

Haematological and clinical-biochemical parameters are considered indicators of the physiological/health status of animals and might serve as intermediate phenotypes to link physiological aspects to production and disease resistance traits. The dissection of the genetic variability affecting these phenotypes might be useful to describe the resilience of the animals and to support the usefulness of the pig as animal model. Here, we analysed 15 haematological and 15 clinical-biochemical traits in 843 Italian Large White pigs, via three genome-wide association scan approaches (single-trait, multi-trait and Bayesian). We identified 52 quantitative trait loci (QTLs) associated with 29 out of 30 analysed blood parameters, with the most significant QTL identified on porcine chromosome 14 for basophil count. Some QTL regions harbour genes that may be the obvious candidates: QTLs for cholesterol parameters identified genes (*ADCY8*, *APOB*, *ATG5*, *CDKAL1*, *PCSK5*, *PRL* and *SOX6*) that are directly involved in cholesterol metabolism; other QTLs highlighted genes encoding the enzymes being measured [ALT (known also as GPT) and AST (known also as GOT)]. Moreover, the multivariate approach strengthened the association results for several candidate genes. The obtained results can contribute to define new measurable phenotypes that could be applied in breeding programs as proxies for more complex traits.

## Introduction

With the advent of high throughput single nucleotide polymorphism (SNP) genotyping tools in different livestock species, genome-wide association studies have become common approaches to identify markers associated with many different phenotypic traits, spanning from monogenic to more complex quantitative traits. In pigs, a large number of studies has been focused on production traits that are usually directly targeted in breeding programs (e.g.^1–4^). Other studies have addressed basic physiological, biochemical and molecular parameters or other intermediate phenotypes (also known as internal phenotypes) that might be indirectly related to economic traits^5–7^. These traits are closer to basic functions and for this reason they might be useful to dissect complex production traits by capturing the fine mechanisms underlying their biological control. The use of intermediate phenotypes could be also useful to better define the pig as animal model to explain basic biological mechanisms and associated diseases^8^.

Haematological and blood clinical-biochemical parameters reflect the physiological and health status of the animals and are used as biomarkers to describe pathological or sub-pathological conditions^9^. They are considered indicators of immune functions and components of the adaptive immune system in both humans and livestock (e.g.^10^). They are also directly linked to biochemical pathways, homeostasis and transport of biomolecules (e.g. cholesterol and other metabolite levels) or enzyme functions and activities (e.g. hepatic and muscle enzyme stress indicators). Therefore, the identification of DNA markers associated with these traits in animals might provide information to indirectly overcome the limited genetic progress that traditional livestock selection programs have on disease resistance, robustness and resilience^11^. In addition, variability in the basal levels of these parameters has been also associated with different predisposition to a wide range of human diseases, including cardiovascular, cancer, metabolic, infection and immune disorders (e.g.^12–16^).

Haematological traits can be classified according to the blood cells from which they derive: erythrocyte-related traits, leukocyte-related traits and platelet-related traits. Clinical-biochemical traits include the amount of biochemical and mineral components of the blood and the activities of several enzymes. Most of blood parameters can be regarded as the result of simpler biological processes than classical performance and production traits and could be considered as intermediate (or internal) phenotypes^8^.

Several QTL studies investigating haematological and immune capacity traits have been reported in pigs. Most of these studies used F2 and backcross reference families having, as parental animals, pigs of different breeds and lines, including wild boars, commercial European breeds (Duroc, Landrace, Large White, Pietrain, Yorkshire) and Asian breeds or lines (Erhulian, Korean native pig, Meishan, Minzhu, Songliao Black Pig)^17–26^. A few other authors have applied genome-wide association studies in Chinese Sutai pigs and in the German Landrace population^27,28^. Results of these works reported few overlapping QTL regions, probably derived by the genetic heterogeneity of the investigated pig populations, differences among the analysed traits or parameters and the variety of the experimental designs.

This study reports the results obtained combining different genome-wide association strategies, i.e. single-marker (single-trait and multivariate associations) and Bayesian (windows-based single-trait) association studies, for a total of 30 haematological and blood clinical-biochemical parameters in Italian Large White heavy pigs. Based on different statistics, these methods present different pros and cons, and their combined use could overcome drawbacks derived by the population structure or by the experimental design. The single-maker approach is one of the most adopted genome scan methods. In its simplest version, this approach is implemented by means of a liner model relating, via an additive model, a phenotype **y** (measured in *n* individuals) to genotypes **x** (*aa*/*aA*/*AA* usually coded as 2/1/0, according to the number of copies of the minor allele *a*)^29^. Implemented as a linear mixed model (LMM), the regression model is augmented by (i) a random genomic effect **g** that account for sample relatedness (population stratification) via a relatedness matrix **K** [either a pedigree-based kinship matrix (**A**) or a genome-based matrix (**G**) also known as Genomic Relationship Matrix (GRM)] and (ii) additional fixed effects accounting for other confounding factors (e.g. sex, body weight)^29^. However, this approach fits one SNP at a time, and multiple testing correction of results is an issue that limits the power of this approach^30^. Moreover, due to imperfect linkage disequilibrium (LD), the effect of a putative gene/QTL can be only in part captured by a single SNP. The Bayesian approach, here implemented as multi-marker windows-based approach, can overcome these limits by fitting multiple markers simultaneously. Over the years several models have been proposed (e.g. BayesA, BayesB, BayesC, BayesD, BayesR) differing mainly in the distributional assumption of the SNP effect. In Bayesian analyses inference of marker association is based on the posterior distribution of markers effect, estimated using Markov Chain Monte Carlo (MCMC) methods and considering SNPs effects within a defined genome window^29^. Lastly, by analysing more phenotypes simultaneously, the multivariate method can increase the statistical power and identify pleiotropic loci^31^. In doing so, the multivariate linear mixed model (mvLMM) adds the cross-traits covariance as extra information^31^. Single-marker and Bayesian approaches have been already efficiently used together to detect QTLs affecting haematological and blood clinical-biochemical parameters in pigs (e.g.^28,32–34^).

Results obtained in this study were then compared to what was reported by previous works carried out in different pig populations using the same or similar blood derived phenotypes. Identified QTLs showed a low chromosome region overlap with those mapped in other studies. Some of them highlighted genomic regions harbouring genes that, for their functions, may be the obvious candidates explaining the detected genetic variability.

## Methods

### Animals, blood collection and analyses

All animals used in this study were kept according to the Italian and European legislations for pig production. All procedures described are in compliance with Italian and European Union regulations for animal care and slaughter. Performance testing of the animals was carried out under the national selection program of heavy pigs. Pigs were not raised or treated in any way for the purpose of this study. Animals were slaughtered in an approved commercial abattoir following the regular procedures for commercial pig slaughtering at the end of their production cycle. Pigs were sampled after slaughtering. For all these reasons no other ethical statements are needed.

Genome-wide association studies were conducted on a total of 843 performance tested Italian Large White pigs (278 castrated males and 565 gilts, obtained from 86 boars and 377 litters), slaughtered over 25 different days. These animals were part of the sib-testing program of the Italian Large White pig population that is based on triplets of pigs of the same litter (two females and one castrated male) that are individually performance tested at the Central Station of the National Pig Breeder Association (ANAS) for the genetic evaluation of a boar from the same litter (sib-testing). Pigs started their performance evaluation at 30–45 days of age until they reached 155 ± 5 kg live weight^35^. All animals were fed with the same standard commercial feed for fattening pigs under the production rules of the Parma and San Daniele dry-cured ham consortia. At the end of the test, animals were transported to the same commercial abattoir where they were slaughtered with standard procedures in the morning (07.00–08.00 a.m.; after overnight fasting of about 12 h) using electrical stunning. Blood was collected just after jugulation and exsanguination, into an EDTA containing tube and a serum separator tube with gel separator and clot activator (Vacutest Kima s.r.l.).

A total of 15 haematological parameters (*erythrocyte traits*: red blood cell count, RBC; haemoglobin, HGB; haematocrit, HCT; mean corpuscular volume, MCV; mean corpuscular haemoglobin, MCH; mean corpuscular haemoglobin concentration, MCHC; and red cell distribution width, RDW; *leukocyte traits*: white blood cell count, WBC; lymphocyte count, LYMPHO; neutrophil count, NEUTRO; eosinophil count, EOSI; basophil count, BASO; and monocyte count, MONO; *platelet traits*: platelet count, PLT; and mean platelet volume, MPV) and 15 clinical-biochemical parameters (*lipid related parameters*: Total cholesterol, T-Chol; high-density lipoprotein cholesterol, HDL-Chol; low-density lipoprotein cholesterol, LDL-Chol; triglycerides, TG; non-esterified fatty acids, NEFA; *metabolism and protein related parameters*: glucose, Glu; urea, UA; total bilirubin, T-Bil; total proteins, T-Prot; albumin, Alb; albumin/globulins ratio, Alb/Glob; creatine kinase, CK; *enzyme activities*: Alkaline phosphatase, ALP; Alanine aminotransferase, ALT; Aspartate aminotransferase, AST) were measured on an Olympus AU 400 (Beckman Coulter) automated blood analyser at the Veterinary Haematological Laboratory of the University of Bologna, using standard procedures.

### Genotyping

DNA was extracted from blood using the Wizard Genomic DNA Purification kit (Promega Corporation, Madison, WI, USA). Animals were then genotyped with the Illumina PorcineSNP60 BeadChip v.2 (Illumina Inc., San Diego, CA, USA), which interrogates 61,565 SNPs, using standard procedures. Genotype calls were conducted by using the Genotyping Module in GenomeStudio software 1.0.2.20706 (Illumina Inc.). Genotypes with an Illumina GenCall score (GC; GenCall Version 6.3.0) below 0.15 were assigned as missing. PLINK v.1.07^36^ was used for quality checks. Briefly, samples with a genotype missing rate >0.9 were discarded (proportion of markers that failed on each sample; command* --mind 0*.*1*) while SNPs were discarded if they presented (i) a call rate <0.95 (proportion of samples for which no genotype calls; command* --geno 0*.*05*), (ii) a Hardy-Weinberg equilibrium (HWE) *p*-value < 0.001 (command* --hwe 0*.*001*) and (iii) a minor allele frequency (MAF) <0.05 (command* --maf 0*.*05*). A total of 3,605, 2,457 and 9,555 variants were discarded, respectively. None of the analysed pig was discarded. Out of the 45,536 retained SNPs, a total of 40,064 markers (~88%) were uniquely mapped to the Sscrofa11.1 pig genome version (including unassembled scaffolds) as previously described^35,37^. Markers assigned to sex chromosomes were not used in this study.

### Data analyses

#### Data transformations

The Box-Cox transformation method^38^ was used to normalize each blood parameter as follows:

1 $$BC(x)=\{\begin{array}{c}\begin{array}{cc}\mathrm{Log}(x) & if=0\end{array}\\ \begin{array}{cc}\frac{{x}^{\lambda }-1}{\lambda } & if\ne 0\end{array}\end{array}$$

The optimal value for λ was selected by maximum log likelihood, considering a uniform grid of 3,001 values of λ in an interval between −3 and +3. The effects of sex, weight and slaughtering date have been considered during the normalization process. Data normalization was done in R v. 3.0.2^39^ by using the “MASS” and “CAR” packages.

#### Confounders removal and correlation network

Environmental and technical factors can affect the levels of blood parameters, so it is important to remove these confounding effects. Here, residuals derived from a linear regression model were considered. The basic model was:

2 $${y}_{i}={\beta }_{0}+{\beta }_{w}{w}_{i}+{\beta }_{s}{s}_{i}+\sum _{j=1}^{J-1}{\beta }_{Cj}{d}_{ij}+{\varepsilon }_{i}$$

where *y*_*i*_ is the level of the blood parameter for the *i*^*th*^ animal [after the Box-Cox transformation; see Eq. (1)], *β*_*0*_ is the intercept term, *w*_*i*_ indicates the weight of the *i*^*th*^ animal, *s*_*i*_ is a dummy variable representing the sex of the *i*^*th*^ animal, *d*_*i1*_, *…*, *d*_*i(J*−*1)*_ is a set of *J* = 25 dummy variables coding the blood collection date for the *i*^*th*^ animal, while *β*_*w*_, *β*_*s*_ and *β*_*C*j_ are the corresponding regression coefficients and ε_i_ is the error term.

Confounding effects are removed by computing the residuals:

3 $${e}_{i}={y}_{i}-{\hat{y}}_{i}$$

with

4 $${\hat{y}}_{i}={b}_{0}+{b}_{w}{w}_{i}+{b}_{s}{s}_{i}+\sum _{j=1}^{J-1}{b}_{Cj}{d}_{ij}$$

where *b*_*0*_, *b*_*w*_, *b*_*s*_, *b*_*Cj*_ (*j* = *1*, *…*, *J* − *1*) are the least squares estimates of model parameters.

Dependences among measured blood parameters were investigated with a correlation network. We obtained a Pearson’s correlation matrix **R**, whose generic entry *r* (i.e. the Pearson’s correlation coefficient between the *h*^*th*^ and the *k*^*th*^ residual blood parameter level) is:

5 $${r}_{hk}=\frac{{\sum }_{i=1}^{n}{e}_{ih}{e}_{ik}}{\sqrt{({\sum }_{i=1}^{n}{e}_{ih}^{2})({\sum }_{i=1}^{n}{e}_{ik}^{2})}}$$

where *e*_*ih*_ and *e*_*ik*_ are obtained by applying eq. (3) to the *h*^*th*^ and the *k*^*th*^ blood parameter level.

The *p*-value was computed by using a t-distribution (*T*) with *n* − 2 degrees of freedom, as follows:

6 $$p=2\times P(T > t)$$

where the test statistic *t* is given by:

7 $$t=\frac{r\times \sqrt{n-2}}{\sqrt{1-{r}^{2}}}$$

with *n* equal to the sample size (*n* = 843). Bonferroni correction was applied considering a nominal level of α = 0.05 and a total of $$(\begin{array}{c}m\\ 2\end{array})$$ correlation coefficients. This resulted in a threshold value of *p*-value = 1.15 × 10^−4^ corresponding to a minimum |*r*| = 0.133. However, the network resulted highly connected, so we retained only correlation coefficients with |*r*| > 0.4 (medium correlation). Correlation coefficients and significances were computed in R with the function *cor*.*test*. The network was visualized and annotated with Cytoscape 3.0^40^.

#### Genome-wide association analyses

Three methods (described in detail below) were used for genome-wide association analyses. Before fitting the genome-wide association models, the effect of slaughtering date on each normalized blood parameter was removed by obtaining residuals from a linear regression model.

1. **Single-marker single-trait genome-wide association analysis**
  Genome-wide association studies were performed examining each trait-SNP pair, hereafter denoted as (*j*, *i*), where *j* = 1, …, *q* (*q* = 30) and *i* = 1,…*p* (*p* = 45,536). Additive genetic models assuming a trend per copy of the minor allele were used to specify the dependency of each blood parameter on genotype categories. The following linear mixed effect model was specified:
  8 $${\boldsymbol{y}}=W{\boldsymbol{\alpha }}+{\boldsymbol{x}}\beta +{\boldsymbol{g}}+{\boldsymbol{e}}$$
  where ***y*** (*n* × 1) is a vector containing blood parameter (residuals of the normalized blood parameter) for the *n*^th^ animal, ***W*** (*n* × *k*) is a covariate matrix with *k* = 3 (a column of 1 s, sex, and weight) and **α** is the *k*-dimensional vector of covariates effects, ***x*** (*n* × 1) is the vector containing genotypes for the i^th^ SNP (coded as 0, 1, 2, according to the number of copies of the minor allele), *β* is the additive fixed effect of the *i*^th^ SNP on blood parameter, **g**~N(**0**,σ^2^_g_ **K**) is a multivariate Gaussian polygenic effect, with covariance matrix proportional to the relatedness matrix **K** (*n* × *n*) and **e**~N(**0**,σ^2^_e_ **I**) is a multivariate Gaussian vector of uncorrelated residuals. The assessment of the association between each SNP and blood parameter was obtained by testing the null hypothesis H_0_:β = 0. Significance was tested by using the Wald test. All the models were fitted with GEMMA^41^ after computing the relatedness matrix **K** as a centred genomic matrix (this matrix provides a good control for population structure^41^). To account for multiple comparisons, we opted for the Bonferroni correction, which considered a total 45,536 SNPs, 30 phenotypes and a value of α = 0.05. We estimated a threshold of $p-value=\frac{0.05}{45,536\times 30}=3.66\times {10}^{-8}$. However, the Bonferroni correction assumes independence among the performed tests, so that it is inherently conservative when applied to correlated phenotypes and genetic data that exhibits high linkage disequilibrium^42,43^. Therefore, to take in consideration also moderate associations, and balance the risk of Type I and Type II errors, in our analyses we considered a less conservative significance threshold of *p*-value = 5.0 × 10^−05^, as widely adopted in genome-wide association studies in farm animals (i.e^2,37,44–47^). Based on this threshold, for a QTL region related to the analysed trait, the SNP with the lowest *p*-value was considered as a “tag” SNP. Given the presence of multiple peaks on the same chromosome, tag SNPs for the trait “basophil count” (BASO) were identified by using Haploview^48^.
  The proportion of variance in phenotype explained by a given SNP (PVE) was computed as described in^49^. Briefly, PVE was estimated as follows:
  9 $$PVE=\,\frac{2{\hat{\beta }}^{2}\times MAF\times (1-MAF)}{2{\hat{\beta }}^{2}\times MAF\times (1-MAF)+{(se(\hat{\beta }))}^{2}+2N\times MAF\times (1-MAF)}$$
  where:
  $\hat{\beta }$, $se(\hat{\beta })$ and *MAF* are the effect size estimate, the standard error of the effect size estimate and the minor allele frequency of a given SNP, respectively. *N* represents the sample size. GEMMA was used also to retrieve, for each trait, the chip heritability (or SNP heritability; ${h}_{SNP}^{2}$) estimated by the whole set of available genotypes.
  QQplots and Manhattan plots were generated in R by using the “qqman” package.
2. **Multivariate genome-wide association analysis**
  Multivariate genome-wide association (single-marker multi-traits) studies were performed with GEMMA by fitting the following multivariate linear mixed model:
  10 $${\boldsymbol{Y}}={\boldsymbol{WA}}+{\boldsymbol{\beta }}{{\boldsymbol{x}}}^{T}+{\boldsymbol{G}}+{\boldsymbol{E}}\,{\bf{G}}\,\sim M{N}_{n\times d}({\bf{0}},{{\boldsymbol{V}}}_{g},{\boldsymbol{K}})\,{\bf{E}}\,\sim M{N}_{n\times d}({\bf{0}},{{\boldsymbol{V}}}_{e},{\boldsymbol{I}})$$
  where ***y*** (*n* × *d*) is a matrix containing *d* blood parameters (residuals of the normalized parameters) for the *n* animals, ***W*** (*n* × *k*) is a covariate matrix with *k* = 3 (a column of 1 s, sex, and weight) and ***A*** (*k* × *d*) is the matrix of the corresponding coefficients including the intercept, ***x*** (*n* × 1) is the vector containing genotypes for the i^*th*^ SNP (coded as 0, 1, 2, according to the number of copies of the minor allele), ***β*** is the additive fixed effect of the *i*^th^ SNP for the *d* phenotypes, ***G*** (*d* × *n*) is a matrix of random effects, ***E*** (*d* × *n*) is matrix of residual errors, ***K*** (*n* × *n*) is the relatedness matrix, **I** (n × *n*) is the identity matrix, **V**_g_ (*d* × *d*) is the symmetric matrix of genetic variance component, **V**_e_ (*d* × *d*) is a symmetric matrix of environmental variance component and MN_d×n_(**0**, **V**_**1**_, **V**_**2**_) denotes the *d* × *n* matrix normal distribution with mean 0, row covariance matrix **V**_**1**_ (*d* × *d*), and column covariance matrix **V**_**2**_ (*n* × *n*). Association between each SNP and blood parameters was obtained by testing the null hypothesis H_0_:β = 0. Wald test was used to test association significance. As relatedness matrix ***K*** we computed with GEMMA a centred genomic matrix. Significant threshold, QQ plots and Manhattans plots were defined or produced as reported for the single-marker single-trait analyses.
3. **Bayesian genome-wide association analysis (windows-based scan)**

As a first step for this approach, missing genotypes were imputed with Beagle v.4.1^50^. Bayesian analyses were performed with GenSel v.4^51^, using the method Bayes-C. This method uses all SNPs simultaneously and assumes a common variance for all SNPs. The model was as follows:

11 $${\boldsymbol{y}}={\boldsymbol{Wb}}+\sum _{j=1}^{J}{{\boldsymbol{x}}}_{j}{\beta }_{j}{\delta }_{j}+{\boldsymbol{e}}$$

where ***y*** (*n* × 1) is a vector containing blood parameter (residuals of the normalized parameter) for the *n*^th^ animal, ***W*** (*n* × *k*) is a covariate matrix with *k* = 4 (sex, weight and two columns for relatedness), ***b*** is the vector of fixed effects, *J* is the number of SNPs, ***x***_*j*_ is the vector containing genotypes for the SNP_j_ (coded as 0, 1, 2, according to the number of copies of the minor allele), *β*_*j*_ is the random substitution effect for the *j*^*th*^ SNP, which conditional on *σ*^2^_*β*_ was assumed normally distributed *N* (0, σ^2^_*β*_) when *δ*_*j*_ = 1 but *β*_*j*_ = 0 when *δ*_*j*_ = 0, with *δ*_*j*_ being a random 0/1 variable indicating the absence (with probability *π*) or presence (with probability 1 − *π*) of SNP *j* in the model, and ***e*** is the vector of the random residual effects assumed normally distributed N (0, σ^2^_e_). Relatedness was accounted by considering the first two eigenvectors of the centred genomic relatedness matrix ***K***, as computed in R by using the function “*eigen*”. The Markov Chain Monte Carlo (MCMC) method, as implemented in GenSel, was used to obtain the posterior distributions of SNPs effects. This comprised a burn-in period of 1,000 iterations from which results were discarded, followed by 51,000 iterations from which results were accumulated to obtain the posterior mean effect of each SNP^28,47^. In the Bayesian variable selection, multiple-regression models with *π* = 0.995, about 200–250 SNPs were fitted simultaneously in each MCMC iteration. The cumulative effect of markers within 1 Mb non-overlapping genome windows was computed in GenSel. The window effect was expressed as the percentage of total genetic variance contributed by each window^28^. A total of 2,332 genomic windows were retrieved. The expected percentage of genetic variance explained by each genomic window (%Var) was equal to 0.043% (100/2,332). The genomic windows explaining more than the 0.043% of genetic variance were considered non-random associations. However, a more stringent and reliable threshold, ranging from 0.2 to 1.00 (which means from ~5X to ~25X the expected variance), is generally applied to consider as QTLs the identified regions, with a threshold value of %Var = 0.5 commonly adopted^28,47,51–55^. Here, a medium moderate threshold, equal to 0.7 (~17X the expected variance), was used: (i) to declare the presence of QTLs and (ii) to confirm those identified via the single-marker single-trait approach. Manhattan plots representing the proportion of genetic variance of consecutive non-overlapping genomic windows were obtained with the “qqman” R package.

### Functional annotation of QTL regions and comparative analysis with previous studies

Different QTL regions for a specific parameter were defined considering significant SNPs that mapped at least one Mbp apart from another significant SNP. Functional analysis was carried out by (i) retrieving all annotated genes in Sscrofa11.1 from a region spanning ±1 Mbp the significant SNPs that identified the borders of that QTL and by (ii) evaluating the relevance of the genes in affecting the considered parameter through the scientific literature, selected combining as keywords the name of the gene under analysis (or synonyms) and the parameter itself (including alias and related traits). In addition, traits (or groups of similar traits defined by the correlation network analysis; see above) for which at least five QTLs were identified, gene enrichment analysis was carried out with NET-GE (http://net-ge.biocomp.unibo.it/enrich)^56^. NET-GE took as input all genes in the QTL regions or only the closest gene to the most significant SNP or only preselected genes based on their functions (as defined above), including at least one gene per QTL region. Over-representation analysis was carried out considering statistically enriched terms with a *p*-value < 0.05, Benjamini-Hochberg corrected (False Discovery Rate, FDR). Analyses run over the Gene Ontology (http://geneontology.org/), KEGG (http://www.kegg.jp/) and Reactome (https://reactome.org/) databases.

Comparative QTL mapping analysis across studies was obtained using the pigQTL database (https://www.animalgenome.org/cgi-bin/QTLdb/SS/index)^57^ with direct evaluation on the related published literature in pigs.

## Results

### Blood parameters and networks

A total of 843 Italian Large White pigs were analysed for 30 blood-related parameters: 15 haematological traits and 15 clinical-biochemical parameters. Descriptive statistics for all these traits are reported in Supplementary Table S1.

**Figure 1:**
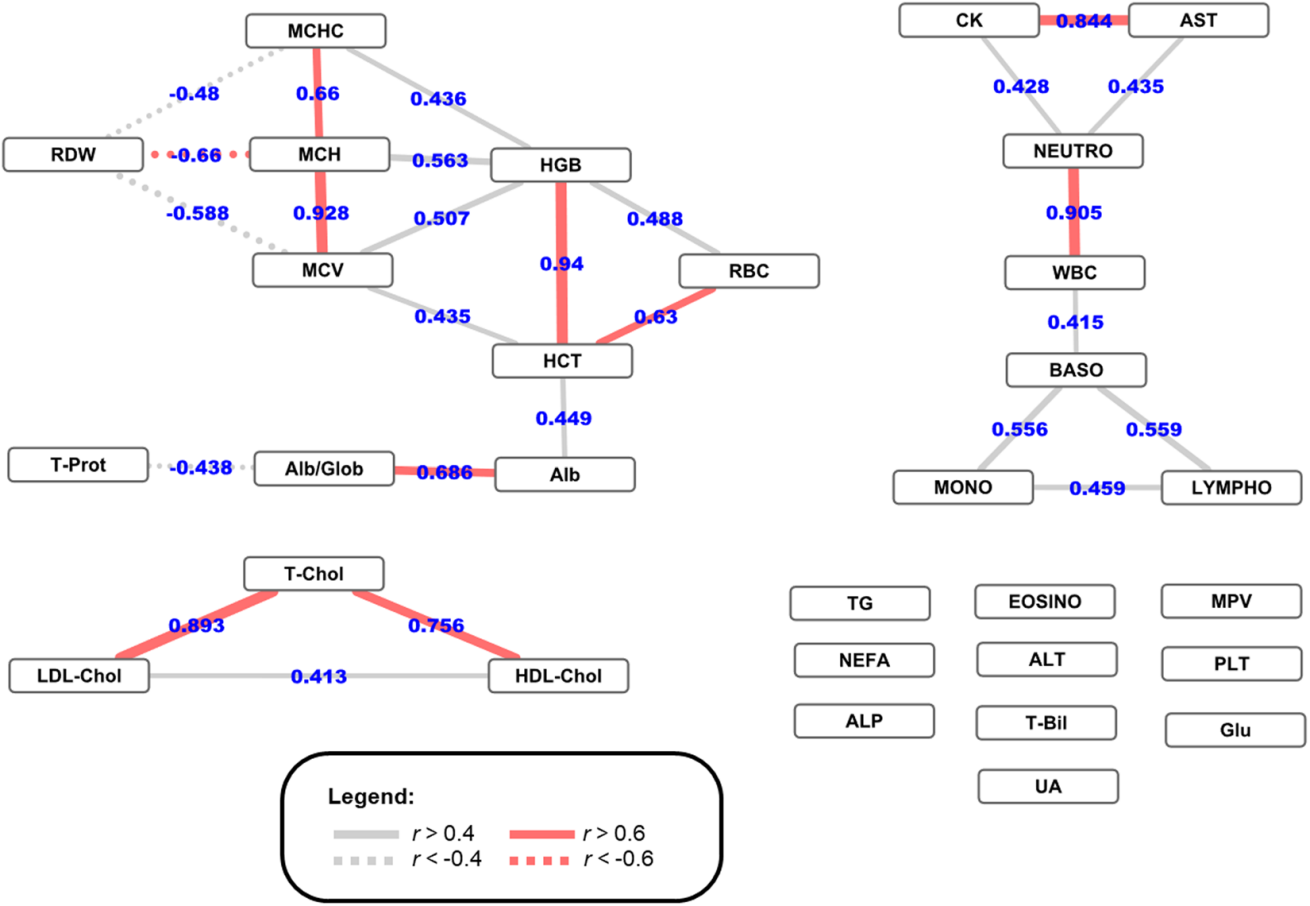
Network representing the Pearson’s correlation coefficients among the 30 analysed blood parameters. Only |*r*| > 0.4 correlations coefficients are drawn (line thickness represents the correlation strength).

A correlation network based on Pearson’s correlation coefficients was used to study the dependence among these parameters. The modelling resulted in a network of 30 nodes (of which 10 were singletons) and 27 edges (|*r*| > 0.4; Fig. 1). This network was characterized by modules describing more complex traits, such as erythropoiesis or leukopoiesis. Three clusters emerged: (i) a large module evidencing two sub-clusters, one erythrocyte/platelet-related [RBC–HCT–HGB–MCV–MCHC–MCH–RDW] and one protein-related [T-prot–Alb– Alb-Glob-ratio]; (ii) a medium module highlighting two sub-clusters, one leukocyte-related [WBC–LYMPHO–MONO–BASO–NEUTRO] and one characterized by enzymatic parameters [AST–CK] and (iii) a small module comprising three lipid-related blood parameters [T-Chol–LDL-Chol–HDL-Chol].

Based on network analysis results, a few medium/high correlated (|*r*| > 0.6) haematological trait clusters were identified and subsequently used in the multivariate genome-wide association scan. In total, six modules were defined: (1) [AST–CK], (2) [NEUTRO–WBC], (3) [T-Chol–LDL-Chol–HDL-Chol], (4) [Alb–Alb-Glob-ratio], (5) [RBC–HGB–HCT] and (6) [MCH–MCHC–MCV–RDW].

### Genome-wide analyses

The proportion of phenotypic variance (PVE%) explained by SNPs (single-marker single-trait analysis) ranged from 3.8 for BASO, to 9.0 for LDL-Chol (Supplementary Table S2). Heritability estimates for the analysed traits showed an average value of ${h}_{SNP}^{2}=0.29$, with the highest values for the two enzyme traits AST and ALT, of ${h}_{SNP}^{2}=0.44$ and ${h}_{SNP}^{2}=0.43$, respectively (Supplementary Table S1). The average ${h}_{SNP}^{2}$ Standard Error (SE) associated to heritability estimates was equal to 0.060 and ranged from 0.028 to 0.071 (Supplementary Table S1).

Genome-wide association analyses were carried out using three approaches (single-marker single-trait analysis, multivariate analysis and Bayesian analysis). The single-marker single-trait approach could account for the population structure of the pigs of the sib-testing program^29,30^. Bayesian analysis was applied to overcome the multiple testing correction to declare significant results in an experimental design in which the power was limited by the number of animals for which blood traits could be measured^30^. Lastly, because of the similarity of several blood phenotypes, the multivariate approach was carried out to boost the power of genome scans by detecting pleiotropic loci^31^.

In the single-marker single-trait analysis, a total of 51 unique tag SNPs was associated to all the 15 haematological traits and to 12 out of 15 clinical-biochemical traits investigated in this work. Table 1 includes only the top/tag markers associated with the investigated traits. Supplementary Table S2 reports all the identified SNPs (no. = 190) associated to these blood-related phenotypes.

**Table 1:** Tag single nucleotide polymorphisms (SNPs) identified in the single-marker single-trait genome-wide association studies for the 30 blood parameters.

Trait^a^	SSC^b^	Marker	Position (bp)^c^	m/M alleles^d^	MAF^e^	*PVE(%)* ^*f*^	*p-*value	Closest protein coding gene (kbp distance)	Previous studies^g^
**Haematological traits**									
**Erythrocyte traits**									
RBC	5	M1GA0007649	15,060,402	G/A	0.122	4.0	3.33 × 10^−05^	*KMT2D* (0)	—
HGB	13	CASI0007727	177,262,726	G/A	0.071	5.2	2.16 × 10^−06^	*ROBO2* (117)	—
	18	DRGA0017710	36,749,906	G/A	0.402	4.2	2.15 × 10^−05^	*ELMO1* (5)	—
HCT	18	DRGA0017710	36,749,906	G/A	0.401	4.1	2.37 × 10^−05^	*ELMO1* (5)	—
MCV	5	MARC0069472	14,820,993	C/A	0.077	4.3	1.63 × 10^−05^	*CCNT1* (13)	—
	8	DRGA0008367	17,423,203	G/A	0.329	4.0	2.97 × 10^−05^	*GBA3* (410)	—
MCH	5	MARC0069472	14,820,993	C/A	0.077	4.9	3.86 × 10^−06^	*CCNT1* (13)	—
	14	H3GA0038580	6,432,121	A/G	0.076	4.9	3.45 × 10^−06^	*LGI3* (3)	—
MCHC	5	MARC0044114	85,947,536	G/A	0.233	4.5	9.05 × 10^−06^	*TMPO* (601)	—
	14	H3GA0038597	6,641,352	G/A	0.351	3.9	3.56 × 10^−05^	*SLC39A14* (0)	—
RDW	12	DIAS0000242	40,010,805	A/G	0.284	3.9	3.65 × 10^−05^	*UNC45B* (0)	^22^
	16	ALGA0090171	33,206,877	G/A	0.367	4.9	3.69 × 10^−06^	*ARL15* (0)	—
	16	ALGA0091410	67,367,349	A/G	0.087	4.3	1.51 × 10^−05^	*SGCD* (0)	—
**Leukocyte traits**									
WBC	6	DIAS0004496	97,994,715	A/G	0.062	3.9	4.27 × 10^−05^	*APCDD1* (0)	—
LYMPHO	2	DIAS0001270	3,257,622	A/G	0.170	3.9	4.10 × 10^−05^	*ANO1* (0)	—
NEUTRO	4	MARC0052177	77,597,154	G/A	0.415	3.9	4.27 × 10^−05^	*RB1CC1* (0)	—
EOSINO	3	H3GA0009277	35,094,559	C/A	0.225	3.9	3.69 × 10^−05^	*RBFOX1* (0)	—
	3	H3GA0010692	117,043,080	A/C	0.250	3.9	4.28 × 10^−05^	*TDRD15* (139)	—
	7	H3GA0021970	61,362,991	C/A	0.281	4.1	2.60 × 10^−05^	*SEC*. *23 A* (179)	—
	7	INRA0028736	118,053,616	G/A	0.099	4.7	6.47 × 10^−06^	*VRK1* (28)	—
	10	H3GA0030197	46,331,882	G/A	0.194	4.1	2.42 × 10^−05^	*ITGA8* (28)	—
BASO	14	ALGA0079529^#^	71,958,965	A/G	0.114	7.5	6.53 × 10^−09^	*STOX1* (0)	LYMPHO^28^
	14	MARC0090899^§^	72,704,456	A/G	0.113	7.5	3.07 × 10^−09^	*FAM241B* (55)	—
MONO	15	ALGA0084320	19,853,411	A/G	0.145	4.6	6.58 × 10^−06^	*GPR39* (52)	—
**Platelet traits**									
PLT	1	ALGA0001781	24,412,234	A/G	0.403	4.3	1.73 × 10^−05^	*CITED2* (743)	—
	4	MARC0047043	94,795,752	G/A	0.255	4.2	1.77 × 10^−05^	*ZBTB7B* (0)	—
MPV	12	ASGA0053310	13,483,964	A/G	0.282	3.9	3.54 × 10^−05^	*CACNG1* (2)	—
**Clinical-chemical traits** ^**b**^									
**Lipid related traits**									
T-Chol	3	DIAS0000055	117,295,071	A/G	0.473	7.5	8.03 × 10^−09^	*APOB* (0)	^33, 90, 91^
	4	ALGA0022970	9,713,080	C/A	0.490	4.2	2.11 × 10^−05^	*ADCY8* (0)	—
	5	ASGA0104003	80,770,124	C/A	0.236	4.4	1.18 × 10^−05^	*STAB2* (0)	^6^
	7	MARC0003814	17,166,311	A/C	0.202	3.9	3.94 × 10^−05^	*SOX4* (271)	—
LDL-Col	1	ALGA0004272	72,306,533	G/A	0.110	4.8	5.14 × 10^−06^	*PRDM1* (3)	—
	1	ALGA0008284	228,918,979	A/G	0.473	4.7	6.05 × 10^−06^	*PCSK5* (0)	—
	3	ASGA0013487	12,471,350	A/G	0.465	4.1	2.24 × 10^−05^	*CASTOR2* (431)	—
	3	DIAS0000055	117,295,071	A/G	0.473	9.0	1.71 × 10^−05^	*APOB* (0)	^33, 90, 91^
	14	ALGA0077250	43,459,397	A/G	0.482	4.1	2.58 × 10^−05^	*MYO18B* (8)	—
TG	16	ASGA0073326	47,077,521	A/G	0.468	3.9	3.53 × 10^−05^	*LOC110257255* (202)	—
NEFA	2	ALGA0015164	115,449,913	G/A	0.338	4.1	2.25 × 10^−05^	*TMEM232* (0)	—
	9	ASGA0041146	5,738,251	G/A	0.146	4.1	2.37 × 10^−05^	*LOC100517176* (2)	—
	14	DRGA0013970	64,191,357	A/C	0.079	4.6	8.83 × 10^−06^	*LOC11025682*2 (0)	—
**Metabolism and Protein related traits**									
Glu	7	ALGA0110857	101,154,686	A/G	0.408	4.2	2.08 × 10^−05^	*NRXN3* (0)	—
UA	5	ALGA0031618	31,257,070	A/G	0.304	4.0	3.07 × 10^−05^	*GRIP1* (82)	—
	5	ALGA0031630	31,476,339	C/A	0.304	4.0	3.07 × 10^−05^	*CAND1* (181)	—
	9	ALGA0051311	10,886,506	A/G	0.300	4.0	3.33 × 10^−05^	*ENSSSCG00000031998* (49)	—
T-Bil	14	ASGA0067171	130,438,475	G/A	0.488	4.1	2.61 × 10^−05^	*PLPP4* (14)	—
	17	ALGA0094849	37,006,714	A/G	0.200	4.2	2.09 × 10^−05^	*CBFA2T2* (0)	—
T-Prot	17	M1GA0022271	52,958,671	A/G	0.263	4.1	2.62 × 10^−05^	*ATP9A* (0)	—
Alb	6	H3GA0052531	88,193,827	A/G	0.460	3.9	4.37 × 10^−05^	*COL16A1* (0)	—
Alb-Glob-ratio	5	H3GA0015245	5,996,798	A/G	0.388	4.3	1.59 × 10^−05^	*ARFGAP3* (0)	^6^
**Enzyme traits**									
ALT	4	ALGA0029783	1,175,147	G/A	0.131	4.8	4.99 × 10^−06^	*TOP1MT* (0)	—
	NW_018084979.1	H3GA0023887	3,163,232	G/A	0.373	4.9	3.88 × 10^−06^	*PLD4* (0)	—
	18	ALGA0098672	48,131,574	A/G	0.231	4.4	1.11 × 10^−05^	*NPY* (138)	—
AST	14	INRA0046629	110,424,327	A/G	0.320	4.4	1.15 × 10^−05^	*HPSE2* (0)	^6, 99^

^a^Erythrocyte traits: Red blood cell count (RBC); Hemoglobin (HGB); Hematocrit (HCT); Mean corpuscular volume (MCV); Mean corpuscular hemoglobin (MCH); Mean corpuscular hemoglobin concentration (MCHC); Red cell distribution width (RDW). Leukocyte traits: White blood cell count (WBC); Lymphocyte count (LYMPHO); Neutrophil count (NEUTRO); Eosinophil count (EOSINO); Basophil count (BASO); Monocyte count (MONO). Platelet traits: Platelet count (PLT); Mean platelet volume (MPV). Metabolism and Protein related traits: Glucose (Glu); Urea (UA); Total bilirubin (T-Bil); Total proteins (T-Prot); Albumin (Alb); Albumin/Globulines ratio (Alb-Glob-ratio). Lipid related traits: Total cholesterol (T-Chol); High-density lipoprotein cholesterol (HDL-Chol); Low-density lipoprotein cholesterol (LDL-Chol); Triglycerides (TG); Non-esterified fatty acids (NEFA). Enzyme traits: Alkaline phosphatase (ALP); Creatine kinase (CK); Alanine aminotransferase (ALT); Aspartate aminotransferase (AST).

^b^SSC = *Sus scrofa* chromosome.

^c^Position (bp): position based on the Sscrofa11.1 reference genome.

^d^m/M Allele = minor/major allele.

^e^MAF = Minor Allele Frequency.

^f^PVE(%): proportion of variance in phenotype explained by the SNP.

^g^References and traits (if different from that of the corresponding QTL) that showed QTLs in the same chromosome region. The symbol “-” indicates that no QTLs have been reported for the same or similar traits in that region.

^#^Top tag SNP selected with Haploview.

^§^Top associated SNP.

The Bayesian analysis (windows-based single-trait) reported a total of 22 windows with an explained additive genetic variance >0.7% each, for twelve different traits (Table 2). Fifteen of these 22 windows (~70%) overlapped significant SNPs reported in the single-marker analysis.

**Table 2:** Top genomic windows identified in the Bayesian genome-wide association studies (windows-based single trait approach) for the 30 blood parameters.

Trait^a^	SSC^b^	Start^c^	End^d^	Mbp^e^	SNPs^f^	% Var^g^	Genes^h^	Previous studies^i^
**Haematological traits**								
BASO	14	67,039,033	67,985,525	0.95	25^#^	1.36	*REEP3*	LYMPHO^28^
HCT	7	50,022,514	50,996,708	0.97	34	0.93	*IL16; STARD5; TMC3; LOC102160759; MEX3B; EFL1; SAXO2*	—
HGB	7	50,022,514	50,996,708	0.97	34	1.3	*IL16; STARD5; TMC3; LOC102160759; MEX3B; EFL1; SAXO2*	—
	7	52,033,314	52,890,898	0.86	24	0.71	*HOMER2; WHAMM; FSD2; AP3B2; CPEB1; RPS17; PDE8A; SLC28A1; ALPK3; ZNF592; SEC*. *11 A; NMB; WDR73*	—
	18	36,020,050	36,959,194	0.94	16^#^	0.94	*DNAJB9; THAP5; GPR141; ELMO1*	—
MCH	14	6,142,147	6,991,699	0.85	25^#,§^	0.72	*DOK2; XPO7; NPM2; FGF17; DMTN; LOC110256689; LOC110256690; FAM160B2; NUDT18; HR; REEP4; LGI3; SFTPC; BMP1; PHYHIP; POLR3D; PIWIL2; SLC39A14; PPP3CC; SORBS3; PDLIM2; CCAR2; BIN3; EGR3; PEBP4*	—
	14	8,994,489	8,994,023	0.99	27	0.72	*ADAM28; ADAMDEC1; ADAM7; NEFM; NEFL*	—
MCV	8	17,019,764	17,986,937	0.97	23^#^	1.43	*PPARGC1A*	—
PLT	1	24,020,779	24,830,003	0.81	20^#^	0.91	—	—
RBC	5	100,071,672	100,955,310	0.88	24	0.71	*PPFIA2; ACSS3; LIN7A; LOC110260827; MYF5; MYF6; PTPRQ*	—
	7	27,045,611	27,963,262	0.92	31	0.72	*KLHL31; GCLC; KHDRBS2*	—
**Clinical-biochemical traits**								
Alb	6	88,007,685	88,895,391	0.89	28^#^	1.16	*SERINC2; LOC100520618; TINAGL1; HCRTR1; PEF1; COL16A1; ADGRB2; SPOCD1; PTP4A2; KHDRBS1; TMEM39B; KPNA6; TXLNA; CCDC28B; IQCC; DCDC2B; TMEM234; EIF3I; LOC106510645; FAM167B; LCK; HDAC1; MARCKSL1; FAM229A; BSDC1; TSSK3; ZBTB8B*	—
ALT	18	48,008,832	48,983,680	0.97	20^#^	0.71	*STK31; FAM221A; UPP1; C18H7orf57; SUN3; HUS1; LANCL2; VOPP1; PGAM2; DBNL; UBE2D4; LOC100525140; MRPS24; TNS3*	
	NW_018084979.1	2,062,221	2,846,867	0.78	24	1.78	—	—
	NW_018084979.1	3,094,869	3,331,399	0.21	7^#^	1.06	—	—
HDL-Chol	4	9,035,765	9,963,475	0.93	21^§^	0.84	*OC90; EFR3A; ADCY8*	—
	9	42,015,102	42,977,109	0.96	29	0.95	*NXPE4; CADM1*	
LDL-Chol	1	228,174,388	228,987,611	0.81	15^#^	0.81	*OSTF1; PCSK5*	^33, 90, 91^
	3	12,025,480	12,941,569	0.92	27^#^	0.82	*CASTOR2*	—
	3	117,016,694	117,989,682	0.97	21^#^	5.05	*TDRD15; APOB; LDAH; GDF7; HS1BP3; RHOB; LOC100523732; PUM2; SDC1*	—
	14	43,074,710	43,957,703	0.88	31^#^	0.9	*KIAA1671; CRYBB3; CRYBB2; LOC110256814; GRK3; MYO18B; SEZ6L*	—
T-Chol	3	117,016,694	117,989,682	0.97	21^#^	2.77	*TDRD15; APOB; LDAH; GDF7; HS1BP3; RHOB; LOC100523732; PUM2; SDC1*	^33, 90, 91^
UA	5	31,022,891	31,954,627	0.93	28^#^	0.74	*GRIP1; CAND1*	—

^a^Full names are reported as note to Table 1.

^b^SSC = *Sus scrofa* chromosome.

^c^Start: position of the first SNP in the window.

^d^End: position of the last SNP in the window.

^e^Mbp: window size.

^f^SNPs: number of SNPs overlapping the window.

^g^% Var: proportion of genetic variance explained by the window.

^h^Genes: protein coding genes located within the window.

^i^References and traits (if different from that of the corresponding QTL) that showed QTLs in the same chromosome region. The symbol “−” indicates that no QTLs have been reported for the same or similar traits in that region.

^#^The window contains SNPs detected via the single-marker single-trait approach.

^§^The window contains SNPs detected via the single-marker multi-traits approach.

The single-marker multi-trait analysis reported a total of 13 tag SNPs associated to five out of six groups of haematological traits (Table 3; no significant associations were obtained for the [NEUTRO–WBC] cluster). Supplementary Table S3 reports all the identified SNPs (n = 49) associated to these clusters of blood parameters. Comparing each set of blood parameters against the related single-trait analyses, five SNPs were in QTL regions not highlighted by the other two single-trait methods (i.e. single-marker and Bayesian approaches).

**Table 3:** Tag single nucleotide polymorphisms (SNPs) identified in the multivariate genome-wide association scans for the six sets of blood parameters.

Trait^a^	SSC^b^	Marker	Position (bp)^c^	m/M alleles^d^	MAF^e^	p-value	QTL single-trait analysis^f^	Previous studies^g^
Alb–Alb-Glob-ratio	7	INRA0025193	38,259,165	A/G	0.406	2.80 × 10^−05^	**	—
AST-CK	2	MARC0016794	8,393,235	C/A	0.488	2.24 × 10^−05^	**	—
	14	INRA0046629	110,424,327	A/G	0.320	1.39 × 10^−13^	AST	^6, 99^
MCH–MCHC–MCV–RDW	5	MARC0069472	14,820,993	C/A	0.077	1.73 × 10^−06^	MCH, MCV	—
	7	ASGA0036974	120,968,439	G/A	0.082	5.50 × 10^−07^	**	—
	14	H3GA0038580	6,432,121	A/G	0.076	3.33 × 10^−05^	MCH, MCHC	—
RBC–HGB–HCT	5	MARC0069472	14,820,993	C/A	0.077	1.68 × 10^−05^	RBC	—
	13	CASI0007727	177,262,726	G/A	0.071	3.47 × 10^−05^	HGB	—
	14	H3GA0038580	6,432,121	A/G	0.076	3.29 × 10^−05^	**	—
T-Chol–LDL-Chol–HDL-Chol	1	ALGA0004272	72,306,533	G/A	0.110	3.75 × 10^−05^	LDL-Chol	—
	2	ALGA0013564	43,603,656	C/A	0.406	5.00 × 10^−05^	**	—
	3	ASGA0016313	116,718,459	G/A	0.145	5.24 × 10^−12^	LDL-Chol	^33, 90, 91^
	4	ALGA0022970	9,713,080	C/A	0.490	4.77 × 10^−05^	T-Chol, HDL-Chol	—

^a^Full names are reported as note to Table 1.

^b^SSC = *Sus scrofa* chromosome.

^c^Position (bp): position based on the Sscrofa11.1 reference genome.

^d^m/M Allele = minor/major allele.

^e^MAF = Minor Allele Frequency.

^f^The double star symbol (**) indicates a new discovered association detected by the multivariate genome scan (i.e. neither the single-marker approach nor the Bayesian one was able to highlight the QTL for the same traits). The remaining cases report the trait for which the QTL has been identified via the other two approaches.

^g^References that showed QTLs in the same chromosome region. The symbol “–” indicates that no QTLs have been previously reported for the same or similar traits in that region.

By combining results from the single-trait and the multivariate genome-wide association scans, a total of 52 unique QTL regions were identified (Supplementary Table S4). These regions were distributed on all autosomes except on porcine chromosome (SSC) 11. Four QTL regions were reported only by multi-trait groups, 41 were detected only by single-traits and the remaining seven were detected by both multi-trait and single-trait analyses. A total of 23 QTL regions were for haematological traits (for all 15 parameters), 28 were for clinical-biochemical traits (for all 14 parameters; ALP levels were not statistically associated to any genomic marker) and one (on SSC3, position 12–18 Mbp) was shared between haematological and clinical-biochemical parameters (LDL-Chol, T-Chol and EOSINO).

Information on QTLs identified by other studies on the same traits matching the same chromosome regions reported in this work is included in Tables 1–3. Only six out of 52 QTL regions have been identified by other studies for the same or similar traits (Tables 1 and 2).

**Figure 2:**
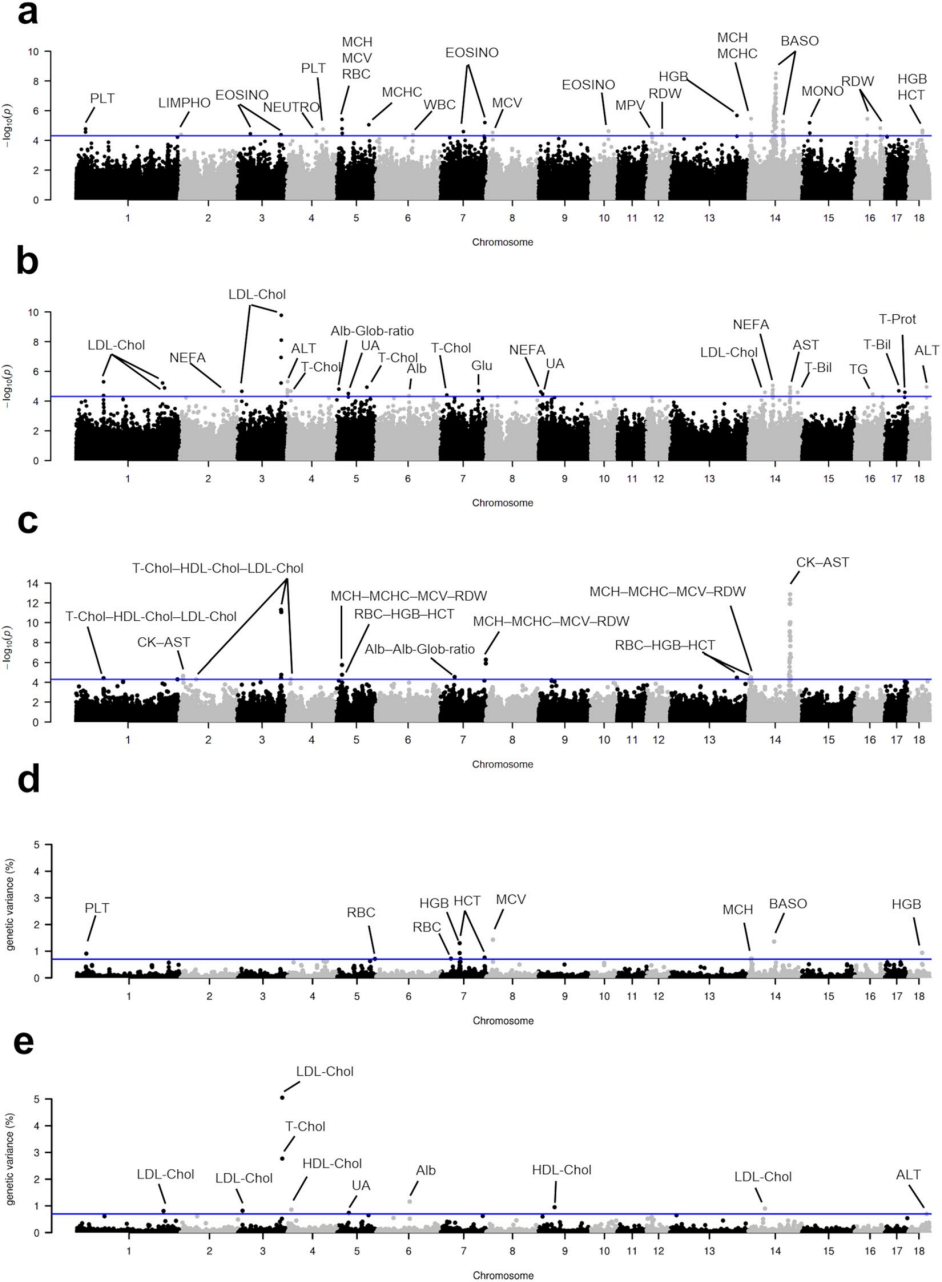
Over-imposed Manhattan plots displaying the results of the genome-wide association studies for the 30 blood parameters. (**a**) Single-marker single-trait genome-wide association scan for haematological traits; (**b**) Single-marker single-trait genome-wide association scan for clinical-biochemical traits; (**c**) Multivariate (single-marker multi-trait) genome-wide association scans for blood parameter clusters; (**d**) Bayesian genome-wide association scan (windows-based single-trait) for haematological traits; (**e**) Bayesian genome-wide association scan (windows-based single-trait) for clinical-biochemical traits.

Manhattan plots constructed over-imposing genome-wide association results for all considered haematological parameters and for all clinical-biochemical traits, carried out using single-marker single-traits linear models, multivariate scan and Bayesian (windows-based single-trait) analyses, are reported in Fig. 2. Manhattan plots produced separately for each trait are reported in Supplementary Fig. S1 (single-marker single-trait), Supplementary Fig. S2 (multivariate approach) and Supplementary Fig. S3 (Bayesian analysis). Q-Q plots are reported in Supplementary Fig. S4 and genomic inflation factors (λ) are included in Supplementary Table S5.

### Erythrocyte traits

A total of 21 SNPs/windows were identified for the seven investigated erythrocyte-related traits (in single-trait or multivariate analyses), with some SNPs/windows shared among the 30 analysed phenotypes: (i) two for RBC; (ii) three for HGB, one detected with the single-marker approach (single-trait and multi-trait) and two identified with Bayesian analysis); (iii) two for HCT; (iv) two for MCV, one of which (on SSC8) confirmed by the Bayesian method; (v) two for MCH, one of which (on SSC14) confirmed by all the three approaches; (vi) two for MCHC; (vii) three for RDW; (viii) three for the multi-trait group [MCH–MCHC–MCV–RDW], one of which not detected with the other single-trait methods and (ix) three for the multi-trait set [RBC–HGB–HCT], all of them detected also with the single-marker single-trait analysis. Combining all these QTLs, a total of 13 QTL regions (affecting one or more traits) were identified (Tables 1–3; Supplementary Table S4; Fig. 2).

Three traits (RBC, MCV and MCH), that quantify red blood cells (RBC) or define ratios including the number of red blood cells at the numerator or denominator of the formulas (see Supplementary Table S1), identified significant markers in the same SSC5 region (mapping at position ~15 Mbp). This region was also identified by the multivariate analysis of the two erythrocyte-related clusters, i.e. [RBC–HGB–HCT] and [MCH–MCHC–MCV–RDW], confirming the presence of a QTL region for erythrocyte traits. A few genes highly expressed in bone marrow (*ADCY6*, *TUBA1B*, *TUBA1C* and *KMT2D*), that could be considered candidates for red blood cell related parameters, are annotated in this chromosome region. Porcine chromosome 5 had another QTL for MCHC at position ~86 Mbp, close to the *TMPO* gene highly expressed in bone marrow.

Bayesian approach identified a QTL for HGB in a window on SSC7 (at position ~50 Mbp) that contains several annotated genes. One of which, *EFL1*, is ubiquitously expressed in bone marrow. Another gene is *ALPK3*, harbouring variants that have been associated in humans to the mean corpuscular haemoglobin concentration (MCHC)^58^. On the same chromosome, the multi-trait method located a QTL at position ~121 Mbp for the cluster [MCH–MCHC–MCV–RDW], not identified by the single-trait approaches. The Bayesian approach identified also a QTLs for RBC count on SSC7 (position 27–28 Mbp). This genomic region contains *GCLC*, *a* gene coding for the catalytic subunit of the glutamate-cysteine ligase (GCL). Mutations in the *GCLC* gene, causing GCL deficiency, have been linked to human haemolytic anaemia^59^.

A QTL for MCV was identified on SSC8 by the single-marker single-trait and Bayesian methods, centred on marker DRGA0008367 (position ~17.4 Mbp). An annotated gene included in the detected Bayesian window is *PPARGC1A*, which encodes for a regulator involved in fibre muscle type formation, blood pressure and cellular cholesterol homeostasis and fat deposition.

The RDW QTL identified on SSC12 (position ~40 Mbp) is marked by a SNP positioned within the *UNC45B* gene, whose known functions and restricted heart expression might not be related to RDW. A QTL on SSC13 for HGB positioned at ~177 Mbp (close to *ROBO2* gene) was also identified with the multi-trait [RBC–HGB–HCT] group.

Two QTLs were located on SSC14. One at position ~2.9 Mbp was only identified using the multi-trait analysis with the cluster [MCH–MCHC–MCV–RDW]. On the same chromosome, two related traits (MCH and MCHC) showed significant markers at position ~6.5 Mbp, close to *BIN3*, a gene reported to be highly expressed in bone marrow. Moreover, this genomic region contains *DOK2*, a gene which was suggested to regulate the differentiation of primitive erythrocytes in zebrafish embryos^60^, and *XPO7*, which was associated to the levels of MCV, MCH and RBC in humans^61,62^. This region was also confirmed by the [RBC–HGB–HCT] multi-trait set.

Two QTLs for RDW were mapped on SSC16 (one at position ~33.2 Mbp and one at position ~67.4 Mbp) with the single-marker single-trait analysis. Two QTLs were also reported for HCT on SSC7 and SSC18.

### Leukocyte traits

Ten QTLs were identified for the six leukocyte traits using the single-marker single-trait-approach, one of which (basophil count, on SSC14) was also identified with the Bayesian method (Tables 1 and 2).

Only one QTL was identified for each lymphocyte count (LYMPHO; on SSC2), neutrophil count (NEUTRO, on SSC4), white blood cell count (WBC; on SSC6), basophil count (BASO; on SSC14) and monocyte count (MONO; on SSC15). Several closest annotated genes have functions that might be indirectly linked to the reported QTLs. For example, the SNP that marked the NEUTRO QTL on SSC4 is within the *RB1CC1* gene, that is involved in autophagy biopathways. The basophil QTL region identified the largest number of significant markers (n. 101 of which 21 have been considered as tag SNPs), spanning a region of SSC14 from position ~66.4 to ~73.4 Mbp. In this region, the Manhattan plot (Fig. 2) evidences two close peaks potentially underlying two QTLs for this trait (but not completely separated to formally consider them two distinct QTL regions). This large region also includes the highly significant tag SNP (ALGA0079529, *p*-value = 6.53 × 10^−09^) that is within the *STOX1* gene. *STOX1* encodes for a DNA binding protein involved in preeclampsia. Other genes whose function might better support their candidacy in affecting basophil number are embedded in this large SSC14 QTL region: *CCNC* that is involved in the regulation of human hematopoietic stem/progenitor cell quiescence^63^; *EGR2* that controls adaptive immune responses by temporally uncoupling expansion from T cell differentiation^64^; *LIN28*B that encodes for pluripotency factor implicated in driving a fetal hematopoietic program^65^; *JMJD1C* which encodes for a hematopoietic transcription factor and that human gene variants have been associated with WBC^66^.

Eosinophil count (EOSINO) showed five QTLs on four different chromosomes (two on SSC3, two on SSC7 and one on SSC10). Eosinophils have a key role in the allergic inflammatory response. Therefore, it is interesting to note that the first significant region for EOSINO on SSC3 is identified by a SNP that is within the *RBFOX1* gene, whose variability in humans has been associated with food allergy^67^.

### Platelet traits

Two platelet traits have been investigated in this work showing a total of three QTLs, two for PLT (on SSC1, also detected with the Bayesian approach, and on SSC4) and one for MPV (on SSC12). The identified regions contain several genes whose known functions can be indirectly considered to have a putative role in platelet activity, level or development. For example, the PLT significant marker on SSC4 is within the *ZBTB7B* gene that encodes for a key regulator of lineage commitment of immature T-cell precursors, involved in several other functions.

### Lipid related traits

Five QTLs were reported for LDL-Cholesterol (LDL-Chol; two on SSC1, two on SSC3 and one on SSC14), four for Total-Cholesterol (T-Chol; on SSC3, SSC4, SSC5 and SSC7) and two for HDL-Cholesterol (HDL-Chol; on SSC4 and SSC9). The QTL on SSC3 controlling the levels of T-Chol was the same of LDL-Chol. The same marker (DIAS0000055, at position 117,295,071 on SSC3) was highly significant for both traits (LDL-Chol, *p*-value = 1.71 × 10^−10^; T-Chol, *p*-value = 8.03 × 10^−09^). This SNP is positioned within the *APOB* gene, which encodes for the main apolipoprotein of low-density lipoproteins and chylomicrons. Mutations in the human *APOB* gene cause familial hypercholesterolemia that is characterized by pathogenic elevated LDL-cholesterol levels and atherosclerosis (e.g.^68^).

Significant SNPs identifying other LDL-Chol and T-Chol QTLs were very close to or within genes that, based on their functions, might be directly involved in affecting cholesterol related traits. For example, ALGA0004272 (SSC1 at position ~72.3 Mbp), that identified a QTL for LDL-Chol, is close to the *ATG5* gene (position 72,345,004–72,520,448) which is one of the genes required for formation of the autophagic isolation membrane and engulfment. Macrophage specific *Atg5*-deficient mice were demonstrated to have decreased cholesterol efflux and for this reason the encoded protein is considered a key element in affecting cholesterol level in blood^69–71^. Another QTL for LDL-Chol on SSC1 identified by SNP ALGA0008284 (position ~228.9 Mbp) indicates the *PCSK5* gene as the most plausible candidate (position 228,854,608–229,308,906; including the mentioned marker). Genetic variations in the human *PCSK5* were shown to modulate high-density lipoprotein cholesterol levels with impact on other cholesterol fractions^71^.

The *ADCY8* gene, that is involved in a metabolic pathway associated with high-density cholesterol in human cohorts^72^, was tagged by ALGA0022970 (positioned within the porcine *ADCY8* on SSC4). This marker was associated with T-Chol (single-marker single trait) and HDL-Chol (windows-based) in our study. For the same trait, we identified a QTL on SSC7, tagged by a SNP (MARC0003814; position ~17.2 Mbp) that is close to two candidate genes: *CDKAL1* (position 15,910,494–16,674,989) and *PRL* (position: 17,449,586–17,463,970). Variants in the human *CDKAL1* gene have been associated with cholesterol efflux capacity^73^. The *PRL gene*, coding for the hormone prolactin, is well known to regulate cholesterol stores in male and female gonads and plasma total cholesterol concentration (e.g.^74^).

The multi-trait analysis of the three cholesterol-related traits, i.e. T-Chol, LDL-Chol and HDL-Chol, strengthened the association of the marker DIAS0000055 in the *APOB* gene (*p*-value = 8.55 × 10^−12^) and confirmed the QTLs on SSC1 (first region) and SSC4 and thus the potential involvement of the *ATG5* and *ADCY8* genes in affecting blood cholesterol level. Moreover, the multi-trait analysis for the same cholesterol traits highlighted a new QTL on the SSC2 (ALGA0013564, position ~43.6 Mbp) near the *SOX6* gene (position 42,452,778–43,068,323). *A*n *in vivo* mouse study demonstrated that *Sox6* is involved in the regulation of serum and liver triglyceride as well as serum cholesterol levels^75^.

Three NEFA QTLs were reported in three different porcine chromosomes (SSC2, SSC9 and SSC14). The SSC2 marker associated with NEFA level is close to the *SLC25A46* gene (position 115,529,496–115,555,452) which encodes for a member of the mitochondrial solute carrier family involved in several metabolic pathways, including fatty acid oxidation^76^. The QTL on SSC9, identified with a marker at position ~5.7 Mbp, is close to the *TRIM2*1 gene (position 5,783,322–5,789,812 bp) which regulates the acetylated form of the fatty acid synthase (*FASN*), a key enzyme in the fatty acid pathway^77^. The significant marker on SSC14 is close to a few genes (*CDK1*, *PSMA4* and *RHOBTB1*) whose role might indirectly affect blood content of NEFA.

Genes annotated in the SSC16 region in which a QTL for blood triglyceride (TG) levels was assigned did not have any obvious or known functions that could be related to this biochemical trait.

### Metabolism and protein related traits

A QTL for glucose content was identified with a marker on SSC7 (ALGA0110857) close *NRXN3* gene (position 101,132,780–102,779,809) which has been associated with human obesity and energy balance^78^.

QTLs for albumin content and albumin/globulin ratio mapped on SSC6 and SSC5, respectively, were close to genes involved in albuminuria (*FABP3*, SSC6 positions 87,941,137–87,951,610) and renal disfunctions (*PACSIN2*, SSC5 positions 5,823,811–5,985,173), respectively^79,80^. The QTL on SSC6 was also confirmed by the Bayesian scan.

Several genes were within (i) the two QTL regions (on SSC5 and SSC9) identified for urea blood content, (ii) the QTLs identified on SSC14 and SSC17 for total bilirubin content, (iii) the SSC6 QTL for total blood protein and (vi) the SSC7 QTL for the Albumin–Albumin-Globulin-ratio cluster. However, their known functions could not be attributed to a direct role on the observed effects.

### Enzyme traits

No markers were associated with alkaline phosphatase (EC 3.1.3.1, ALP), even if some SNPs on SSC2 were just below the significant threshold.

Alanine aminotransferase [EC 2.6.1.2, ALT or ALT1; also known as alanine transaminase (AAT1) or glutamate-pyruvate transaminase (GPT)] activity showed three QTLs, one on SSC4, one on SSC18 and another one on an unassigned scaffold (NW_018084979.1; Table 1). The QTL on SSC4 was marked by a SNP (ALGA0029783, position ~1,16 Mbp) close to the porcine *GPT* gene (position 297,747–302,498 bp), supporting a direct role of the gene encoding the analysed enzyme in the identified QTL related to its function.

The activity of aspartate aminotransferase [EC 2.6.1.1, AST; also known as aspartate transaminase or serum glutamic oxaloacetic transaminase (sGOT)] was significantly associated with a marker on SSC14 (INRA0046629 at position ~110.4 Mbp) *GOT1* gene (position 110,608,422–110,635,901). *GOT1* encodes for the cytoplasmic form of the enzyme whose activity was also measured, supporting again a direct involvement of this gene in the identified QTL related to its function. AST showed a high degree of correlation (*r* = 0.844) with creatine kinase (CK), so these two parameters were jointly analysed in the multivariate GWA scan using the [AST–CK] cluster. Creatine kinase and AST are two enzymes mostly present in muscle cells whose increased level in serum is an indicator of muscle stress or damage, whereas a high AST level is considered to be derived from liver damage^81,82^. The multivariate analysis strengthened the association of the markers on the SSC14 (INRA0046629; from *p*-value = 1.15 × 10^−05^ of the single-marker single-trait analysis to *p*-value = 1.39 × 10^−13^ of the combined [AST-CK] analysis) highlighting a larger genomic region with significant markers (Supplementary Table S3). This region includes both the *GOT1* and *CPN1* genes (positions 111,181,224–111,225,685). Genetic variations in the human *CPN1* gene have been associated with plasma levels of both ALT and AST enzymes^83^. Moreover, variations in the human *CPN1* gene have been also associated with blood CK levels^12^.

Another [AST-CK] QTL was located on SSC2 (position ~8.3–8.4 Mbp) but in this region no annotated genes might be directly involved in functions producing altered blood AST and/or CK levels.

### Gene enrichment

Gene enrichment analysis was performed with NET-GE, which performs both a standard and a network-based analysis (the latter taking advantage of protein-protein interaction networks to better define biological functions)^56,84^. When applied for traits for which at least five QTL regions were identified in the genome association analyses, NET-GE highlighted statistically significant terms when genes were preselected based on their functions for cholesterol related traits. A gene list identified as mentioned above (including *ADCY8*, *APOB*, *ATG5*, *CASTOR2*, *MYO18B*, *NPAP1*, *PCSK5*, *STAB2*, *SOX4* and *SOX6*) showed significant terms for the Gene Ontology Molecular Function (GO:MF) and the Reactome databases. The GO:MF highlighted four terms involving two genes of the input set. Two terms were leaves of the GO:MF hierarchy: low-density lipoprotein particle binding (GO:0030228, *p*-value = 0.012) and lipoprotein particle receptor activity (GO:0030169, *p*-value = 0.026). Over the Reactome database, two terms (involving four genes of the input set) were retrieved: (i) scavenging by Class H Receptors (R-HSA-3000497, *p*-value = 3.61 × 10^−04^), related to the vesicle-mediated transport, and (ii) deactivation of the beta-catenin transactivating complex (R-HSA-3769402, *p*-value = 0.025), related to a transduction pathway. Overall, these terms confirm the biological mechanisms underlying the identified QTLs related to blood cholesterol levels. For the other traits for which five or more QTLs were identified (i.e. eosinophil count and the multi-trait group [MCH–MCHC–MCV–RDW]) no significantly enriched terms were shown.

## Discussion

Sub-optimal farming practices and environmental stressors might reduce animal response to adverse conditions and increase disease susceptibility. To link physiological aspects of the animals to their potential production performances in many different conditions, intermediate (internal) phenotypes should be measured and used to describe the underlying fine biological mechanisms related to these aspects. In this context, blood parameters provide biomarkers with several applications in animal sciences: (i) to detect and monitor the pathophysiological status of the animals (diagnostic and monitoring biomarkers) and, in normal or challenging conditions, (ii) to indicate the potential to develop a disease or a sensitivity to an exposure (susceptibility/risk biomarkers), (iii) to identify animals that might experience a favourable or unfavourable effect from a particular condition or exposure (predictive biomarkers), and (iv) to identify likelihood of an adverse condition or disease progression^85^. Hematopoietic cells are responsible for a range of different functions including oxygen and dioxide transport (erythrocytes), immunosurveillance (leukocytes), clotting/homeostasis maintenance (platelets) and vary substantially among healthy animals. Lipids, energy related metabolites, proteins and enzyme activities in the serum are indicators of metabolic disorders, cardiovascular disease risks, oxidative stress, and many different pathological conditions (e.g.^86^).

Genetic variability in these blood parameters can be important to describe the potentials of the animals to cope with infective agents and stressing conditions. This information could contribute to design new strategies to overcome the limited effectiveness of the traditional selection programs to improve disease resistance, tolerance and resilience^87^. In addition, considering the importance of blood related traits in human medicine, results obtained in pigs might further strengthen the usefulness of this animal model in translational-biomedical applications for related aspects.

Despite the relevance of blood measures in animals, just few studies (compared to what has been reported for carcass and meat production and performance traits) have investigated at the genetic level these parameters in pigs. In this study, three different approaches for association analyses – single-marker single-trait, single-marker multi-trait (multivariate analysis) and multi-marker single-trait (Bayesian method) analyses – were adopted to dissect the genetic variability of 15 haematological traits (erythrocyte-, leukocytes- and platelet-related parameters) and 15 clinical-biochemical traits (lipids, metabolism and protein, and enzyme traits) in an Italian Large White heavy pig population. The use of different approaches was able to improve the output of this genome scan study, overcoming, at least in part, the limited power of the experimental design due to the small investigated population (blood traits were measured for a total of 843 animals). The multiple testing correction in the single-marker single-trait analysis should balance the risk of Type I and Type II errors and for this reason we applied a significance threshold that was already used in several other works (e.g.^2,37,44–47^) that needed to deal with this question that is quite common in livestock. To overcame in part this problem, we also applied another genome wide association method that used a windows-based approach (i.e. Bayesian approach). The use of genome windows could also counteract the problem of imperfect linkage disequilibrium established between markers nearby a gene affecting the QTL^30^. In addition to these two methods, a multivariate approach was added to the study with the objective to increase the statistical power and identify pleiotropic loci^31^, considering that several blood traits are correlated to each other (|*r*| > 0.4).

Similarly to other genome-wide association studies in livestock species (e.g.^27,28,32,47^), the combination of results derived by several genome-wide association approaches was able to refine and confirm QTL regions, as also observed in our study which highlighted genomic regions harbouring almost obvious candidate genes.

To our knowledge, this study in Italian Large White pigs reported results for the largest number of blood traits in any single genome-wide studies carried out in pigs so far. Most of the previous genome-wide studies investigated only cellular traits^18–28,88,89^ or clinical and biochemical parameters^6,7,33,34,90–92^. Results obtained by all these works showed few shared QTLs for the same traits (e.g.^33^). This is also what we obtained comparing the QTL maps for the same or similar traits already reported by others to the results we obtained in Italian Large White pigs. From a total of 52 QTLs that we identified in this heavy pig breed, only 10% were located in chromosome regions that have been already shown to harbour QTLs for the same or similar blood parameters included in the current work. This can be explained by the heterogeneity of the studies carried out so far (including animals analysed at different ages) which might reflect, in turn, the heterogeneity of the results: most previous works have studied QTLs in Asian breeds or crosses between Asian and European breeds and few works have been carried out on pure European breeds only. Large White pigs have been previously investigated in just one genome-wide association study as pure breed^89^ and in a F2 based study as parental animals^24^.

A few QTL regions showed pleiotropic effects, in particular for haematological traits. This could be expected since the different blood parameters (cell count, volumetric measurements or haemoglobin levels) can serve as intermediate descriptors of erythropoiesis. MCHC, MCV, RBC shared the QTL on the SSC5, MCH and MCHC shared the QTLs on the SSC5 and SSC14 while HCT and HGB shared the QTL on the SSC18. Genes highly expressed in bone marrow are annotated in most of these QTL regions, suggesting that several genetic factors affecting haematopoiesis could determine the observed effects on erythrocyte traits, as also reported in humans and rodents (e.g.^92^).

Relationships among erythrocyte traits were also evidenced from the analysis of the correlation network which identified two phenotypic modules: [RBC–HGB–HCT] and [MCH–MCHC–MCV–RDW]. Results based on these multi-trait groups confirmed, in most cases, single-trait analyses, also strengthening the observed associations. In a few cases, they highlighted additional QTL regions that were just below the significance threshold in the single-marker analyses. This is also obtained for the other multi-trait modules.

Leukocytes are fundamental players of the primary defence mechanisms against pathogen agents and their counts (in the different components) are used as clinical marker of inflammation status. In humans, high WBC count has been associated with cancer mortality and all-cause mortality and several common diseases (e.g.^93,94^). Eosinophil count and LDL-Cholesterol levels showed the largest number of QTLs for a single haematological and clinical-biochemical trait (i.e. five) in the investigated Italian Large White pig population. However, the most significant QTL was reported for basophil count on SSC14. It is worth to mention that this region might actually harbour two QTLs as two close peaks are evident from the Manhattan plot of the single-marker single-trait analysis (even if we could not formally separate them). The same region was reported to harbour a QTL for lymphocyte count in German Landrace pigs^28^. Genetic heterogeneity in this chromosome regions could act at the haematopoiesis level affecting stem cells which might lead to the development of these two classes of leukocytes in the two pig breeds: basophil in Italian Large White and lymphocytes in the German Landrace breed.

For some lipid and enzyme measures, mapped QTLs were able to directly pinpoint candidate genes based on their functions related to the expected effect on the analysed phenotype or that could contribute to explain the biological mechanisms underlying their genetic variability. For example, QTLs reported for cholesterol traits (with estimated SNP heritability of about 0.35–0.38) highlighted several genes involved in the metabolism, homeostasis, transport and regulation of its forms (i.e. *ADCY8*, *APOB*, *ATG5*, *CDKAL1*, *PCSK5*, *PRL* and *SOX6*) as deduced from literature information already available in humans and mice. In particular, variability in the porcine *APOB* gene has been already associated with the level of blood cholesterol in four months-old pigs^95,96^. These studies are considered among the first examples that demonstrated the usefulness of the pig as animal model for cardiovascular diseases, as variability in this gene is associated with atherosclerosis risk. In pigs, other works already identified QTL for Total-cholesterol, LDL-cholesterol, LDL/HDL-cholesterol ratio and TC in the *APOB* region^33,96^. Age-specific associations were reported in Duroc pigs, which showed significant results only in fattening animals (190 days) and not in the post-weaning phase (45 days^32^). The study in Italian Large White pigs was based on just one time point (i.e. slaughtering age of the animals at 270 days), that might confirm an effect on adult pigs of this region for cholesterol measures. However, no association with TC was reported on this SSC3 region in the Italian Large White population. Another interesting aspect that might have age-related implications, is that in the adult Italian Large White pigs, no QTLs for HDL-cholesterol were observed. This is in contrast to what was reported in adult Duroc pigs which showed several QTLs for this cholesterol fraction^32,90^.

Most of the genes located in QTL regions for cholesterol traits in the Italian Large White breed (all except *APOB*) were not identified in corresponding genome wide association studies for the same traits in human cohorts. However, they were associated to fat deposition/obesity traits, cardiovascular disorders and other diseases in several human studies^97^, (see https://www.ebi.ac.uk/gwas/home). The genome-wide association study that was carried out in this heavy pig population captured the effect of genetic variability on cholesterol parameters that could be explained by a detailed analysis of the literature. Thus, across species inference might be important to better inform the functional relevance of genes involved in several biological mechanisms^98^. Gene enrichment analysis confirmed their role in biological processes and molecular mechanisms involving cholesterol.

QTLs for several other traits analysed in this study identified candidate genes that might be directly involved in explaining their genetic variability. For example, a QTL for alanine aminotransferase activity (the enzyme also known as glutamate-pyruvate transaminase or GPT) identified a SSC4 region in which the gene encoding for this enzyme is annotated. Thus far, only another study analysed this serum parameter in a genome scan, which was based on an F2 intercross between Landrace and Korean pigs, showing QTLs in different chromosomes (i.e. SSC1, SSC5 and SSC7^6^). A QTL for the activity of another enzyme [aspartate aminotransferase (AST), also known as aspartate transaminase or serum glutamic oxaloacetic transaminase (sGOT or GOT)] was on a SSC14 region in which the *GOT1* gene (that encodes for the measured enzyme) in annotated. Reiner *et al*.^99^. reported a suggestive AST QTL on SSC14 in a Pietrain/Meishan F_2_ family infected by the common protozoan parasite *Sarcocystis miescheriana*. This QTL became significant after the acute burden of the parasite infection^100^. Subsequent sequence characterization of the *GOT1* gene reported a SNP in the 5′ flanking region that resulted associated to AST even in non-challenged and healthy pigs, further supporting the effect of this region in affecting the activity of this enzyme^96^. Association analysis with the [AST-CK] trait group in the Italian Large White pigs increased largely the significant results observed for the single-trait analysis (based on AST). This result confirmed the presence of genetic variability in this SSC14 region that may play an important role on response to stressing or tissue damaging conditions, according to the diagnostic and predictive potential of these two blood biomarkers^81,82^.

## Conclusions

This study provided new insights into the genetic factors affecting haematological and clinical-biochemical traits in pigs. These traits can be considered intermediate or  internal phenotypes. They are simpler than production or external traits and could be useful to dissect the complex genetic architecture of disease resistance and resilience of the animals to stressing and adverse environmental conditions. Combining different approaches (single- marker with single-trait and multi-trait analyses and Bayesian multi-marker method), this study identified QTL regions for 29 out of 30 analysed blood biomarkers which highlighted promising candidate genes, some of which encode for the analysed enzymes or are directly involved in the biological mechanisms that may explain the variability of the measured parameters. The obtained results can contribute to explore new avenues to overcome the limited genetic progress for disease resistance and related traits that selection programs are currently experiencing due to the difficulties in defining measurable phenotypes for these traits linked to genetic variability in commercial pig populations.

## Supplementary information

Supplementary information: Tables S1-S5 and Figures S1-S4.

## Data Availability

The datasets used and/or analysed during the current study are available from the corresponding author on reasonable request.

## References

[1] Fan B (2011). Genome-wide association study identifies Loci for body composition and structural soundness traits in pigs. PLoS One.

[2] Sanchez M-P (2014). A genome-wide association study of production traits in a commercial population of Large White pigs: evidence of haplotypes affecting meat quality. Genet sel evol.

[3] Fontanesi L, Schiavo G, Galimberti G, Calò DG, Russo V (2014). A genomewide association study for average daily gain in Italian Large White pigs. J Anim Sci.

[4] Fontanesi L (2017). Genome-wide association study for ham weight loss at first salting in Italian Large White pigs: towards the genetic dissection of a key trait for dry-cured ham production. Anim Genet.

[5] Reiner G, Clemens N, Lohner E, Willems H (2010). SNPs in the porcine GOT1 gene improve a QTL for serum aspartate aminotransferase activity on SSC14. Anim Genet.

[6] Yoo C-K (2012). QTL analysis of clinical-chemical traits in an F intercross between Landrace and Korean native pigs. Physiol Genomics.

[7] Bovo S (2016). Genome-wide association study for the level of serum electrolytes in Italian Large White pigs. Anim Genet.

[8] Fontanesi L (2016). Metabolomics and livestock genomics: Insights into a phenotyping frontier and its applications in animal breeding. Anim Front.

[9] Grindem Carol B. (2011). Schalm's Veterinary Hematology, 6th edition. Editors: Douglas J. Weiss, K. Jane Wardrop. Veterinary Clinical Pathology.

[10] Colditz IG (2002). Effects of the immune system on metabolism: implications for production and disease resistance in livestock. Livest Prod Sci.

[11] Bishop SC, Woolliams JA (2014). Genomics and disease resistance studies in livestock. Livest Sci.

[12] Kristjansson RP (2016). Common and rare variants associating with serum levels of creatine kinase and lactate dehydrogenase. Nat Commun.

[13] Soranzo N (2009). A genome-wide meta-analysis identifies 22 loci associated with eight hematological parameters in the HaemGen consortium. Nat Genet.

[14] Nalls MA (2011). Multiple loci are associated with white blood cell phenotypes. PLoS Genet.

[15] Crosslin DR (2012). Genetic variants associated with the white blood cell count in 13,923 subjects in the eMERGE Network. Hum Genet.

[16] Do R (2013). Common variants associated with plasma triglycerides and risk for coronary artery disease. Nat Genet.

[17] Edfors-Lilja I (1998). Mapping quantitative trait loci for immune capacity in the pig. J Immunol.

[18] Reiner G (2007). Quantitative trait loci for red blood cell traits in swine. Anim Genet.

[19] Reiner G (2008). Quantitative trait loci for white blood cell numbers in swine. Anim Genet.

[20] Zou Z (2008). Quantitative trait loci for porcine baseline erythroid traits at three growth ages in a White Duroc × Erhualian F(2) resource population. Mamm Genome.

[21] Yang S (2009). Quantitative trait loci for porcine white blood cells and platelet-related traits in a White Duroc × Erhualian F resource population. Anim Genet.

[22] Gong Y-F (2010). Detection of quantitative trait loci affecting haematological traits in swine via genome scanning. BMC Genet.

[23] Cho IC (2011). QTL analysis of white blood cell, platelet and red blood cell-related traits in an F2 intercross between Landrace and Korean native pigs. Anim Genet.

[24] Luo W (2012). Genome-wide association study of porcine hematological parameters in a Large White × Minzhu F2 resource population. Int J Biol Sci.

[25] Zhang Z (2013). Genome-wide association study reveals constant and specific loci for hematological traits at three time stages in a White Duroc × Erhualian F2 resource population. PLoS One.

[26] Jung EJ (2014). Genome-wide association study identifies quantitative trait loci affecting hematological traits in an F2 intercross between Landrace and Korean native pigs. Anim Genet.

[27] Zhang F (2014). Genome-wide association studies for hematological traits in Chinese Sutai pigs. BMC Genet.

[28] Ponsuksili S, Reyer H, Trakooljul N, Murani E, Wimmers K (2016). Single- and Bayesian Multi-Marker Genome-Wide Association for Haematological Parameters in Pigs. PLoS One.

[29] Schmid M, Bennewitz J (2017). Invited review: Genome-wide association analysis for quantitative traits in livestock – a selective review of statistical models and experimental designs. Arch Anim Breed.

[30] Sahana G, Guldbrandtsen B, Janss L, Lund MS (2010). Comparison of association mapping methods in a complex pedigreed population. Genet Epidemiol.

[31] Galesloot TE, Steen K, Van Kiemeney LALM, Janss LL, Vermeulen SH (2014). A Comparison of Multivariate Genome-Wide Association Methods. PLoS One.

[32] Manunza A (2014). A genome-wide association analysis for porcine serum lipid traits reveals the existence of age-specific genetic determinants. BMC Genomics.

[33] Yang H (2015). Genome-Wide Association Analysis for Blood Lipid Traits Measured in Three Pig Populations Reveals a Substantial Level of Genetic Heterogeneity. PLoS One.

[34] Uddin MJ (2011). Mapping quantitative trait loci for innate immune response in the pig. Int J Immunogenet.

[35] Fontanesi L (2012). Identification and association analysis of several hundred single nucleotide polymorphisms within candidate genes for back fat thickness in Italian Large White pigs using a selective genotyping approach. J Anim Sci.

[36] Purcell S (2007). PLINK: a tool set for whole-genome association and population-based linkage analyses. Am J Hum Genet.

[37] Fontanesi L (2012). A genome wide association study for backfat thickness in Italian Large White pigs highlights new regions affecting fat deposition including neuronal genes. BMC Genomics.

[38] Box GEP, Cox DR (1964). An Analysis of Transformations. J R Stat Soc Ser B Method.

[39] R Core Team. R: A Language and Environment for Statistical Computing. (R Foundation for Statistical Computing 2014).

[40] Shannon P (2003). Cytoscape: a software environment for integrated models of biomolecular interaction networks. Genome Res.

[41] Zhou X, Stephens M (2012). Genome-wide efficient mixed-model analysis for association studies. Nat Genet.

[42] Li M-X, Yeung JMY, Cherny SS, Sham PC (2012). Evaluating the effective numbers of independent tests and significant p-value thresholds in commercial genotyping arrays and public imputation reference datasets. Hum Genet.

[43] Duggal P, Gillanders EM, Holmes TN, Bailey-Wilson JE (2008). Establishing an adjusted p-value threshold to control the family-wide type 1 error in genome wide association studies. BMC Genomics.

[44] Teyssèdre S (2012). Genome-wide association studies for osteochondrosis in French Trotter horses. J Anim Sci.

[45] Allais S (2014). Fine mapping of quantitative trait loci underlying sensory meat quality traits in three French beef cattle breeds. J Anim Sci.

[46] Stratz P, Wellmann R, Preuss S, Wimmers K, Bennewitz J (2014). Genome-wide association analysis for growth, muscularity and meat quality in Piétrain pigs. Anim Genet.

[47] Bertolini F (2018). Genome-wide association studies for seven production traits highlight genomic regions useful to dissect dry-cured ham quality and production traits in Duroc heavy pigs. Animal.

[48] Barrett JC, Fry B, Maller J, Daly MJ (2005). Haploview: analysis and visualization of LD and haplotype maps. Bioinformatics.

[49] Shim H (2015). A multivariate genome-wide association analysis of 10 LDL subfractions, and their response to statin treatment, in 1868 Caucasians. PLoS One.

[50] Browning BL, Browning SR (2016). Genotype Imputation with Millions of Reference Samples. Am J Hum Genet.

[51] Fernando, R. L & Garrick, D. J. GenSel—User manual for a portfolio of genomic selection related analyses. Animal Breeding and Genetics, Iowa State University, Ames (2008).

[52] Sollero BP, Junqueira VS, Gomes CCG, Caetano AR, Cardoso FF (2017). Tag SNP selection for prediction of tick resistance in Brazilian Braford and Hereford cattle breeds using Bayesian methods. Genet Sel Evol.

[53] Reyer H, Hawken R, Murani E, Ponsuksili S, Wimmers K (2015). The genetics of feed conversion efficiency traits in a commercial broiler line. Sci Rep.

[54] Shen M (2017). Genetic Architecture and Candidate Genes Identified for Follicle Number in Chicken. Sci Rep.

[55] Reyer H, Varley PF, Murani E, Ponsuksili S, Wimmers K (2017). Genetics of body fat mass and related traits in a pig population selected for leanness. Sci Rep.

[56] Bovo S, Di Lena P, Martelli PL, Fariselli P, Casadio R (2016). NET-GE: a web-server for NETwork-based human gene enrichment. Bioinformatics.

[57] Hu Z-L, Park CA, Reecy JM (2016). Developmental progress and current status of the Animal QTLdb. Nucleic Acids Res.

[58] Chami N (2016). Exome Genotyping Identifies Pleiotropic Variants Associated with Red Blood Cell Traits. Am J Hum Genet.

[59] Almusafri F (2017). Clinical and molecular characterization of 6 children with glutamate-cysteine ligase deficiency causing hemolytic anemia. Blood Cells Mol Dis.

[60] Kulkeaw K (2018). Purification of zebrafish erythrocytes as a means of identifying a novel regulator of haematopoiesis. Br J Haematol.

[61] Astle WJ (2016). The Allelic Landscape of Human Blood Cell Trait Variation and Links to Common Complex Disease. Cell.

[62] Kanai M (2018). Genetic analysis of quantitative traits in the Japanese population links cell types to complex human diseases. Nat Genet.

[63] Miyata Y (2010). Cyclin C regulates human hematopoietic stem/progenitor cell quiescence. Stem Cells.

[64] Miao T (2017). Egr2 and 3 control adaptive immune responses by temporally uncoupling expansion from T cell differentiation. J Exp Med.

[65] Wang LD (2015). The role of Lin28b in myeloid and mast cell differentiation and mast cell malignancy. Leukemia.

[66] Tajuddin SM (2016). Large-Scale Exome-wide Association Analysis Identifies Loci for White Blood Cell Traits and Pleiotropy with Immune-Mediated Diseases. Am J Hum Genet.

[67] Li J (2015). Copy Number Variations in CTNNA3 and RBFOX1 Associate with Pediatric Food Allergy. J Immunol.

[68] Whitfield AJ, Barrett PHR, Van Bockxmeer FM, Burnett JR (2004). Lipid disorders and mutations in the APOB gene. Clin Chem.

[69] Ouimet M (2011). Autophagy regulates cholesterol efflux from macrophage foam cells via lysosomal acid lipase. Cell Metab.

[70] Razani B (2012). Autophagy links inflammasomes to atherosclerotic progression. Cell Metab.

[71] Iatan I (2009). Genetic variation at the proprotein convertase subtilisin/kexin type 5 gene modulates highdensity lipoprotein cholesterol levels. Circ Cardiovasc Genet.

[72] Silver M (2013). Pathways-driven sparse regression identifies pathways and genes associated with highdensity lipoprotein cholesterol in two Asian cohorts. PLoS Genet.

[73] Cheon EJ (2018). Novel association between CDKAL1 and cholesterol efflux capacity: Replication after GWAS-based discovery. Atherosclerosis.

[74] Cincotta AH, Meier AH (1989). Reductions of body fat stores and total plasma cholesterol and triglyceride concentrations in several species by bromocriptine treatment. Life Sci.

[75] Leow SC (2016). The transcription factor SOX6 contributes to the developmental origins of obesity by promoting adipogenesis. Development.

[76] Palmieri F (2013). The mitochondrial transporter family SLC25: identification, properties and physiopathology. Mol Aspects Med.

[77] Lin H-P (2016). Destabilization of Fatty Acid Synthase by Acetylation Inhibits De Novo Lipogenesis and Tumor Cell Growth. Cancer Res.

[78] Heard-Costa NL (2009). NRXN3 is a novel locus for waist circumference: a genome-wide association study from the CHARGE Consortium. PLoS Genet.

[79] Chen H-M, Zheng C-X, Gao Q, Ge Y-C, Liu Z-H (2012). Heart-type fatty acid binding protein is associated with proteinuria in obesity. PLoS One.

[80] Dumont V (2017). PACSIN2 accelerates nephrin trafficking and is up-regulated in diabetic kidney disease. FASEB J.

[81] Jackson, P. G. G. & Cockcroft, P. D. *Handbook of Pig**Medicine*. (Elsevier Health Sciences, 2007).

[82] Tvarijonaviciute A (2017). Measurement of Creatine kinase and Aspartate aminotransferase in saliva of dogs: a pilot study. BMC Vet Res.

[83] Yuan X (2008). Population-based genome-wide association studies reveal six loci influencing plasma levels of liver enzymes. Am J Hum Genet.

[84] Bovo S, Lena PD, Martelli PL, Fariselli P, Casadio R (2017). From Protein Variations to Biological Processes and Pathways with NET-GE. Genomics Comput Biol.

[85] Myers MJ, Smith ER, Turfle PG (2017). Biomarkers in Veterinary Medicine. Annu Rev Anim Biosci.

[86] Royer E, Barbé F, Guillou D, Rousselière Y, Chevaux E (2016). Development of an oxidative stress model in weaned pigs highlighting plasma biomarkers’ specificity to stress inducers. J Anim Sci.

[87] Bishop SC (2012). A consideration of resistance and tolerance for ruminant nematode infections. Front Genet.

[88] Lu X (2011). Mapping quantitative trait loci for T lymphocyte subpopulations in peripheral blood in swine. BMC Genet.

[89] Wang JY (2013). Genome-wide association studies for hematological traits in swine. Anim Genet.

[90] Gallardo D (2008). Mapping of quantitative trait loci for cholesterol, LDL, HDL, and triglyceride serum concentrations in pigs. Physiol Genomics.

[91] Chen C (2013). Genetic dissection of blood lipid traits by integrating genome-wide association study and gene expression profiling in a porcine model. BMC Genomics.

[92] Orkin SH (2000). Diversification of haematopoietic stem cells to specific lineages. Nat Rev Genet.

[93] Ruggiero C (2007). White blood cell count and mortality in the Baltimore Longitudinal Study of Aging. J Am Coll Cardiol.

[94] Danesh J, Collins R, Appleby P, Peto R (1998). Association of fibrinogen, C-reactive protein, albumin, or leukocyte count with coronary heart disease: meta-analyses of prospective studies. JAMA.

[95] Rapacz J, Hasler-Rapacz J, Taylor KM, Checovich WJ, Attie AD (1986). Lipoprotein mutations in pigs are associated with elevated plasma cholesterol and atherosclerosis. Science.

[96] Prescott MF, McBride CH, Hasler-Rapacz J, Von Linden J, Rapacz J (1991). Development of complex atherosclerotic lesions in pigs with inherited hyper-LDL cholesterolemia bearing mutant alleles for apolipoprotein B. Am J Pathol.

[97] MacArthur J (2017). The new NHGRI-EBI Catalog of published genome-wide association studies (GWAS Catalog). Nucleic Acids Res.

[98] Ashburner M (2000). Gene ontology: tool for the unification of biology. The Gene Ontology Consortium. Nat Genet.

[99] Reiner G (2007). Mapping of quantitative trait loci affecting resistance/susceptibility to Sarcocystis miescheriana in swine. Genomics.

[100] Reiner G (2007). Genetic resistance to Sarcocystis miescheriana in pigs following experimental infection. Vet Parasitol.

