# Supplementary material for: Genome-wide association studies for 30 haematological and blood clinical-biochemical traits in Large White pigs reveal genomic regions affecting intermediate phenotypes
Source: Sci Rep. 2019 May 7;9:7003. doi: 10.1038/s41598-019-43297-1 (PMC6504931; doi:10.1038/s41598-019-43297-1)
Supplement: Supplementary file 1 — Supplementary information: Tables S1-S5 and Figures S1-S4. [file 41598_2019_43297_MOESM1_ESM.pdf]

Supplementary information

Genome-wide association studies for 30 haematological and blood clinical-biochemical traits in Large White pigs reveal genomic regions affecting intermediate phenotypes

Samuele Bovo, Gianluca Mazzoni, Francesca Bertolini, Giuseppina Schiavo, Giuliano Galimberti, Maurizio Gallo, Stefania Dall'Olio, Luca Fontanesi

Supplementary Table S1. Descriptive statistics of the 30 blood traits analyzed in the Italian Large White pig population.

Parameters (units)	Abbreviation	Formula	Mean	Standard deviation	Genomic heritability (Standard Error)
**Haematological parameters**					
Erythrocyte traits					
Red blood cell count (cells/μL)	RBC	-	8,363,333	709,858	0.357 (0.066)
Hemoglobin (g/dL)	HGB	-	14.3	1.4	0.361 (0.064)
Hematocrit (%)	HCT	-	49.0	4.3	0.274 (0.061)
Mean corpuscular volume (fL)	MCV	$\frac{HCT(\%)\times 10}{RBC\ (\times 10^{12}/_{L})}$	58.7	4	0.393 (0.066)
Mean corpuscular hemoglobin (pg)	MCH	$\frac{HGB(^{g}/_{dL})\times 10}{RBC\ (\times 10^{12}/_{L})}$	17.1	1.5	0.399 (0.064)
Mean corpuscular hemoglobin concentration (g/dL)	MCHC	$\frac{HGB(^{g}/_{dL})\times 100}{HCT\ (\%)}$	29.1	1.2	0.244 (0.056)
Red cell distribution width (%)	RDW	-	16.9	2.1	0.387 (0.064)
Leukocyte traits					
White blood cell count (cells/μL)	WBC	-	23,299	5,728	0.311 (0.071)
Lymphocyte count (cells/μL)	LYMPHO	-	8,230	2,019	0.313 (0.061)
Neutrophil count (cells/μL)	NEUTRO	-	14,008	5,646	0.244 (0.071)
Eosinophil count (cells/μL)	EOSINO	-	149	97	0.137 (0.059)
Basophil count (cells/μL)	BASO	-	139	54	0.193 (0.055)
Monocyte count (cells/μL)	MONO	-	577	259	0.147 (0.044)
Platelet traits					
Platelet count (cells/μL)	PLT	-	340,632	99,473	0.307 (0.059)
Mean platelet volume (fL)	MPV	-	9.6	1.4	0.367 (0.063)
**Clinical-biochemical parameters**					
Lipid related traits					
Total cholesterol (mg/dL)	T-Chol	-	98	14	0.380 (0.060)
High-density lipoprotein cholesterol	HDL-Chol	-	38.9	9.5	0.354 (0.061)

Low-density lipoprotein cholesterol	LDL-Chol	-	55	10	0.381 (0.060)
Triglycerides (mg/dL)	TG	-	46	16	0.288 (0.067)
Non-esterified fatty acids (mmol/L)	NEFA	-	0.18	0.11	0.128 (0.056)
Metabolism and protein related traits					
Glucose (mg/dL)	Glu	-	121	31	0.087 (0.044)
Urea (mg/dL)	UA	-	31.93	6.78	0.367 (0.060)
Total bilirubin (mg/dL)	T-Bil	-	0.14	0.04	0.004 (0.028)
Total proteins (g/dL)	T-Prot	-	7.73	0.53	0.303 (0.067)
Albumin (g/dL)	Alb	-	4.04	0.35	0.248 (0.056)
Albumin/Globulines ratio	Alb-Glob-ratio	-	1.12	0.22	0.294 (0.068)
Enzyme traits					
Alkaline phosphatase (u/L)	ALP	-	269	221	0.219 (0.062)
Creatine kinase (u/dL)	CK	-	8,149	10,699	0.338 (0.062)
Alanine aminotransferase (u/dL)	ALT	-	51	11	0.433 (0.062)
Aspartate aminotransferase (u/dL)	AST	-	90	71	0.441 (0.061)

Supplementary Table S2. Whole set of single nucleotide polymorphisms identified in the single marker genome wide association studies for the 30 blood parameters.

Trait[1]	SSC[2]	Marker	Position (bp)[3]	m/M alleles[4]	MAF[5]	*p*-value	PVE(%)[6]
Alb	6	H3GA0052531	88193827	A/G	0.4603	4.37E-05	3.85
Alb-Glob-ratio	5	H3GA0015245	5996798	A/G	0.3875	1.59E-05	4.29
ALT	4	H3GA0053919	586320	G/A	0.1376	4.59E-05	3.83
ALT	4	ASGA0023643	1007973	A/G	0.1732	1.78E-05	4.23
ALT	4	M1GA0007042	1035397	A/G	0.1631	2.67E-05	4.06
ALT	4	ALGA0029783	1175147	G/A	0.1305	4.99E-06	4.77
ALT	18	ALGA0098672	48131574	A/G	0.2307	1.11E-05	4.43
ALT	NW_018084979.1	ASGA0037264	2390114	A/G	0.4520	2.38E-05	4.11
ALT	NW_018084979.1	ASGA0037274	2493881	A/G	0.4609	4.70E-05	3.87
ALT	NW_018084979.1	M1GA0026306	2683385	G/A	0.3772	2.64E-05	4.07
ALT	NW_018084979.1	ALGA0110485	2700480	A/G	0.3515	2.06E-05	4.17
ALT	NW_018084979.1	H3GA0055095	2704835	G/A	0.3854	6.77E-06	4.65
ALT	NW_018084979.1	ASGA0100304	2731684	G/A	0.3582	1.97E-05	4.19
ALT	NW_018084979.1	ASGA0096862	2817806	A/G	0.3790	1.70E-05	4.25
ALT	NW_018084979.1	H3GA0023887	3163232	G/A	0.3725	3.88E-06	4.88
ALT	NW_018084979.1	M1GA0011476	3221013	G/A	0.3805	1.83E-05	4.25
ALT	NW_018084979.1	ASGA0037291	3252230	G/A	0.3511	4.21E-05	3.87
ALT	NW_018084979.1	ASGA0037279	3287126	A/G	0.3511	4.21E-05	3.87
ALT	NW_018084979.1	ASGA0037286	3301399	A/C	0.4790	7.93E-06	4.76
AST	14	ASGA0066070	1.1E+08	A/G	0.3238	3.07E-05	4.00
AST	14	H3GA0042029	1.1E+08	A/G	0.3238	3.07E-05	4.00
AST	14	ALGA0081018	1.1E+08	G/A	0.3233	2.92E-05	4.02
AST	14	ALGA0081020	1.1E+08	G/A	0.3238	3.07E-05	4.00
AST	14	INRA0046629	1.1E+08	A/G	0.3203	1.15E-05	4.42
AST	14	ALGA0081025	1.11E+08	A/C	0.3996	2.04E-05	4.23
BASO	14	ALGA0078342	63301421	A/C	0.4247	2.57E-05	4.08
BASO	14	INRA0044634	66411511	A/G	0.1299	4.72E-06	4.79
BASO	14	ALGA0078500	66463802	G/A	0.1418	1.47E-06	5.29

BASO	14	ASGA0064195	66511894	C/A	0.1295	4.14E-06	4.86
BASO	14	ASGA0064196	66543901	G/A	0.1280	2.70E-06	5.07
BASO	14	MARC0069902	66601592	A/G	0.1412	2.30E-06	5.10
BASO	14	DIAS0002104	66650724	G/A	0.1412	2.30E-06	5.10
BASO	14	INRA0044651	66713900	A/G	0.1412	2.30E-06	5.10
BASO	14	INRA0044654	66739480	C/A	0.1407	2.03E-06	5.15
BASO	14	ASGA0064202	66774513	A/G	0.1412	2.30E-06	5.10
BASO	14	ASGA0064204	66813618	G/A	0.1412	2.30E-06	5.10
BASO	14	ALGA0078520	66952063	A/G	0.1453	2.83E-06	5.01
BASO	14	ALGA0078523	67039033	G/A	0.1412	2.30E-06	5.10
BASO	14	DRGA0014010	67093823	G/A	0.1412	2.30E-06	5.10
BASO	14	DRGA0014012	67147013	G/A	0.1412	2.30E-06	5.10
BASO	14	MARC0013889	67164506	G/A	0.1412	2.30E-06	5.10
BASO	14	ASGA0064211	67179643	G/A	0.3529	5.42E-07	5.71
BASO	14	MARC0095482	67221608	A/G	0.1335	9.15E-06	4.51
BASO	14	H3GA0040774	67243311	G/A	0.3571	9.16E-07	5.48
BASO	14	ASGA0064218	67269867	G/A	0.3612	1.11E-06	5.40
BASO	14	ALGA0078532	67334411	G/A	0.3612	1.11E-06	5.40
BASO	14	ALGA0078534	67342053	G/A	0.3612	1.11E-06	5.40
BASO	14	ALGA0078541	67409552	G/A	0.3499	2.75E-06	5.02
BASO	14	DRGA0014018	67429030	A/G	0.1376	1.12E-05	4.43
BASO	14	ALGA0078548	67443330	G/A	0.1453	2.83E-06	5.01
BASO	14	H3GA0052373	67551686	A/G	0.3600	9.49E-07	5.47
BASO	14	INRA0044677	67556019	A/G	0.3600	9.49E-07	5.47
BASO	14	ASGA0064234	67864215	G/A	0.1453	2.83E-06	5.01
BASO	14	ALGA0078557	67944945	G/A	0.1435	2.61E-06	5.04
BASO	14	MARC0109364	67985525	A/G	0.1453	2.83E-06	5.01
BASO	14	DRGA0014039	68444298	C/A	0.1443	4.12E-06	4.86
BASO	14	DRGA0014049	68924011	G/A	0.1425	2.27E-06	5.15
BASO	14	CASI0008086	68966102	A/G	0.1530	1.73E-06	5.22
BASO	14	DRGA0014060	69076616	A/G	0.1554	2.24E-06	5.11
BASO	14	DRGA0014062	69112868	A/G	0.1554	2.24E-06	5.11
BASO	14	DRGA0014064	69217969	A/G	0.1483	9.62E-07	5.46
BASO	14	ALGA0078618	69290179	G/A	0.1412	2.30E-06	5.10
BASO	14	DRGA0014066	69323867	A/G	0.1483	9.62E-07	5.46

BASO	14	ALGA0078633	69589926	C/A	0.1613	4.69E-05	3.82
BASO	14	H3GA0040804	69721367	G/A	0.1717	4.76E-05	3.91
BASO	14	ASGA0064288	69743473	A/G	0.1601	4.57E-05	3.83
BASO	14	ALGA0078642	69814326	A/C	0.1489	7.87E-07	5.55
BASO	14	MARC0092344	69865089	A/G	0.1489	7.87E-07	5.55
BASO	14	ALGA0078645	69888948	G/A	0.1489	7.87E-07	5.55
BASO	14	MARC0006045	69943312	G/A	0.1340	7.74E-06	4.59
BASO	14	MARC0007983	69965414	A/G	0.1388	1.63E-05	4.27
BASO	14	ASGA0064298	70036197	A/G	0.1536	1.68E-06	5.23
BASO	14	ASGA0064299	70068946	A/G	0.1661	8.52E-06	4.54
BASO	14	ALGA0078660	70099607	G/A	0.1661	8.52E-06	4.54
BASO	14	ALGA0078667	70216594	G/A	0.1524	7.01E-07	5.60
BASO	14	ASGA0064303	70250940	G/A	0.1524	7.01E-07	5.60
BASO	14	CASI0008356	70268234	A/G	0.1607	3.81E-07	5.85
BASO	14	DRGA0014086	70282756	G/A	0.1655	7.99E-07	5.54
BASO	14	DRGA0014089	70316991	G/A	0.1655	7.99E-07	5.54
BASO	14	MARC0003944	70334131	A/G	0.1530	1.40E-06	5.31
BASO	14	ALGA0078676	70360906	A/G	0.1655	7.99E-07	5.54
BASO	14	ASGA0099631	70394534	C/A	0.1453	1.76E-06	5.21
BASO	14	ASGA0064307	70444514	G/A	0.1524	7.01E-07	5.60
BASO	14	ALGA0078688	70502037	G/A	0.1524	7.01E-07	5.60
BASO	14	ALGA0078691	70524003	G/A	0.1329	3.21E-06	4.96
BASO	14	ALGA0078692	70535908	A/G	0.1329	3.21E-06	4.96
BASO	14	DRGA0014093	70607575	G/A	0.1317	3.70E-06	4.90
BASO	14	MARC0104070	70974541	A/G	0.1228	7.12E-08	6.55
BASO	14	H3GA0040825	71051046	G/A	0.1851	2.22E-06	5.11
BASO	14	MARC0019955	71087715	A/C	0.1228	7.12E-08	6.55
BASO	14	INRA0044866	71116678	A/G	0.1358	2.31E-07	6.06
BASO	14	CASI0009762	71171960	G/A	0.1358	2.31E-07	6.06
BASO	14	ALGA0113073	71377302	A/G	0.1346	1.70E-07	6.19
BASO	14	MARC0084716	71463045	A/G	0.1346	1.70E-07	6.19
BASO	14	MARC0046932	71515970	A/C	0.1346	1.70E-07	6.19
BASO	14	MARC0064193	71530810	G/A	0.1346	1.70E-07	6.19
BASO	14	ALGA0079514	71575626	G/A	0.1318	1.25E-07	6.33
BASO	14	MARC0065976	71578222	G/A	0.1323	1.45E-07	6.26

BASO	14	ALGA0079525	71604272	G/A	0.1103	6.77E-08	6.57
BASO	14	ASGA0064974	71926501	A/G	0.1346	1.70E-07	6.19
BASO	14	ALGA0079529	71958965	A/G	0.1139	6.53E-09	7.54
BASO	14	ALGA0079530	71996220	A/G	0.1174	1.84E-07	6.16
BASO	14	DIAS0001667	72025065	G/A	0.1281	2.19E-08	7.04
BASO	14	ASGA0064979	72070115	A/G	0.1281	2.19E-08	7.04
BASO	14	ALGA0079538	72086976	G/A	0.1240	3.16E-08	6.89
BASO	14	MARC0030485	72230609	G/A	0.1002	7.10E-08	6.55
BASO	14	INRA0045518	72254375	A/G	0.1026	1.41E-07	6.27
BASO	14	H3GA0041322	72418293	G/A	0.1329	6.54E-08	6.59
BASO	14	ALGA0079580	72454977	G/A	0.1281	2.19E-08	7.04
BASO	14	ALGA0079583	72478930	G/A	0.1435	2.15E-06	5.13
BASO	14	DIAS0001570	72544071	G/A	0.1281	2.19E-08	7.04
BASO	14	ALGA0079602	72571424	G/A	0.1115	7.66E-07	5.62
BASO	14	MARC0057405	72691300	A/G	0.1269	1.86E-08	7.11
BASO	14	MARC0090899	72704456	A/G	0.1133	3.07E-09	7.85
BASO	14	ALGA0078726	72824472	G/A	0.1305	2.44E-08	7.00
BASO	14	H3GA0040831	72876839	A/G	0.1234	3.17E-07	5.93
BASO	14	M1GA0018799	72901799	A/G	0.1241	1.40E-07	6.28
BASO	14	ASGA0064326	72965155	G/A	0.1103	2.74E-08	6.95
BASO	14	ALGA0078729	73118420	G/A	0.1103	7.89E-07	5.55
BASO	14	ALGA0078757	73396066	G/A	0.1275	5.68E-06	4.72
BASO	14	ALGA0078765	73434714	A/G	0.1097	1.36E-05	4.35
BASO	14	ASGA0064370	73455311	G/A	0.1097	1.36E-05	4.35
BASO	14	ALGA0078783	73476027	A/G	0.1275	5.68E-06	4.72
BASO	14	MARC0033060	91341310	G/A	0.1406	1.97E-05	4.19
BASO	14	ALGA0079922	91363743	A/C	0.1477	5.49E-06	4.73
BASO	14	ALGA0079939	91504896	G/A	0.3599	3.75E-05	3.92
EOSINO	3	H3GA0009277	35094559	C/A	0.2254	3.69E-05	3.92
EOSINO	3	H3GA0010692	1.17E+08	A/C	0.2503	4.28E-05	3.86
EOSINO	7	H3GA0021970	61362991	C/A	0.2805	2.60E-05	4.07
EOSINO	7	INRA0028736	1.18E+08	G/A	0.0990	6.47E-06	4.66
EOSINO	10	H3GA0030197	46331882	G/A	0.1940	2.42E-05	4.10
Glu	7	ALGA0110857	1.01E+08	A/G	0.4081	2.08E-05	4.16
HCT	18	MARC0020251	36745365	G/A	0.3897	3.11E-05	3.99

HCT	18	DRGA0017710	36749906	G/A	0.4015	2.37E-05	4.11
HGB	13	CASI0007727	1.77E+08	G/A	0.0706	2.16E-06	5.12
HGB	18	MARC0020251	36745365	G/A	0.3897	2.65E-05	4.06
HGB	18	DRGA0017710	36749906	G/A	0.4015	2.12E-05	4.16
LDL-Chol	1	ALGA0004272	72306533	G/A	0.1100	5.14E-06	4.80
LDL-Chol	1	ALGA0004279	72428453	A/G	0.1305	4.22E-05	3.87
LDL-Chol	1	ALGA0008284	2.29E+08	A/G	0.4727	6.05E-06	4.69
LDL-Chol	1	MARC0052175	2.34E+08	G/A	0.3665	1.31E-05	4.56
LDL-Chol	3	ASGA0013487	12471350	A/G	0.4649	2.24E-05	4.14
LDL-Chol	3	ASGA0016309	1.17E+08	A/G	0.3090	6.12E-06	4.68
LDL-Chol	3	ASGA0016313	1.17E+08	G/A	0.1449	1.18E-07	6.35
LDL-Chol	3	DIAS0000055	1.17E+08	A/G	0.4732	1.71E-10	9.05
LDL-Chol	14	ALGA0077250	43459397	A/G	0.4822	2.58E-05	4.08
LIMPHO	2	DIAS0001270	3257622	A/G	0.1702	4.10E-05	3.88
MCH	5	MARC0069472	14820993	C/A	0.0765	3.86E-06	4.88
MCH	14	H3GA0038580	6432121	A/G	0.0759	3.45E-06	4.93
MCHC	5	MARC0044114	85947536	G/A	0.2331	9.05E-06	4.52
MCHC	14	ALGA0074674	6409504	G/A	0.3416	4.45E-05	3.84
MCHC	14	H3GA0038597	6641352	G/A	0.3511	3.56E-05	3.94
MCV	5	MARC0069472	14820993	C/A	0.0765	1.63E-05	4.27
MCV	8	DRGA0008367	17423203	G/A	0.3286	2.97E-05	4.01
MONO	15	ALGA0084320	19853411	A/G	0.1453	6.58E-06	4.65
MONO	15	ALGA0084354	20487669	A/C	0.0605	3.20E-05	3.98
MPV	12	ASGA0053310	13483964	A/G	0.2817	3.54E-05	3.94
NEFA	2	ALGA0015164	1.15E+08	G/A	0.3381	2.25E-05	4.13
NEFA	9	ASGA0041146	5738251	G/A	0.1459	2.37E-05	4.11
NEFA	14	MARC0068396	63488234	A/C	0.1097	3.09E-05	4.00
NEFA	14	ALGA0078353	63515463	C/A	0.1097	3.09E-05	4.00
NEFA	14	MARC0033238	63574364	A/G	0.1103	4.23E-05	3.86
NEFA	14	ASGA0064107	63629712	A/G	0.1121	3.64E-05	3.93
NEFA	14	MARC0047133	63934099	A/G	0.0807	1.76E-05	4.24
NEFA	14	ASGA0064132	64162795	A/G	0.0848	1.97E-05	4.19
NEFA	14	DRGA0013970	64191357	A/C	0.0794	8.83E-06	4.56
NEFA	14	ALGA0078392	64221190	A/G	0.1070	4.76E-05	3.82
NEFA	14	ASGA0064154	64593845	G/A	0.0753	1.69E-05	4.25

NEFA	14	ASGA0064161	64856186	C/A	0.1056	4.66E-05	3.82
NEUTRO	4	MARC0052177	77597154	G/A	0.4152	4.27E-05	3.86
PLT	1	ALGA0001781	24412234	A/G	0.4032	1.73E-05	4.30
PLT	1	CASI0009793	24473717	A/G	0.3482	2.76E-05	4.05
PLT	4	MARC0047043	94795752	G/A	0.2550	1.77E-05	4.24
RBC	5	M1GA0007649	15060402	G/A	0.1222	3.33E-05	3.97
RDW	12	DIAS0000242	40010805	A/G	0.2838	3.65E-05	3.93
RDW	16	ASGA0072968	33184787	A/C	0.3108	4.79E-05	3.81
RDW	16	ALGA0090171	33206877	G/A	0.3671	3.69E-06	4.90
RDW	16	ALGA0091410	67367349	A/G	0.0866	1.51E-05	4.30
T-Bil	14	ASGA0067171	1.3E+08	G/A	0.4875	2.61E-05	4.07
T-Bil	17	ALGA0094849	37006714	A/G	0.1996	2.09E-05	4.21
T-Chol	3	DIAS0000055	1.17E+08	A/G	0.4732	8.03E-09	7.48
T-Chol	4	ALGA0022970	9713080	C/A	0.4899	2.11E-05	4.16
T-Chol	5	ASGA0104003	80770124	C/A	0.2357	1.18E-05	4.41
T-Chol	7	MARC0003814	17166311	A/C	0.2023	3.94E-05	3.89
TG	16	ASGA0073326	47077521	A/G	0.4680	3.53E-05	3.94
T-Prot	17	M1GA0022271	52958671	A/G	0.2633	2.62E-05	4.07
UA	5	ALGA0031618	31257070	A/G	0.3037	3.07E-05	4.00
UA	5	ALGA0031630	31476339	C/A	0.3037	3.07E-05	4.00
UA	9	ALGA0051311	10886506	A/G	0.3001	3.33E-05	3.97
WBC	6	DIAS0004496	97994715	A/G	0.0617	4.27E-05	3.86

[1]Full names are reported in Additional File 1: Table S1.

[2]SSC = *Sus scrofa* chromosome; NW_018084979.1 is an unassembled scaffold.

[3]Position (bp): position based on the Sscrofa11.1 reference genome;

[4]m/M Allele = minor/major allele;

[5]MAF = Minor Allele Frequency;

[6]PVE(%): proportion of variance in phenotype explained by the SNP.

Supplementary Table S3. Whole set of single nucleotide polymorphisms identified in the multivariate genome wide association studies for the six medium/high (|*r*| > 0.6) correlated sets of blood parameters.

Groups of traits	SSC[1]	Marker	Position (bp)[2]	m/M allele[3]	MAF[4]	*p*-value[5]	Identified[6]
Alb–Alb-Glob-ratio	7	INRA0025193	38259165	A/G	0.406	2.80E-05	Alb-Glob-ratio
CK–AST	2	ALGA0112586	8371601	G/A	0.483	4.21E-05	**
	2	MARC0016794	8393235	C/A	0.488	2.24E-05	**
	2	MARC0056808	8401366	G/A	0.469	4.55E-05	**
	14	MARC0026910	107584405	G/A	0.457	1.61E-05	**
	14	ALGA0080896	108134979	G/A	0.487	2.77E-05	**
	14	ALGA0080935	108956441	A/G	0.324	2.87E-06	**
	14	ASGA0066004	109011557	A/G	0.302	9.93E-07	**
	14	ALGA0080940	109048435	A/G	0.302	9.93E-07	**
	14	ALGA0080944	109071267	A/G	0.301	6.35E-07	**
	14	ALGA0038327	109305728	A/C	0.335	8.11E-10	**
	14	ASGA0093112	109329518	A/G	0.337	8.82E-10	**
	14	ALGA0080958	109444557	G/A	0.337	7.61E-10	**
	14	ASGA0066039	109497913	G/A	0.447	1.50E-10	**
	14	ASGA0066041	109537669	A/G	0.447	1.50E-10	**
	14	H3GA0042007	109554549	A/G	0.447	1.50E-10	**
	14	ALGA0080984	109576733	A/G	0.448	1.07E-10	**
	14	ASGA0066048	109599604	A/G	0.223	8.67E-06	**
	14	ALGA0080999	109616495	A/G	0.415	3.71E-07	**
	14	CASI0009983	109688469	A/G	0.339	2.08E-09	**
	14	ASGA0066070	109742247	A/G	0.324	1.28E-12	AST
	14	H3GA0042029	109774665	A/G	0.324	1.28E-12	AST
	14	ALGA0081018	109823787	G/A	0.323	4.55E-13	AST
	14	ALGA0081020	109884548	G/A	0.324	1.28E-12	AST
	14	CASI0007888	110150467	A/G	0.473	7.77E-06	**
	14	INRA0046629	110424327	A/G	0.320	1.39E-13	AST
	14	INRA0046630	110536742	G/A	0.082	3.20E-05	**
	14	ALGA0081025	110570159	A/C	0.400	2.54E-05	AST

	14	ASGA0066077	110656834	A/G	0.428	9.53E-11	**
	14	ALGA0081039	110742109	G/A	0.339	2.35E-08	**
	14	ASGA0066087	110784465	C/A	0.333	6.15E-09	**
	14	MARC0043866	110837017	A/G	0.340	1.46E-07	**
	14	H3GA0042042	110891845	A/G	0.332	2.63E-09	**
	14	MARC0015087	111922361	G/A	0.291	3.09E-07	**
MCH–MCHC–MCV–RDW	5	MARC0069472	14820993	C/A	0.077	1.73E-06	MCH, MCV
	7	ASGA0036974	120968439	G/A	0.082	5.50E-07	**
	7	ASGA0037027	121152195	A/G	0.077	1.24E-06	**
	14	ASGA0060510	2891771	A/C	0.464	4.73E-05	MCH
	14	H3GA0038580	6432121	A/G	0.076	3.33E-05	MCH
RBC–HGB–HCT	5	MARC0069472	14820993	C/A	0.077	1.68E-05	**
	13	CASI0007727	177262726	G/A	0.071	3.47E-05	HGB
	14	H3GA0038580	6432121	A/G	0.076	3.29E-05	**
T-Chol–LDL-Chol–HDL-Chol	1	ALGA0004272	72306533	G/A	0.110	3.75E-05	LDL-Chol
	2	ALGA0013564	43603656	C/A	0.406	5.00E-05	**
	3	ASGA0016309	116576491	A/G	0.309	3.65E-05	LDL-Chol
	3	ASGA0016313	116718459	G/A	0.145	5.24E-12	LDL-Chol
	3	DRGA0004246	117086029	C/A	0.098	1.74E-05	**
	3	DIAS0000055	117295071	A/G	0.473	8.55E-12	LDL-Chol, T-Chol
	4	ALGA0022970	9713080	C/A	0.490	4.77E-05	T-Chol, HDL-Chol

[1]Full names are reported in Supplementary Table S1. No significant markers were reported for the [NEUTRO-WBC] cluster.
[2]SSC = *Sus scrofa* chromosome.
[3]Position (bp): position based on the Sscrofa11.1 reference genome.
[4]m/M Allele = minor/major allele.
[5]MAF = Minor Allele Frequency.
[6]The double star symbol (**) indicates SNPs that were not reported to be associated in the single marker single trait approach and in the Bayesian method. Single nucleotide polymorphisms indicated in red were not located in any QTL regions identified with the other two approaches. Single nucleotide polymorphisms indicated in black were already reported to be in QTL regions identified with the other two approaches. When SNPs were already reported to be associated with investigated traits, this information is indicated (full names are reported in Supplementary Table S1). Detailed information of the QTL regions is reported in Additional File 4: Table S4.

Table S4. Full list of QTLs regions (based on tag single nucleotide polymorphisms) associated to the 30 blood parameters.

Class	Trait(s)[1]	SSC[2]	Marker(s)	Position(bp)[3]	QTL progressive number
Haematological trait	PLT	1	MARC0028659-DRGA0000307	24020779-24830003	1
Haematological trait	PLT	1	ALGA0001781	24412234	1
Clinical-biochemical trait	LDL-Chol<br>T-Chol–LDL-Chol–HDL-Chol	1	ALGA0004272	72306533	2
Clinical-biochemical trait	LDL-Chol	1	ALGA0008261-ASGA0005957	228174388-228987611	3
Clinical-biochemical trait	LDL-Chol	1	ALGA0008284	228918979	3
Clinical-biochemical trait	LDL-Chol	1	MARC0052175	233651896	3
Haematological trait	LIMPHO	2	DIAS0001270	3257622	4
Clinical-biochemical trait	CK–AST	2	MARC0016794	8393235	5
Clinical-biochemical trait	T-Chol–LDL-Chol–HDL-Chol	2	ALGA0013564	43603656	6
Clinical-biochemical trait	NEFA	2	ALGA0015164	115449913	7
Clinical-biochemical trait	LDL-Chol	3	ALGA0017541-ALGA0017674	12025480-12941569	8
Clinical-biochemical trait	LDL-Chol	3	ASGA0013487	12471350	8
Haematological trait	EOSINO	3	H3GA0009277	35094559	9
Clinical-biochemical trait	T-Chol–LDL-Chol–HDL-Chol	3	ASGA0016313	116718459	10
Clinical-biochemical trait	LDL-Chol<br>T-Chol	3	ASGA0016334-MARC0034240	117016694-117989682	10
Haematological trait	EOSINO	3	H3GA0010692	117043080	10
Clinical-biochemical trait	LDL-Chol<br>T-Chol	3	DIAS0000055	117295071	10
Clinical-biochemical trait	ALT	4	ALGA0029783	1175147	11
Clinical-biochemical trait	HDL-Chol	4	ALGA0022933-ALGA0023014	9035765-9963475	12
Clinical-biochemical trait	T-Chol<br>T-Chol–LDL-Chol–HDL-Chol	4	ALGA0022970	9713080	12
Haematological trait	NEUTRO	4	MARC0052177	77597154	13

Haematological trait	PLT	4	MARC0047043	94795752	14
Clinical-biochemical trait	Alb-Glob-ratio	5	H3GA0015245	5996798	15
Haematological trait	MCH<br>MCV<br>MCH–MCHC–MCV–RDW<br>RBC–HGB–HCT	5	MARC0069472	14820993	16
Haematological trait	RBC	5	M1GA0007649	15060402	16
Clinical-biochemical trait	UA	5	ALGA0031583-<br>MARC0036874	31022891-<br>31954627	17
Clinical-biochemical trait	UA	5	ALGA0031618	31257070	17
Clinical-biochemical trait	UA	5	ALGA0031630	31476339	17
Clinical-biochemical trait	T-Chol	5	ASGA0104003	80770124	18
Haematological trait	MCHC	5	MARC0044114	85947536	19
Haematological trait	RBC	5	ALGA0034012-<br>DIAS0000757	100071672-<br>100955310	20
Clinical-biochemical trait	Alb	6	MARC0028659-<br>DRGA0000307	88007685-<br>88895391	21
Clinical-biochemical trait	Alb	6	H3GA0052531	88193827	21
Haematological trait	WBC	6	DIAS0004496	97994715	22
Clinical-biochemical trait	T-Chol	7	MARC0003814	17166311	23
Clinical-biochemical trait	Alb–Alb-Glob-ratio	7	INRA0025193	38259165	24
Haematological trait	HGB<br>HCT	7	MARC0028659-<br>DRGA0000307	50022514-<br>50996708	25
Haematological trait	HGB	7	M1GA0010414-<br>ASGA0085536	52033314-<br>52890898	25
Haematological trait	EOSINO	7	H3GA0021970	61362991	26
Clinical-biochemical trait	Glu	7	ALGA0110857	101154686	27
Haematological trait	EOSINO	7	INRA0028736	118053616	28
Haematological trait	MCH–MCHC–MCV–RDW	7	ASGA0036974	120968439	29
Haematological trait	MCV	8	DRGA0008367	17423203	30
Haematological trait	MCV	8	ALGA0046671-<br>MARC0000247	17019764-<br>17986937	30
Clinical-biochemical trait	NEFA	9	ASGA0041146	5738251	31
Clinical-biochemical trait	UA	9	ALGA0051311	10886506	32

Clinical-biochemical trait	HDL-Chol	9		42015102-<br>42977109	33
Haematological trait	EOSINO	10	H3GA0030197	46331882	34
Haematological trait	MPV	12	ASGA0053310	13483964	35
Haematological trait	RDW	12	DIAS0000242	40010805	36
Haematological trait	HGB<br>RBC–HGB–HCT	13	CASI0007727	177262726	37
Haematological trait	MCH–MCHC–MCV–RDW	14	ASGA0060510	2891771	38
Haematological trait	MCH	14	M1GA0018108-<br>ASGA0060872	6142147-<br>6991699	38
Haematological trait	MCH<br>MCH–MCHC–MCV–RDW<br>RBC–HGB–HCT	14	H3GA0038580	6432121	38
Haematological trait	MCHC	14	H3GA0038597	6641352	38
Haematological trait	MCH	14	DRGA0013486-<br>DIAS0002359	8004489-<br>8994023	38
Clinical-biochemical trait	LDL-Chol	14	ALGA0077232-<br>ASGA0063118	43074710-<br>43957703	39
Clinical-biochemical trait	LDL-Chol	14	ALGA0077250	43459397	39
Clinical-biochemical trait	NEFA	14	DRGA0013970	64191357	40
Haematological trait	BASO	14	ALGA0078523-<br>MARC0109364	67039033-<br>67985525	41
Haematological trait	BASO	14	INRA0044634	66411511	41
Haematological trait	BASO	14	DIAS0002104	66650724	41
Haematological trait	BASO	14	ASGA0064211	67179643	41
Haematological trait	BASO	14	MARC0095482	67221608	41
Haematological trait	BASO	14	DRGA0014018	67429030	41
Haematological trait	BASO	14	H3GA0052373	67551686	41
Haematological trait	BASO	14	CASI0008086	68966102	41
Haematological trait	BASO	14	DRGA0014066	69323867	41
Haematological trait	BASO	14	ALGA0078633	69589926	41
Haematological trait	BASO	14	MARC0092344	69865089	41
Haematological trait	BASO	14	MARC0006045	69943312	41
Haematological trait	BASO	14	CASI0008356	70268234	41
Haematological trait	BASO	14	MARC0019955	71087715	41

Haematological trait	BASO	14	INRA0044866	71116678	41
Haematological trait	BASO	14	ALGA0079529	71958965	41
Haematological trait	BASO	14	ALGA0079530	71996220	41
Haematological trait	BASO	14	ALGA0079583	72478930	41
Haematological trait	BASO	14	DIAS0001570	72544071	41
Haematological trait	BASO	14	MARC0090899	72704456	41
Haematological trait	BASO	14	ALGA0078729	73118420	41
Haematological trait	BASO	14	ALGA0078757	73396066	41
Clinical-biochemical trait	AST<br>CK–AST	14	INRA0046629	110424327	42
Clinical-biochemical trait	T-Bil	14	ASGA0067171	130438475	43
Haematological trait	MONO	15	ALGA0084320	19853411	44
Haematological trait	RDW	16	ALGA0090171	33206877	45
Clinical-biochemical trait	TG	16	ASGA0073326	47077521	46
Haematological trait	RDW	16	ALGA0091410	67367349	47
Clinical-biochemical trait	T-Bil	17	ALGA0094849	37006714	48
Clinical-biochemical trait	T-Prot	17	M1GA0022271	52958671	49
Haematological trait	HGB	18	MARC0005874-<br>MARC0014887	36020050-<br>36959194	50
Haematological trait	HCT<br>HGB	18	DRGA0017710	36749906	50
Clinical-biochemical trait	ALT	18	DRGA0017057-<br>MARC0055353	48008832-<br>48983680	51
Clinical-biochemical trait	ALT	18	ALGA0098672	48131574	51
Clinical-biochemical trait	ALT	NW_018084979.1	ASGA0101473-<br>ALGA0124558	2062221-<br>2846867	52
Clinical-biochemical trait	ALT	NW_018084979.1	H3GA0055255-<br>ASGA0037286	3094869-<br>3301399	52
Clinical-biochemical trait	ALT	NW_018084979.1	H3GA0023887	3163232	52

[1]Full names are reported in Supplementary Table S1.

[2]SSC = *Sus scrofa* chromosome. NW_018084979.1 is an unassembled scaffold.

[3]Position (bp): position based on the Sscrofa11.1 reference genome.

Supplementary Table S5. Genomic inflation factor (λ) retrieved from the single marker single trait genome-wide association scans.

Parameters (units)	Abbreviation	Genomic inflation factor (λ)
**Haematological parameters**		
Erythrocyte traits		
Red blood cell count (cells/μL)	RBC	1.00899
Hemoglobin (g/dL)	HGB	0.98373
Hematocrit (%)	HCT	0.97911
Mean corpuscular volume (fL)	MCV	1.00011
Mean corpuscular hemoglobin (pg)	MCH	0.99392
Mean corpuscular hemoglobin	MCHC	0.99264
Red cell distribution width (%)	RDW	1.04305
Leukocyte traits		
White blood cell count (cells/μL)	WBC	1.01203
Lymphocyte count (cells/μL)	LYMPHO	1.00922
Neutrophil count (cells/μL)	NEUTRO	0.99688
Eosinophil count (cells/μL)	EOSINO	1.02923
Basophil count (cells/μL)	BASO	0.99029
Monocyte count (cells/μL)	MONO	0.99101
Platelet traits		
Platelet count (cells/μL)	PLT	0.99589
Mean platelet volume (fL)	MPV	0.99940
**Clinical-biochemical parameters**		
Lipid related traits		
Total cholesterol (mg/dL)	T-Chol	1.01309
High-density lipoprotein cholesterol (mg/dL)	HDL-Chol	1.00721
Low-density lipoprotein cholesterol (mg/dL)	LDL-Chol	1.00948
Triglycerides (mg/dL)	TG	0.98329
Non-esterified fatty acids (mmol/L)	NEFA	1.00507
Metabolism and protein related traits		
Glucose (mg/dL)	Glu	1.01359
Urea (mg/dL)	UA	1.01742
Total bilirubin (mg/dL)	T-Bil	1.05034
Total proteins (g/dL)	T-Prot	1.00939
Albumin (g/dL)	Alb	0.99293
Albumin/Globulines ratio	Alb-Glob-ratio	1.00591
Enzyme traits		
Alkaline phosphatase (u/L)	ALP	1.00305
Creatine kinase (u/dL)	CK	1.00325
Alanine aminotransferase (u/dL)	ALT	0.98242
Aspartate aminotransferase (u/dL)	AST	0.99965

Supplementary Figure S1. Manhattan plots displaying the results of the single marker genome wide association studies for the 30 blood parameters. Trait abbreviations are defined in Supplementary Table S1.

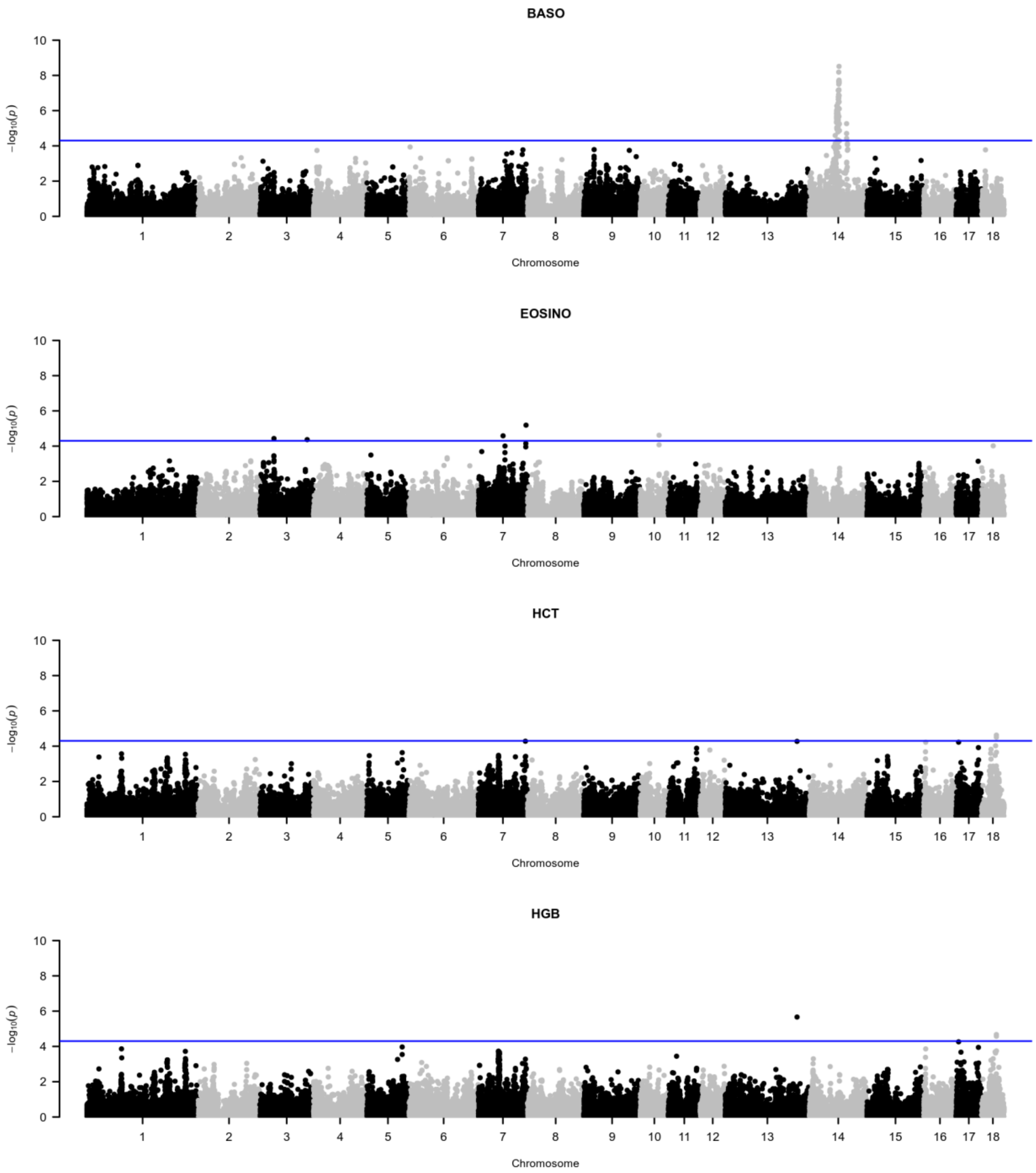

LYMPHO

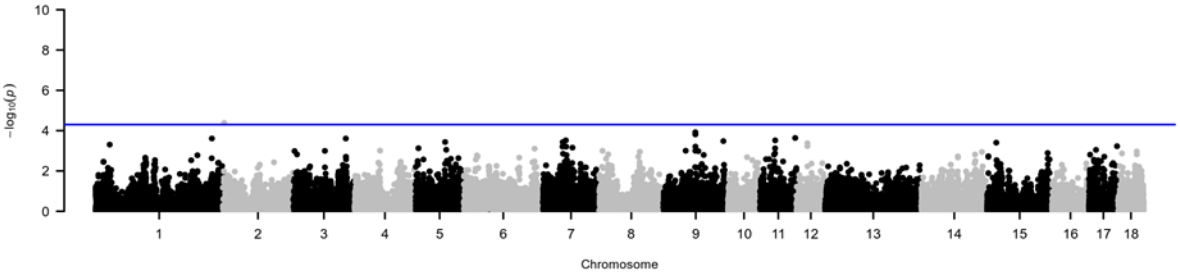

LIMPHO

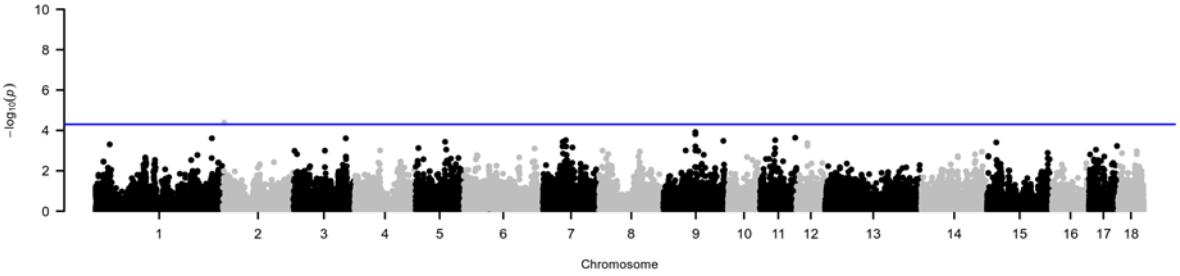

MCH

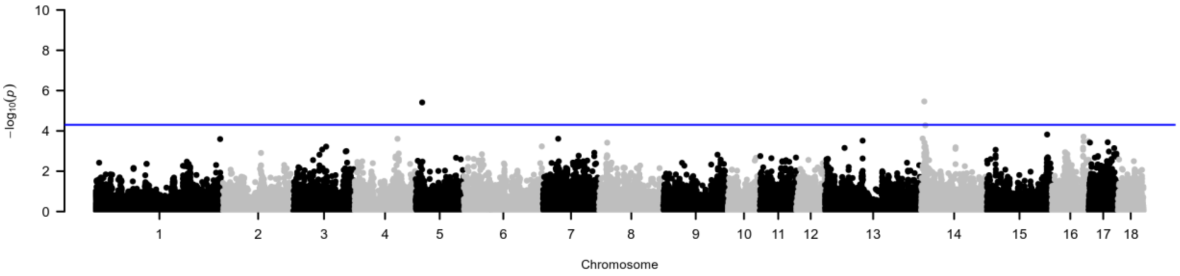

MCHC

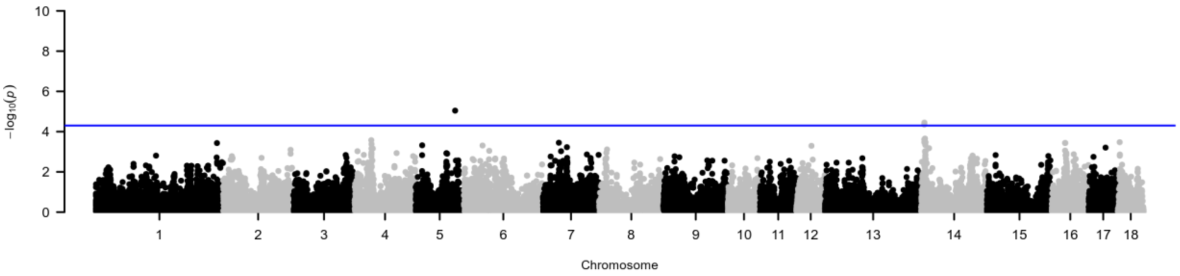

MONO

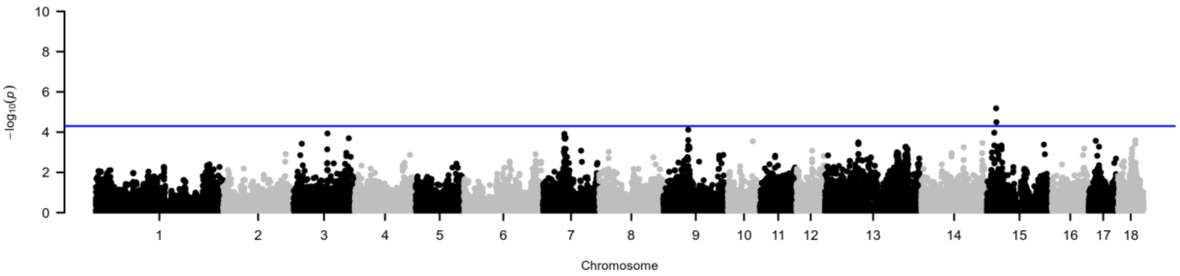

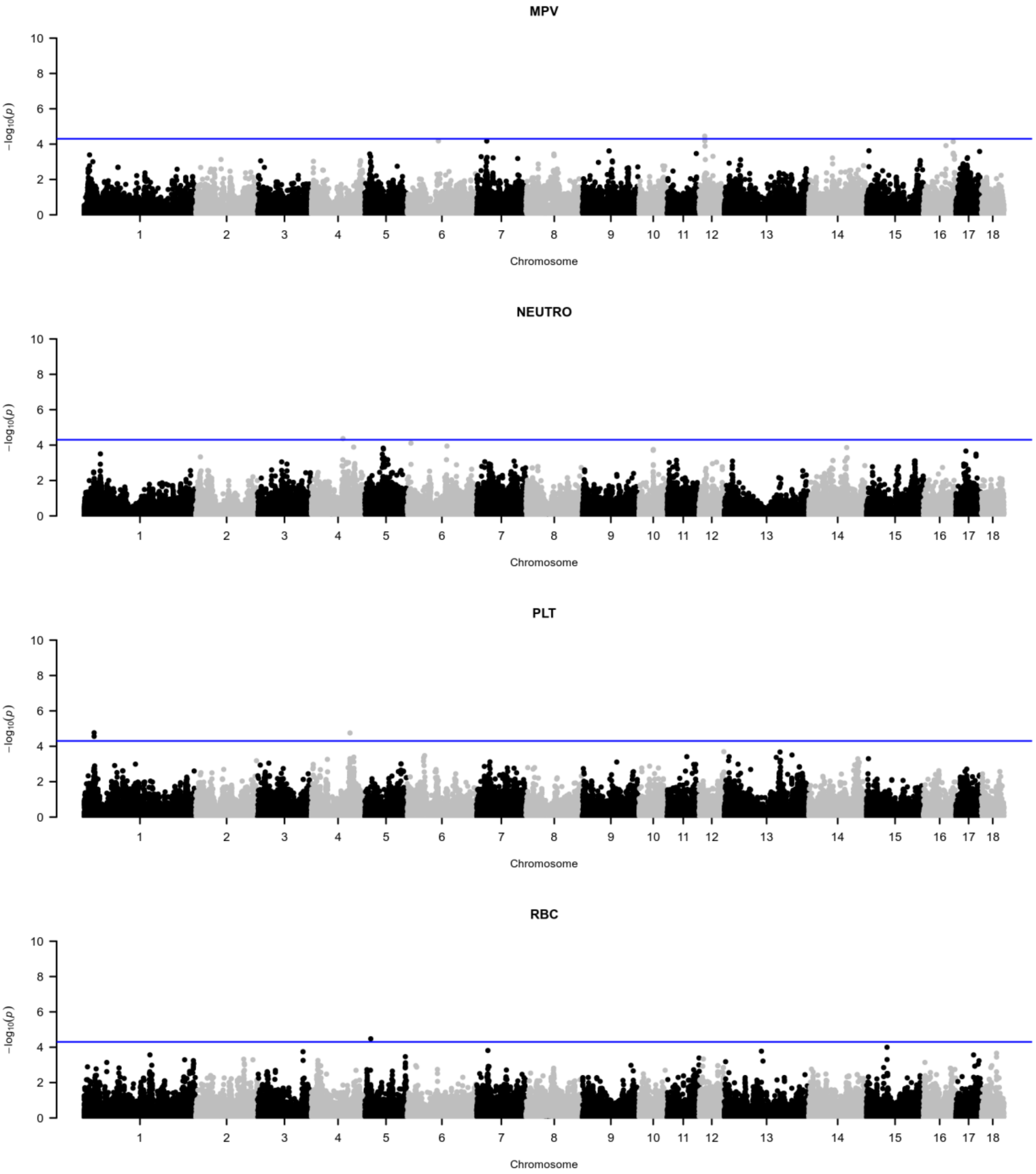
MPV
NEUTRO
PLT
RBC
−log10(p)
Chromosome
1 2 3 4 5 6 7 8 9 10 11 12 13 14 15 16 17 18

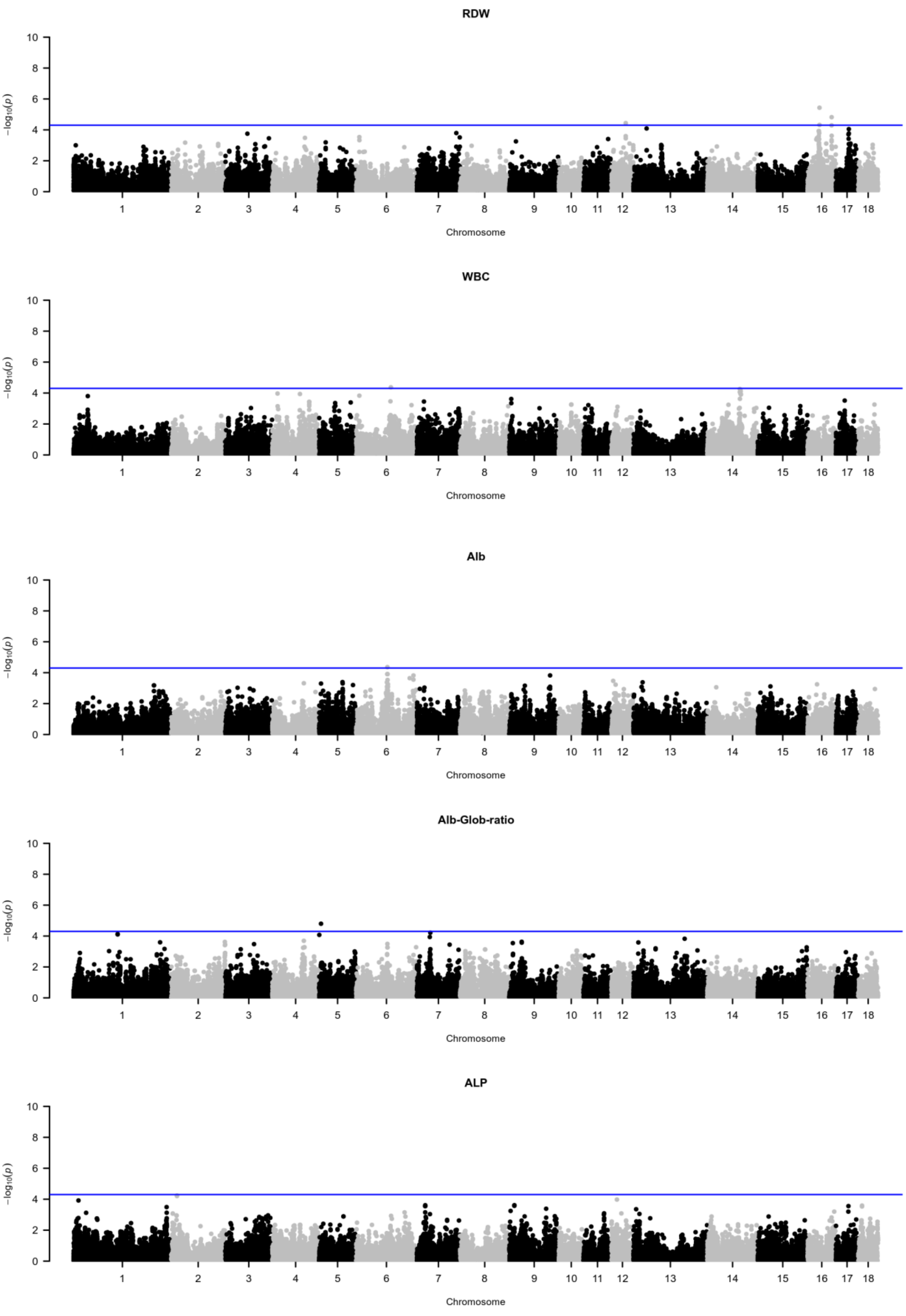
RDW
WBC
Alb
Alb-Glob-ratio
ALP
−log10(p)
Chromosome
1 2 3 4 5 6 7 8 9 10 11 12 13 14 15 16 17 18

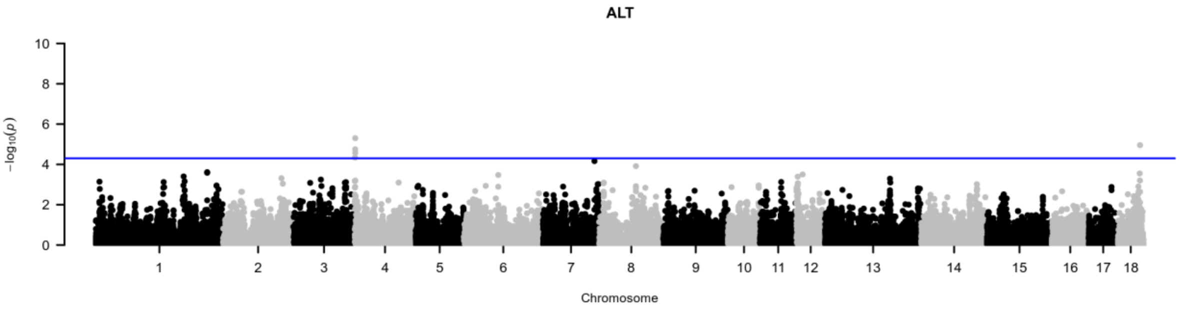
ALT
−log10(p)
Chromosome
1 2 3 4 5 6 7 8 9 10 11 12 13 14 15 16 17 18

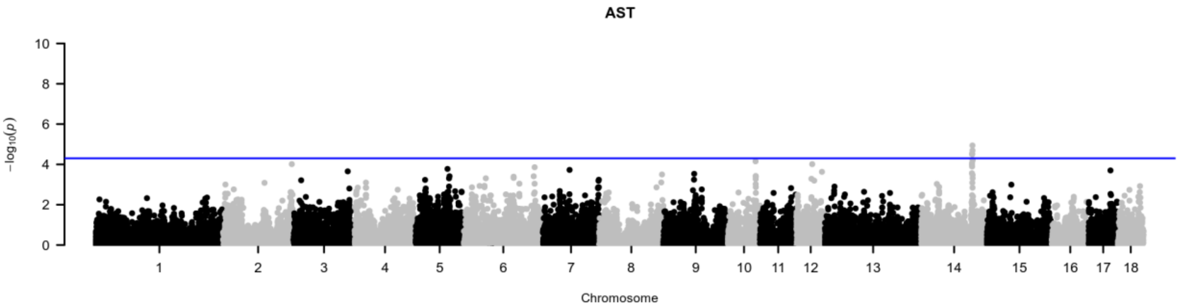
AST
−log10(p)
Chromosome
1 2 3 4 5 6 7 8 9 10 11 12 13 14 15 16 17 18

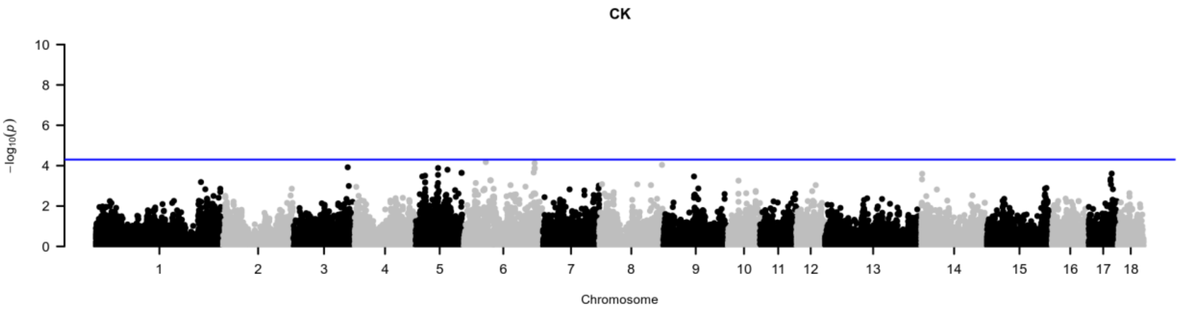
CK
−log10(p)
Chromosome
1 2 3 4 5 6 7 8 9 10 11 12 13 14 15 16 17 18

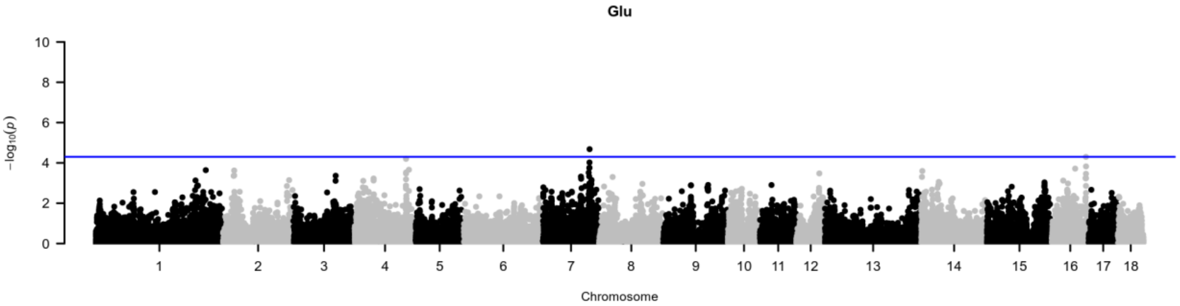
Glu
−log10(p)
Chromosome
1 2 3 4 5 6 7 8 9 10 11 12 13 14 15 16 17 18

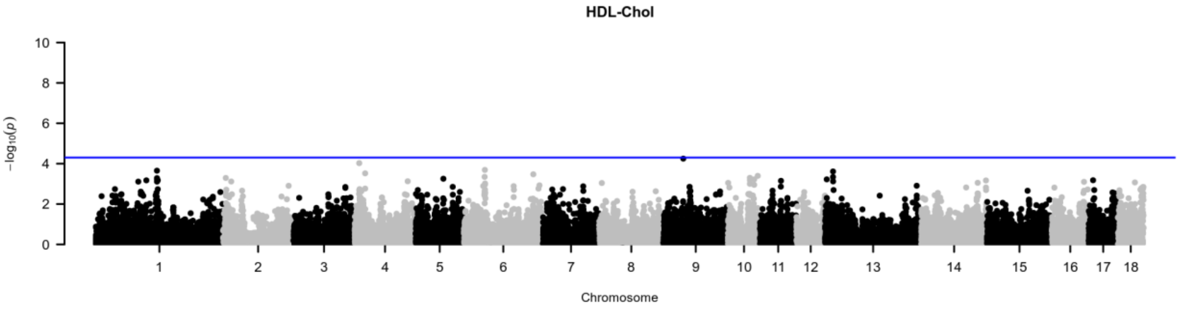
HDL-Chol
−log10(p)
Chromosome
1 2 3 4 5 6 7 8 9 10 11 12 13 14 15 16 17 18

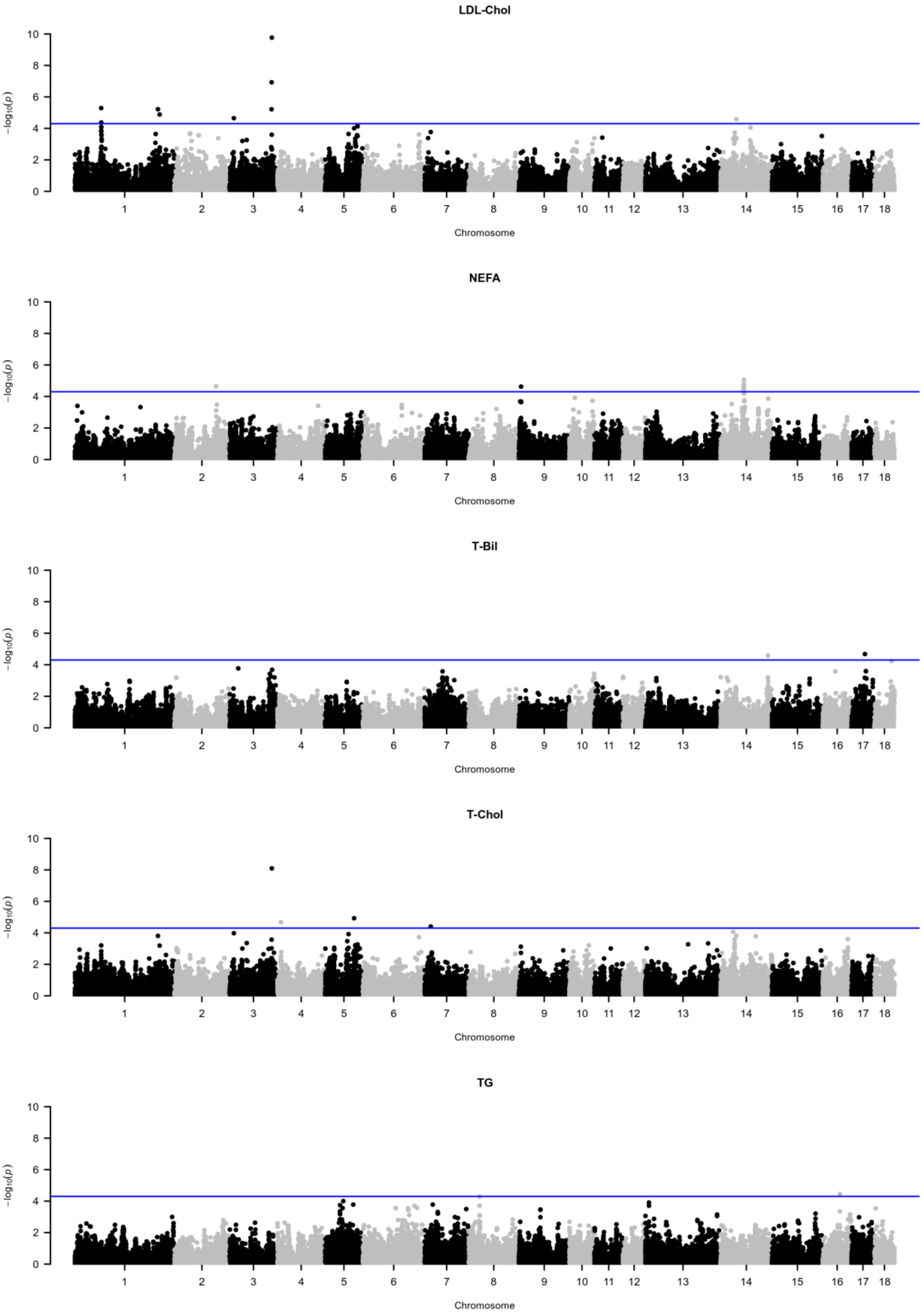

LDL-Chol
NEFA
T-Bil
T-Chol
TG
$-\log_{10}(p)$
0 2 4 6 8 10
1 2 3 4 5 6 7 8 9 10 11 12 13 14 15 16 17 18
Chromosome

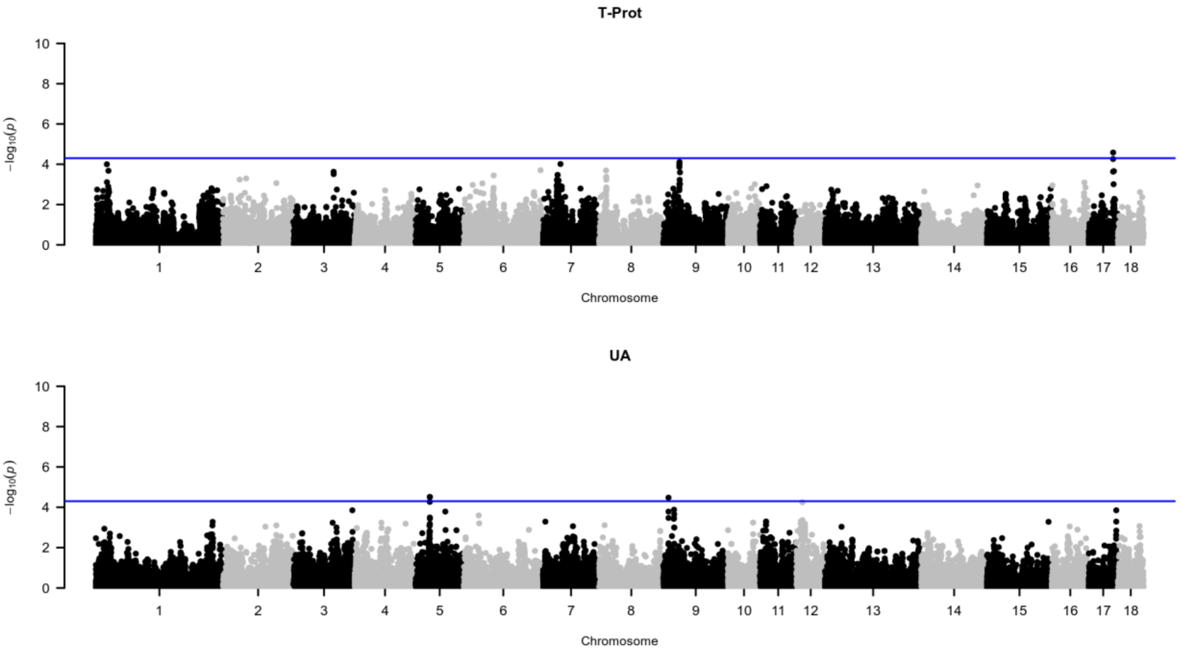

T-Prot
10
8
6
4
2
0
−log10(p)
1
2
3
4
5
6
7
8
9
10
11
12
13
14
15
16
17
18
Chromosome
UA
10
8
6
4
2
0
−log10(p)
1
2
3
4
5
6
7
8
9
10
11
12
13
14
15
16
17
18
Chromosome

Supplementary Figure S2. Manhattan plots displaying the results of the multivariate genome wide association studies for the six sets of blood parameters. Trait abbreviations are defined in Supplementary Table S1.

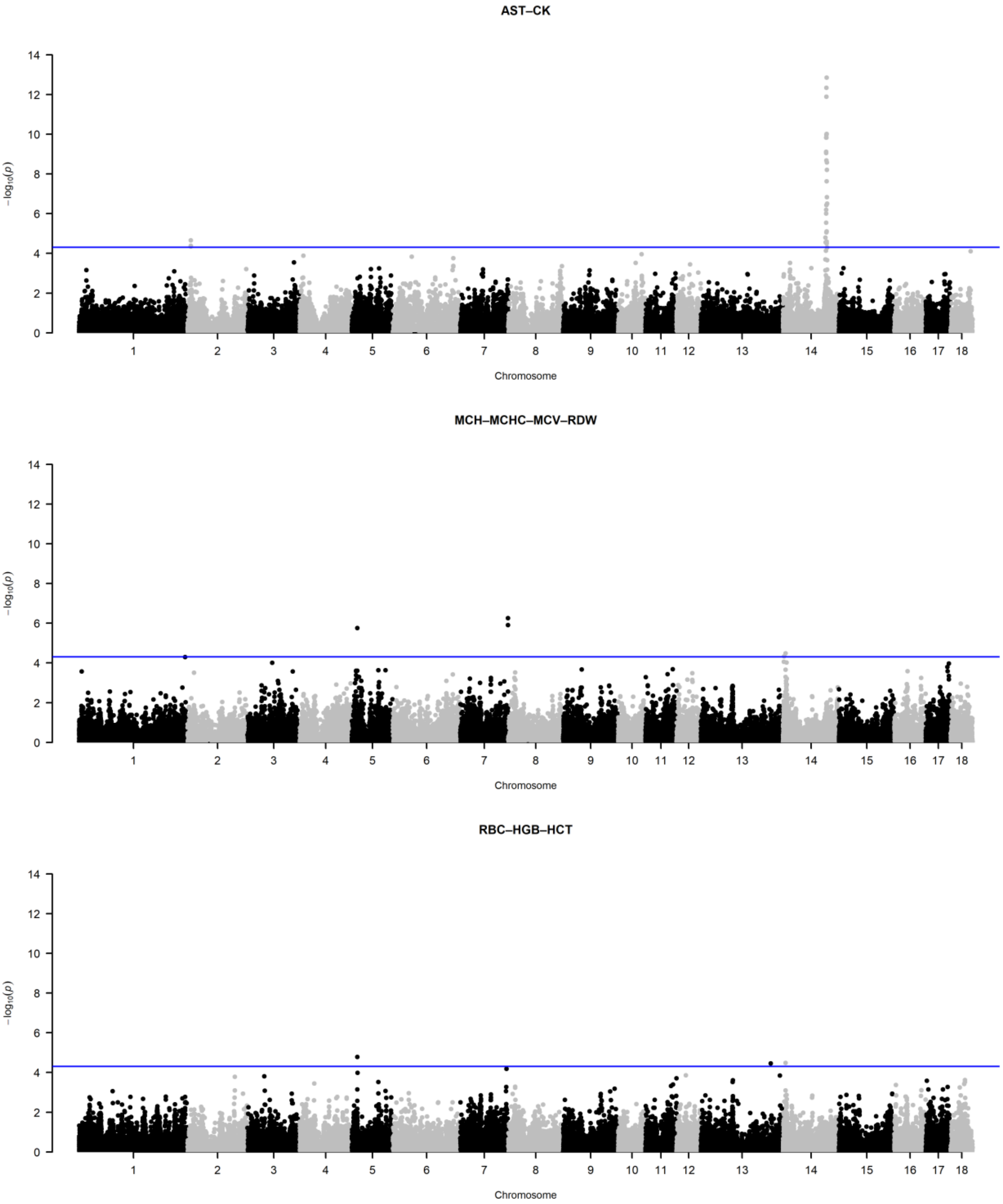

T-Chol–LDL-Chol–HDL-Chol

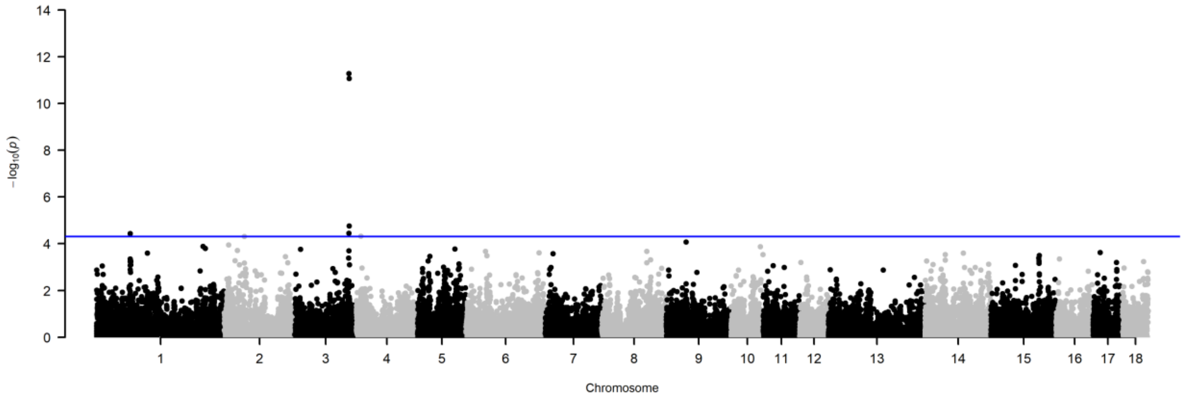

T-prot–Alb–Alb-Glob-ratio

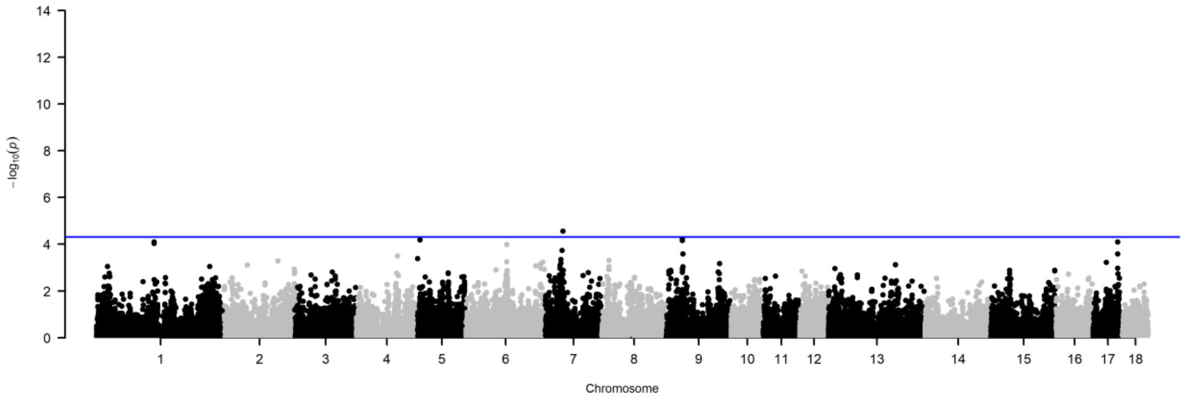

Supplementary Figure S3. Manhattan plots displaying the results of the Bayesian genome wide association studies for the 30 blood parameters. Trait abbreviations are defined in Supplementary Table S1.

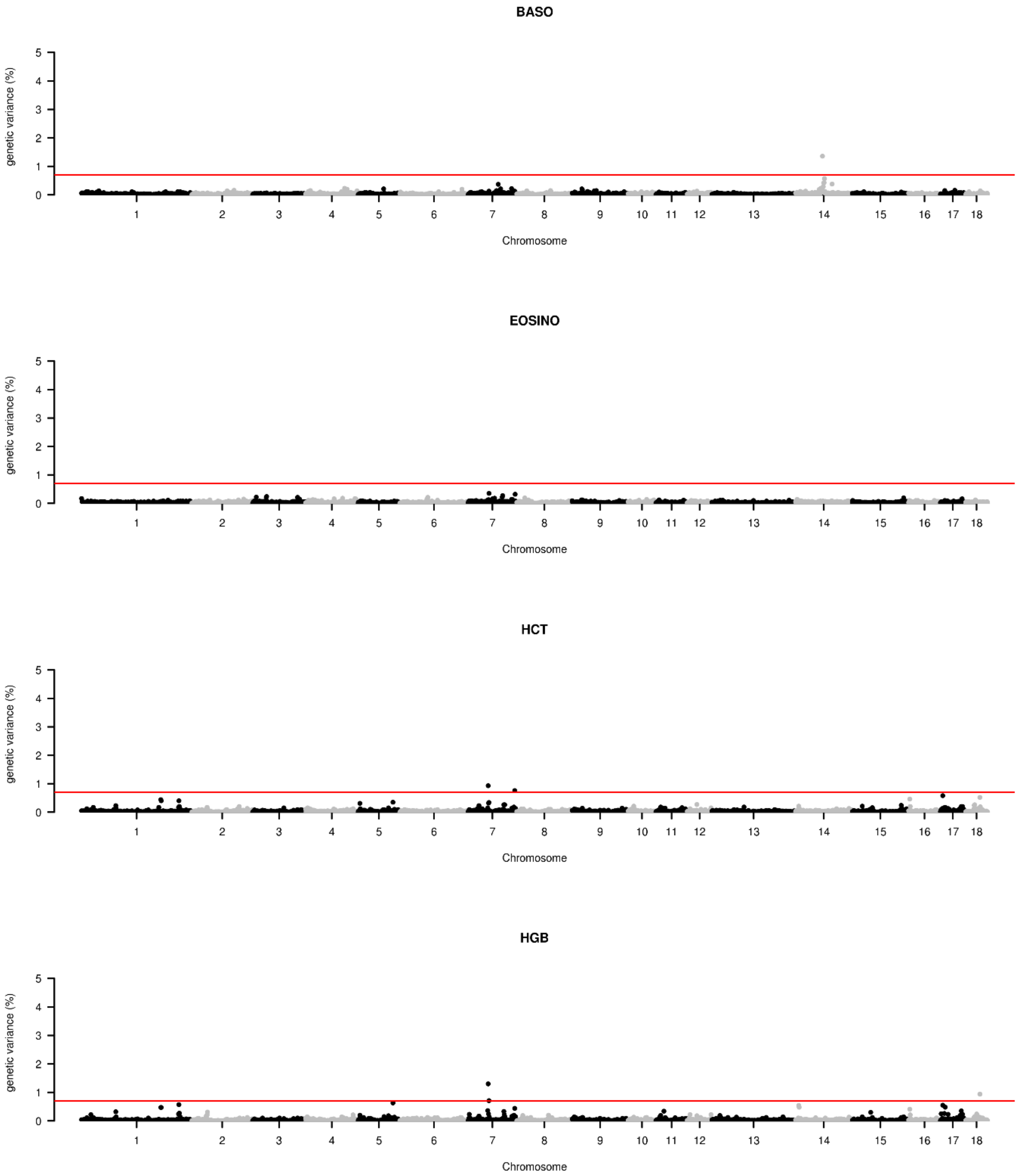

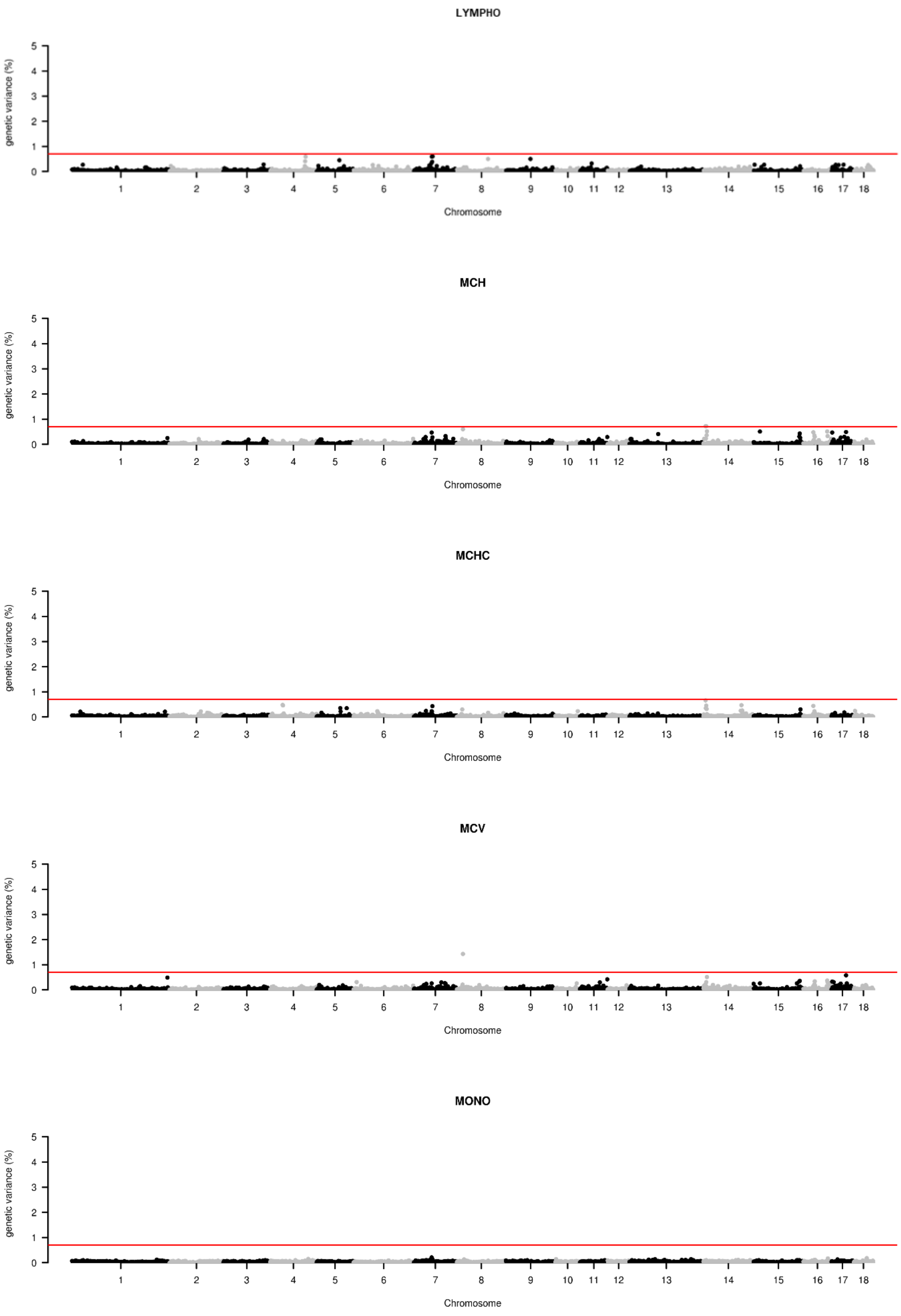

LYMPHO
genetic variance (%)
0
1
2
3
4
5
1
2
3
4
5
6
7
8
9
10
11
12
13
14
15
16
17
18
Chromosome
MCH
genetic variance (%)
Chromosome
MCHC
genetic variance (%)
Chromosome
MCV
genetic variance (%)
Chromosome
MONO
genetic variance (%)
Chromosome

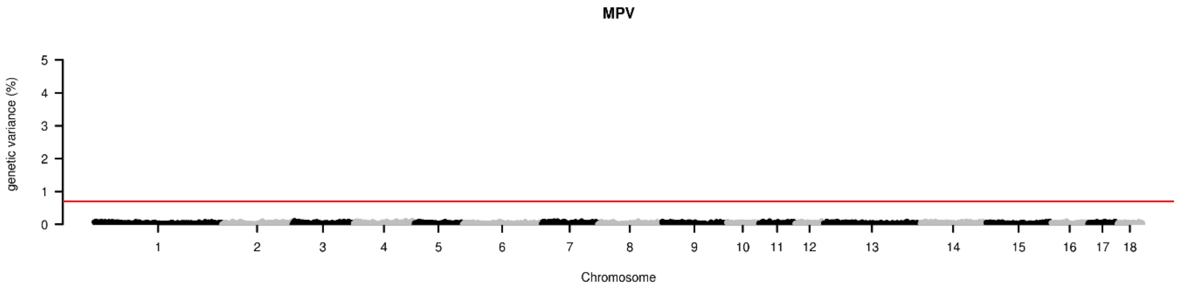
MPV
genetic variance (%)
Chromosome

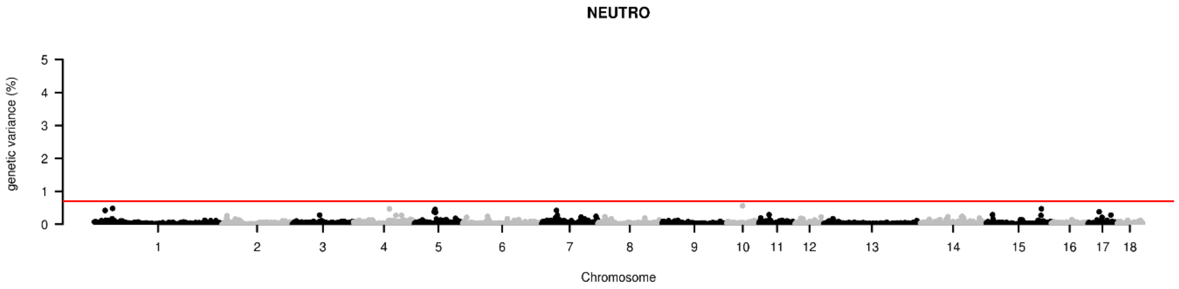
NEUTRO
genetic variance (%)
Chromosome

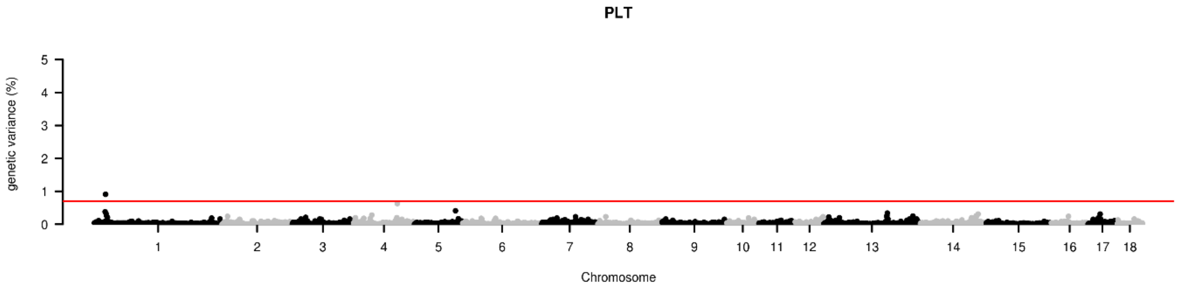
PLT
genetic variance (%)
Chromosome

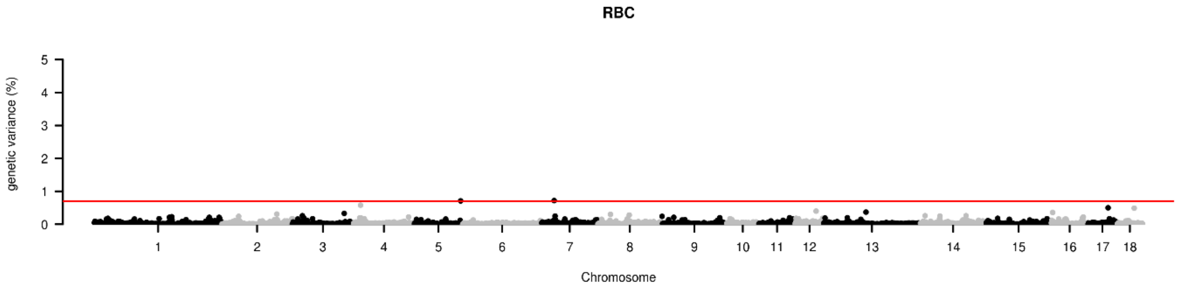
RBC
genetic variance (%)
Chromosome

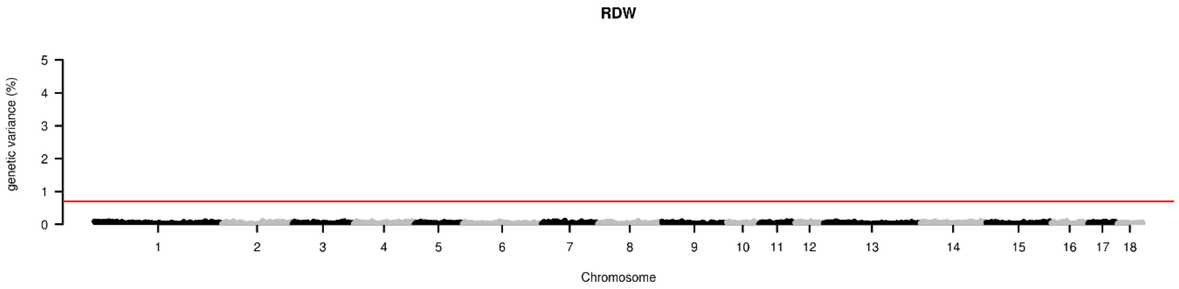
RDW
genetic variance (%)
0 1 2 3 4 5
1 2 3 4 5 6 7 8 9 10 11 12 13 14 15 16 17 18
Chromosome

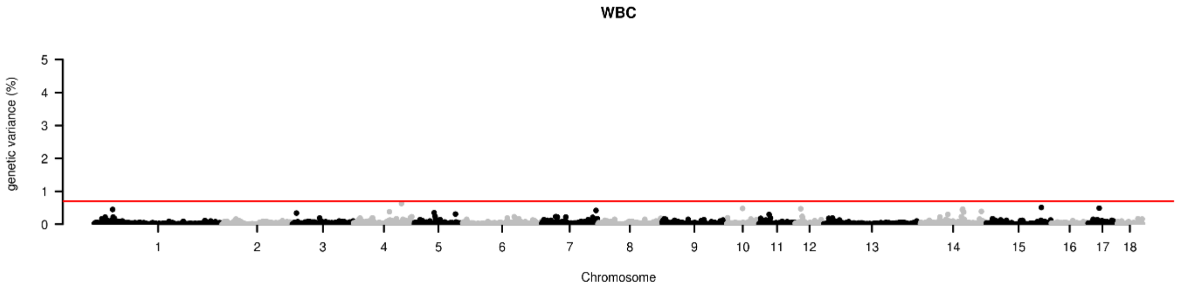
WBC
genetic variance (%)
0 1 2 3 4 5
1 2 3 4 5 6 7 8 9 10 11 12 13 14 15 16 17 18
Chromosome

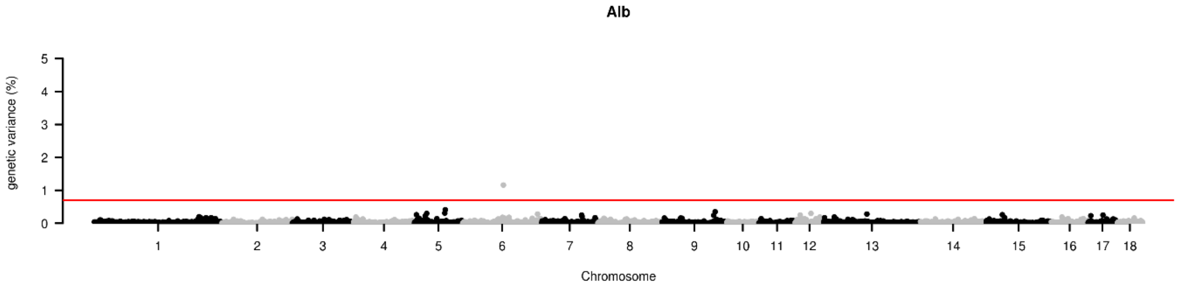
Alb
genetic variance (%)
0 1 2 3 4 5
1 2 3 4 5 6 7 8 9 10 11 12 13 14 15 16 17 18
Chromosome

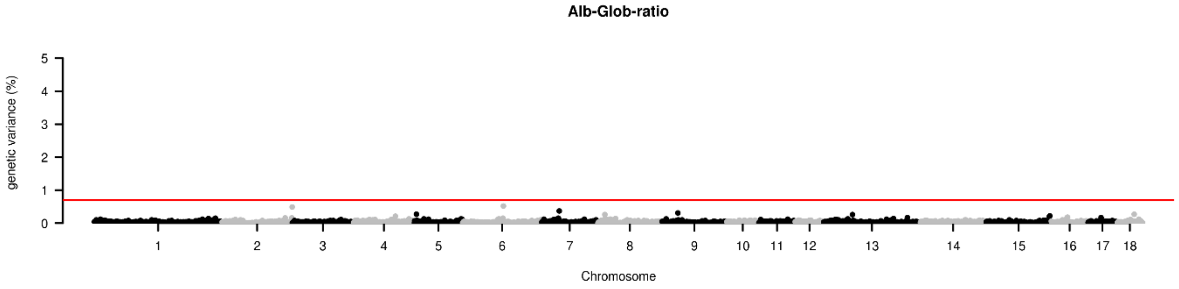
Alb-Glob-ratio
genetic variance (%)
0 1 2 3 4 5
1 2 3 4 5 6 7 8 9 10 11 12 13 14 15 16 17 18
Chromosome

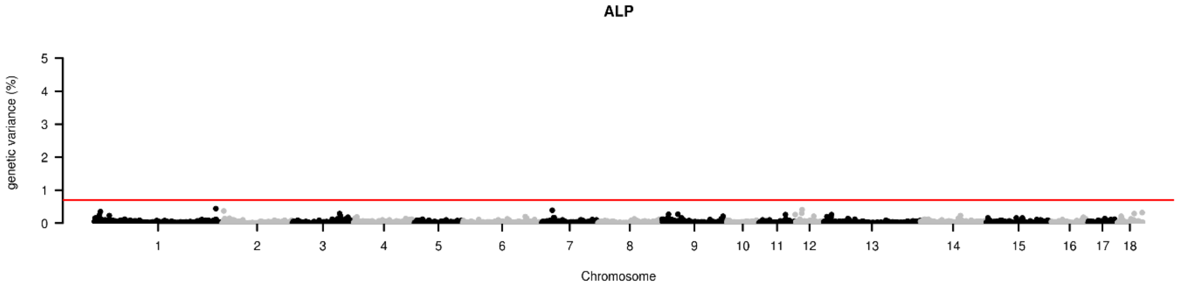
ALP
genetic variance (%)
0 1 2 3 4 5
1 2 3 4 5 6 7 8 9 10 11 12 13 14 15 16 17 18
Chromosome

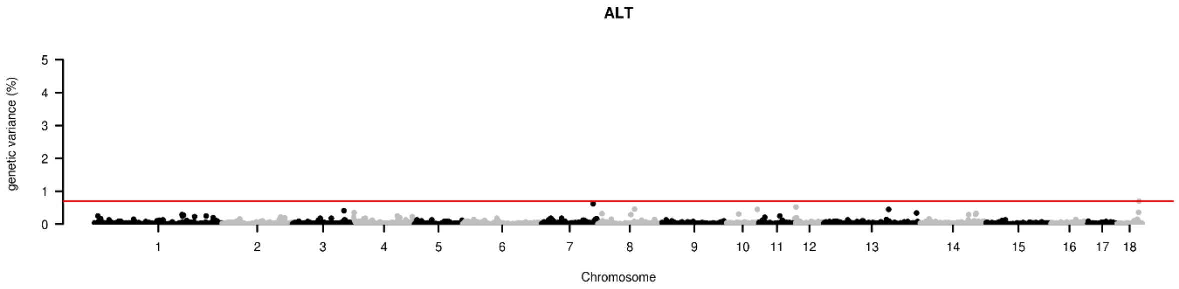
ALT
genetic variance (%)
0
1
2
3
4
5
1
2
3
4
5
6
7
8
9
10
11
12
13
14
15
16
17
18
Chromosome

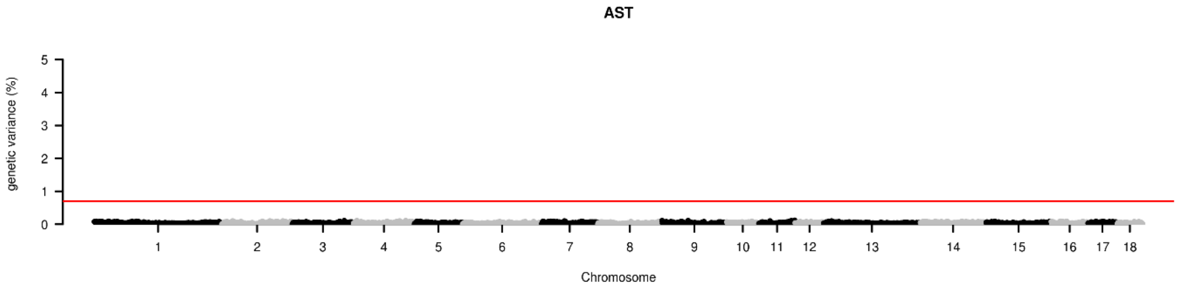
AST
genetic variance (%)
0
1
2
3
4
5
1
2
3
4
5
6
7
8
9
10
11
12
13
14
15
16
17
18
Chromosome

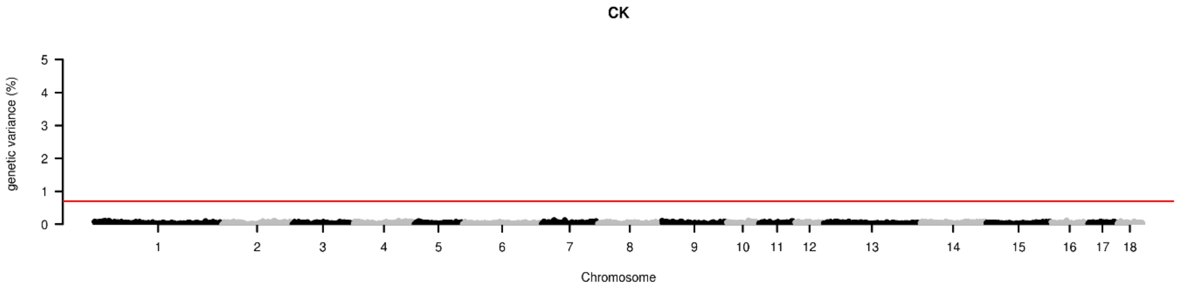
CK
genetic variance (%)
0
1
2
3
4
5
1
2
3
4
5
6
7
8
9
10
11
12
13
14
15
16
17
18
Chromosome

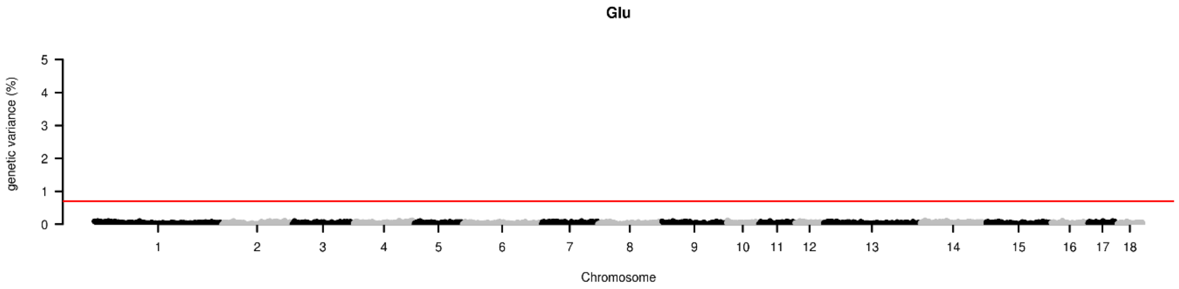
Glu
genetic variance (%)
0
1
2
3
4
5
1
2
3
4
5
6
7
8
9
10
11
12
13
14
15
16
17
18
Chromosome

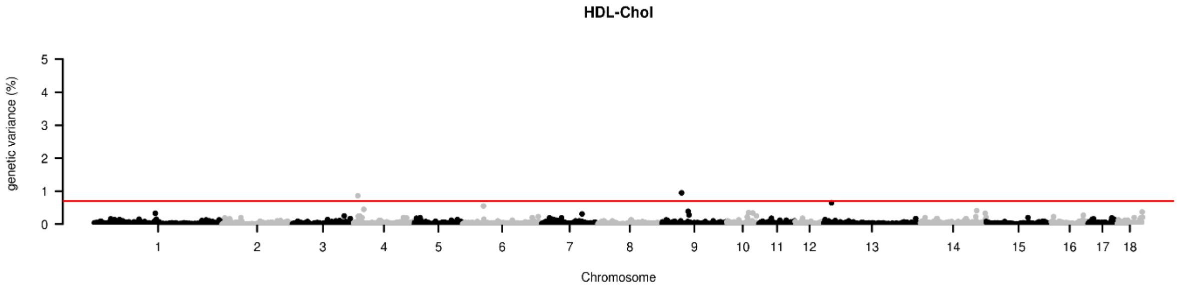
HDL-Chol
genetic variance (%)
0
1
2
3
4
5
1
2
3
4
5
6
7
8
9
10
11
12
13
14
15
16
17
18
Chromosome

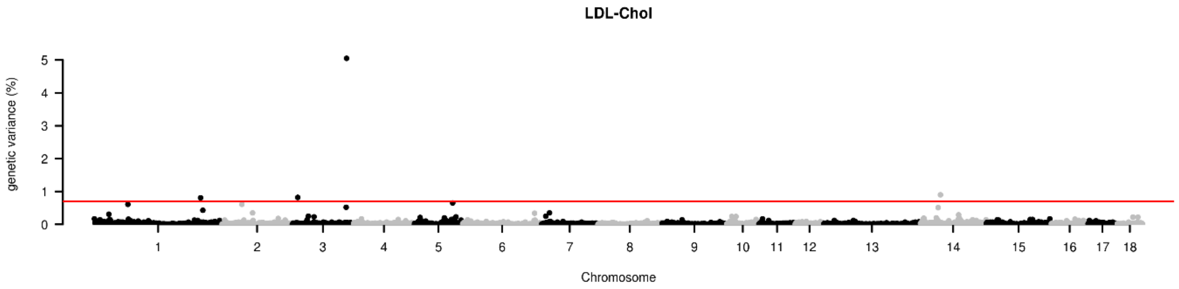
LDL-Chol
genetic variance (%)
Chromosome

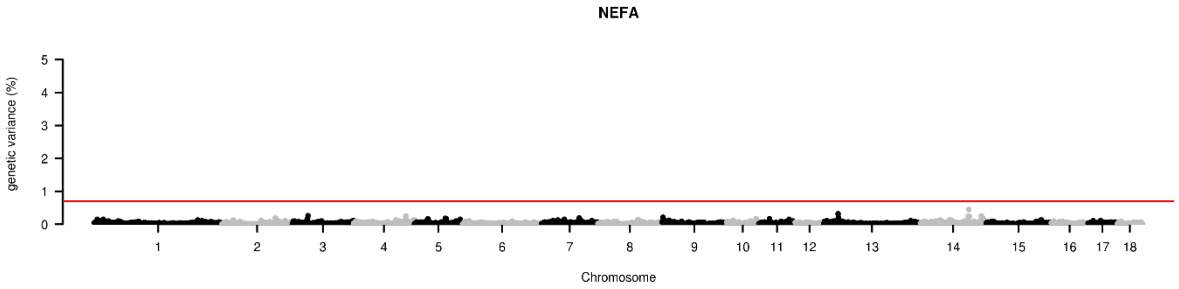
NEFA
genetic variance (%)
Chromosome

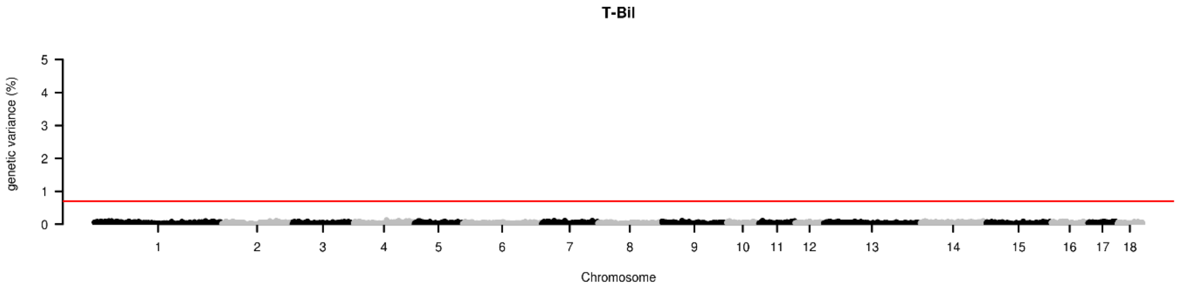
T-Bil
genetic variance (%)
Chromosome

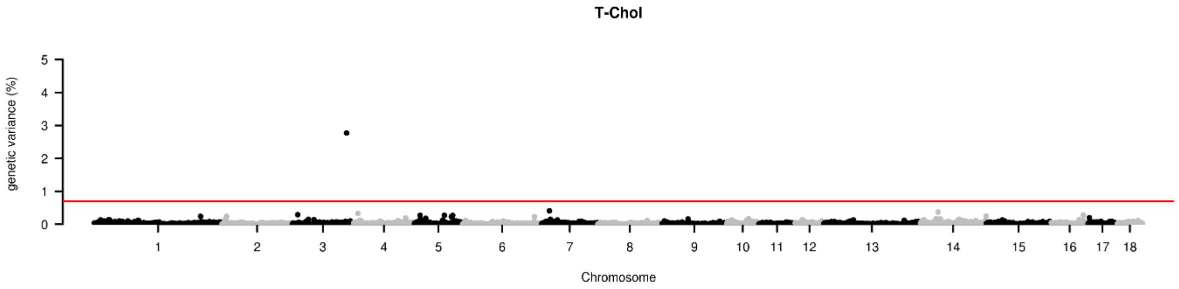
T-Chol
genetic variance (%)
Chromosome

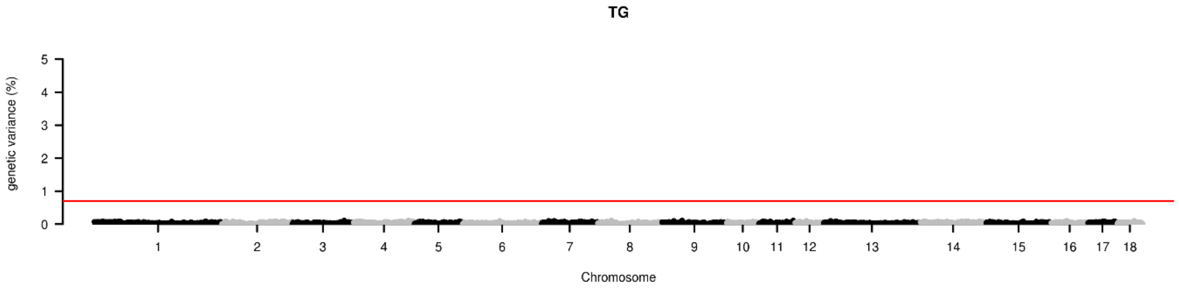
TG
genetic variance (%)
Chromosome

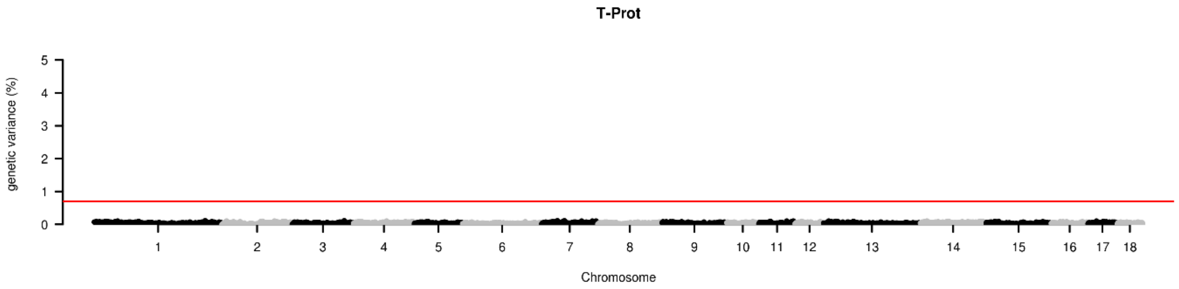
T-Prot
genetic variance (%)
0
1
2
3
4
5
1 2 3 4 5 6 7 8 9 10 11 12 13 14 15 16 17 18
Chromosome

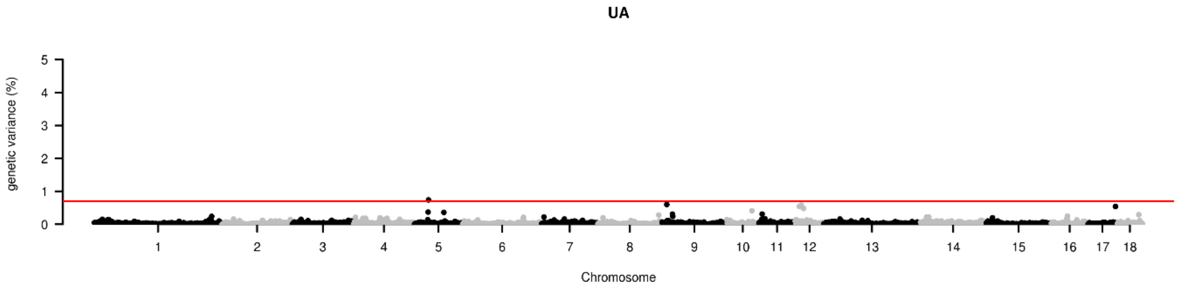
UA
genetic variance (%)
0
1
2
3
4
5
1 2 3 4 5 6 7 8 9 10 11 12 13 14 15 16 17 18
Chromosome

Supplementary Figure S4. QQ-plots of the single marker genome wide association studies for the 30 blood parameters Trait abbreviations are defined in Supplementary Table S1.

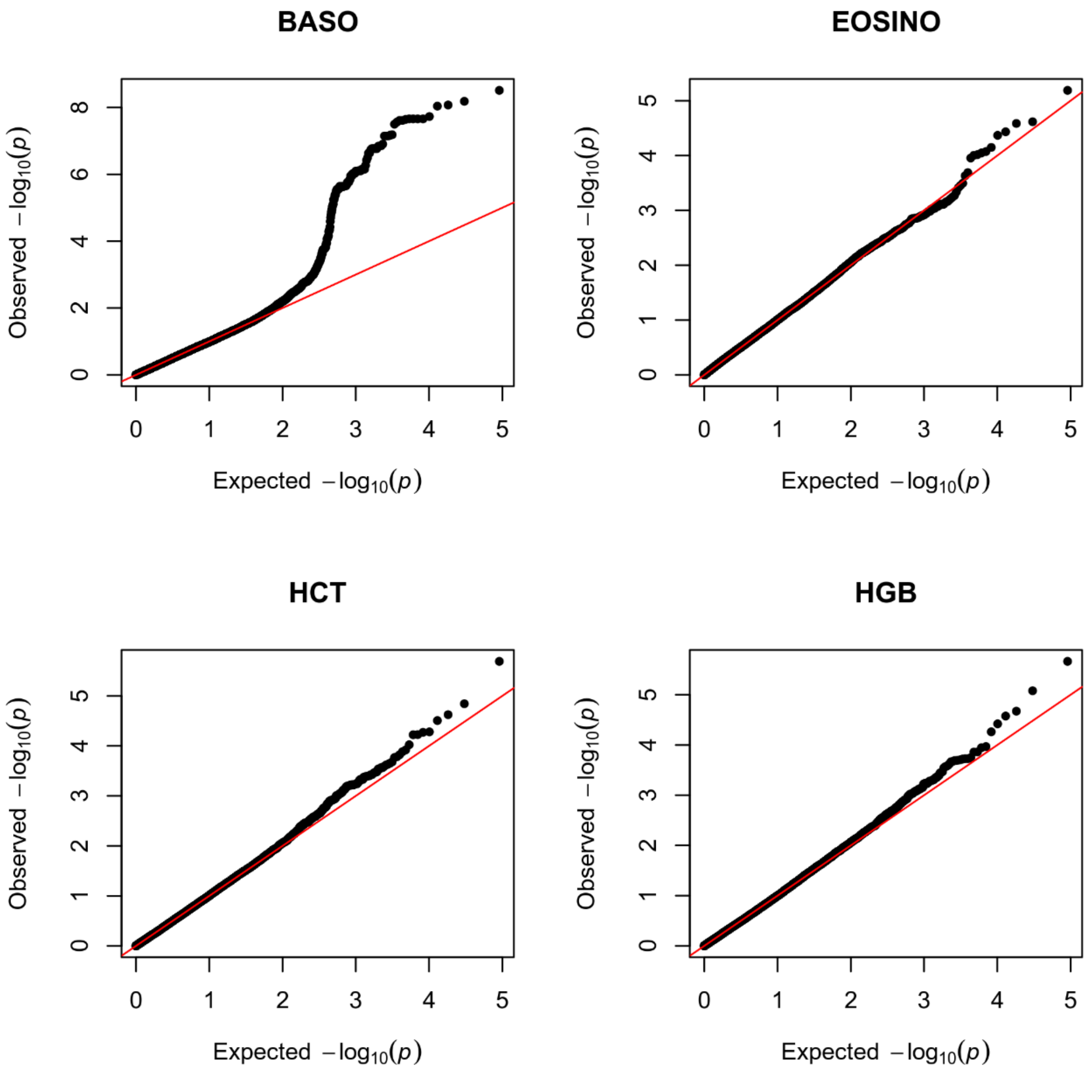

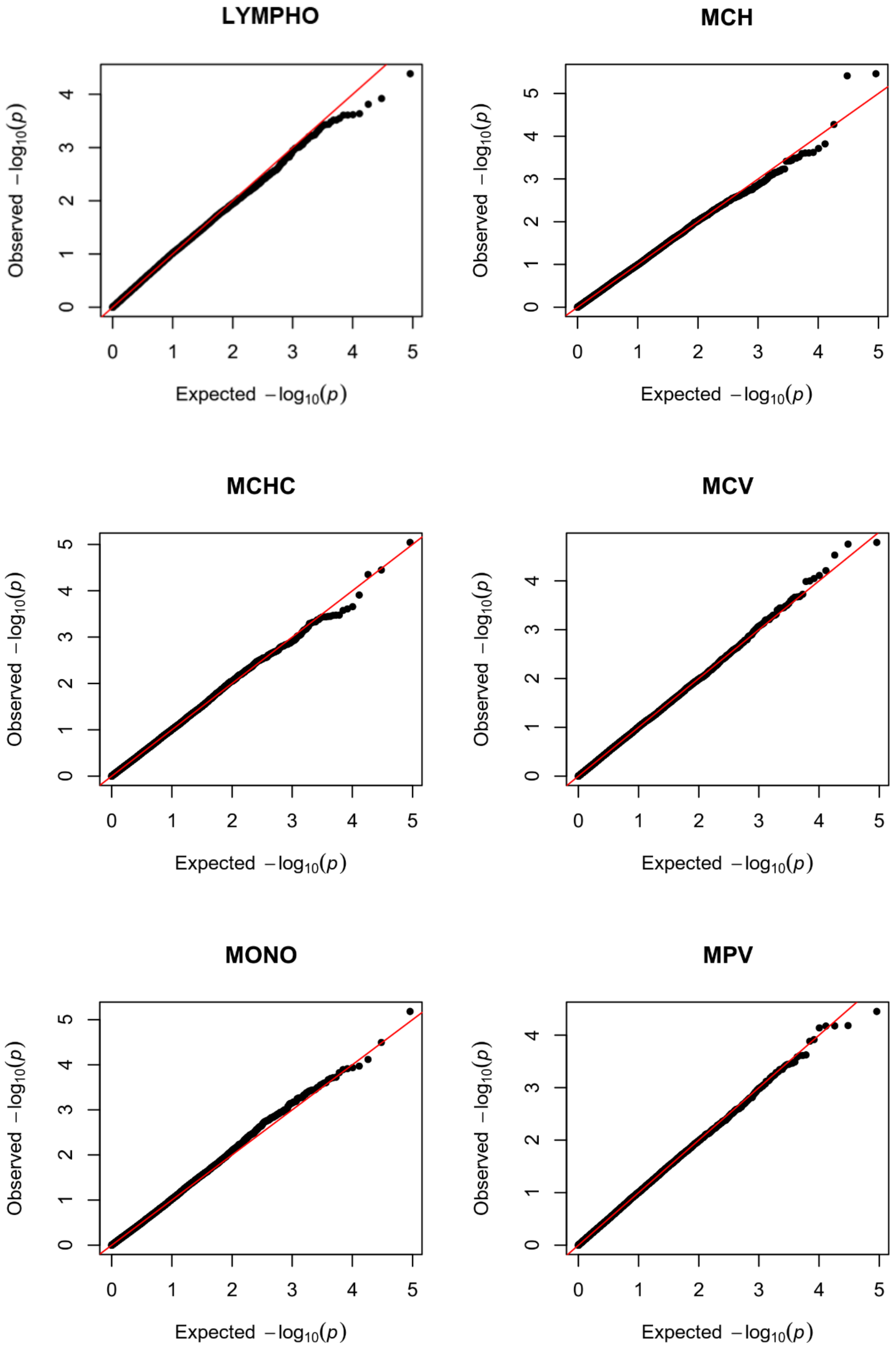

LYMPHO
MCH
MCHC
MCV
MONO
MPV
Observed $-\log_{10}(p)$
Expected $-\log_{10}(p)$

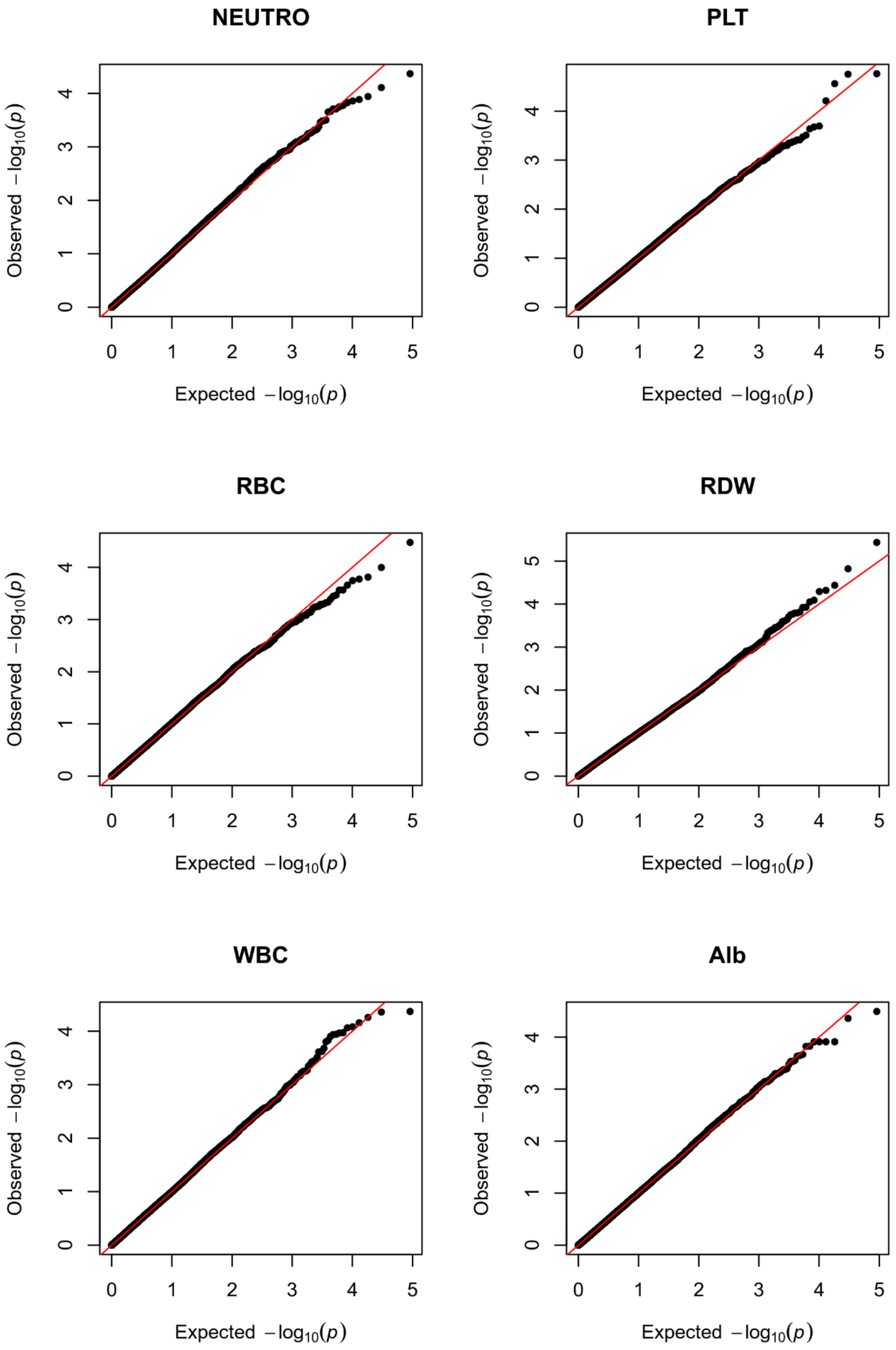

NEUTRO
PLT
RBC
RDW
WBC
Alb
Observed −log10(p)
Expected −log10(p)
0
1
2
3
4
5

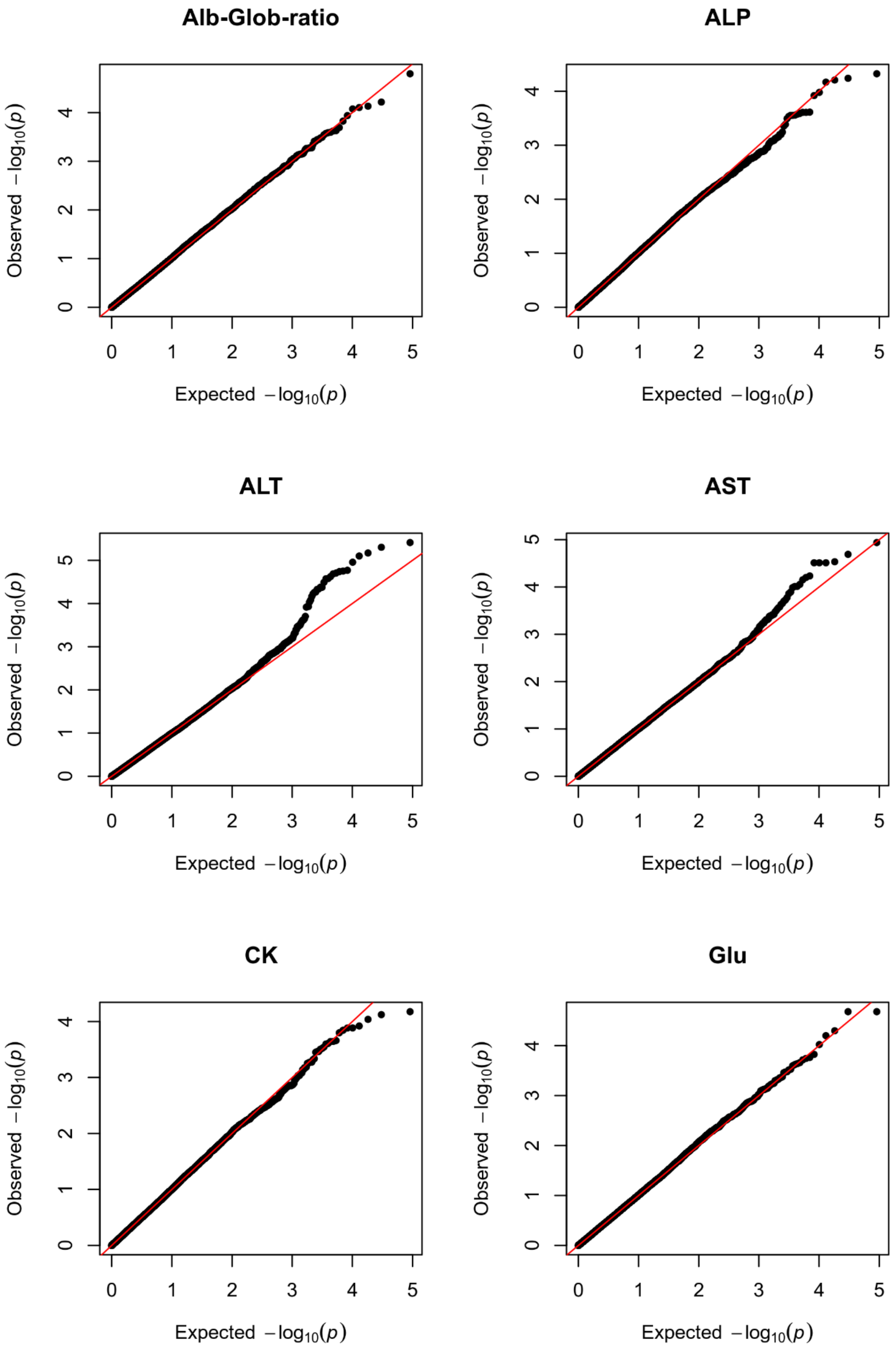
Alb-Glob-ratio
ALP
ALT
AST
CK
Glu
Observed $-\log_{10}(p)$
Expected $-\log_{10}(p)$
0
1
2
3
4
5

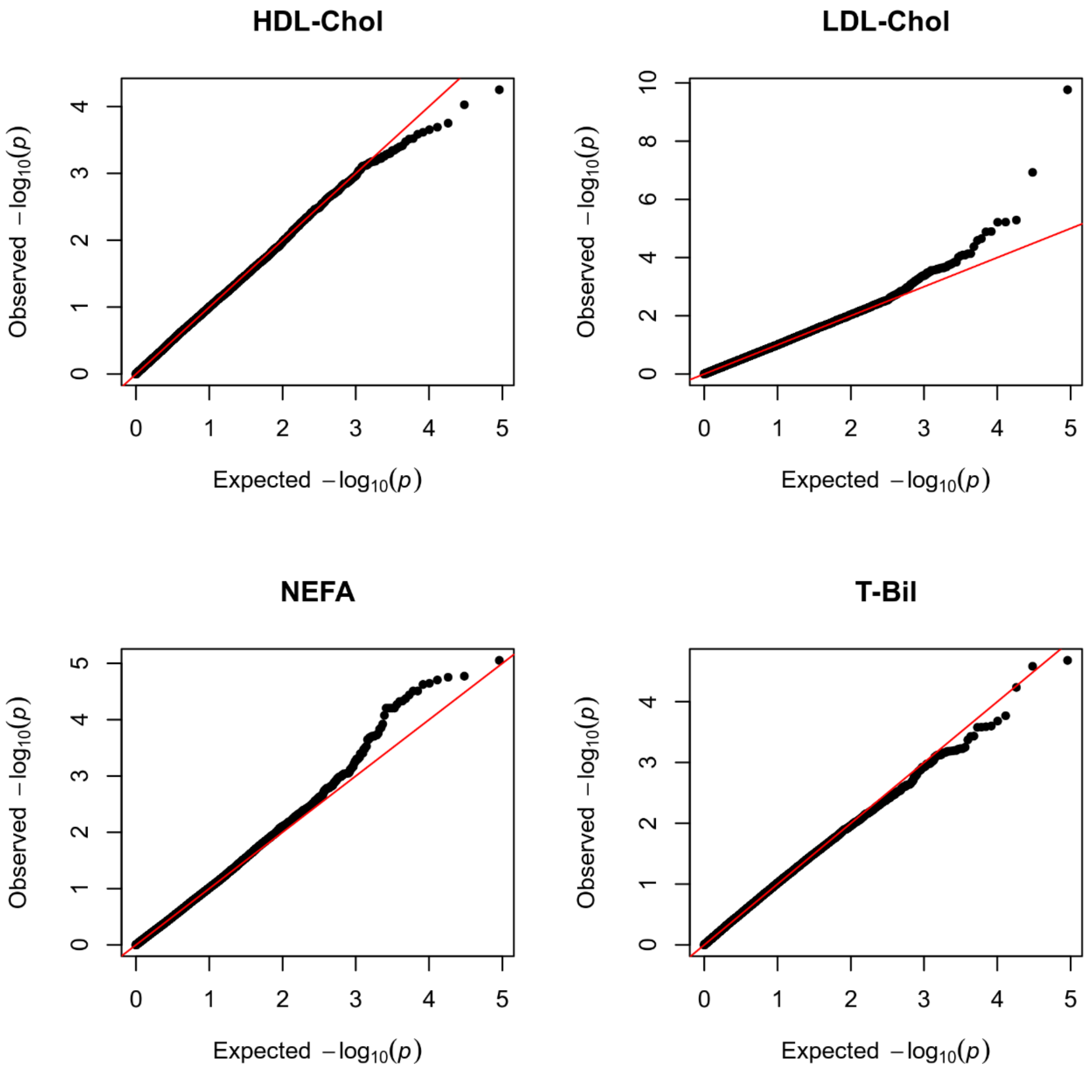

HDL-Chol
LDL-Chol
NEFA
T-Bil
Observed $-\log_{10}(p)$
Expected $-\log_{10}(p)$

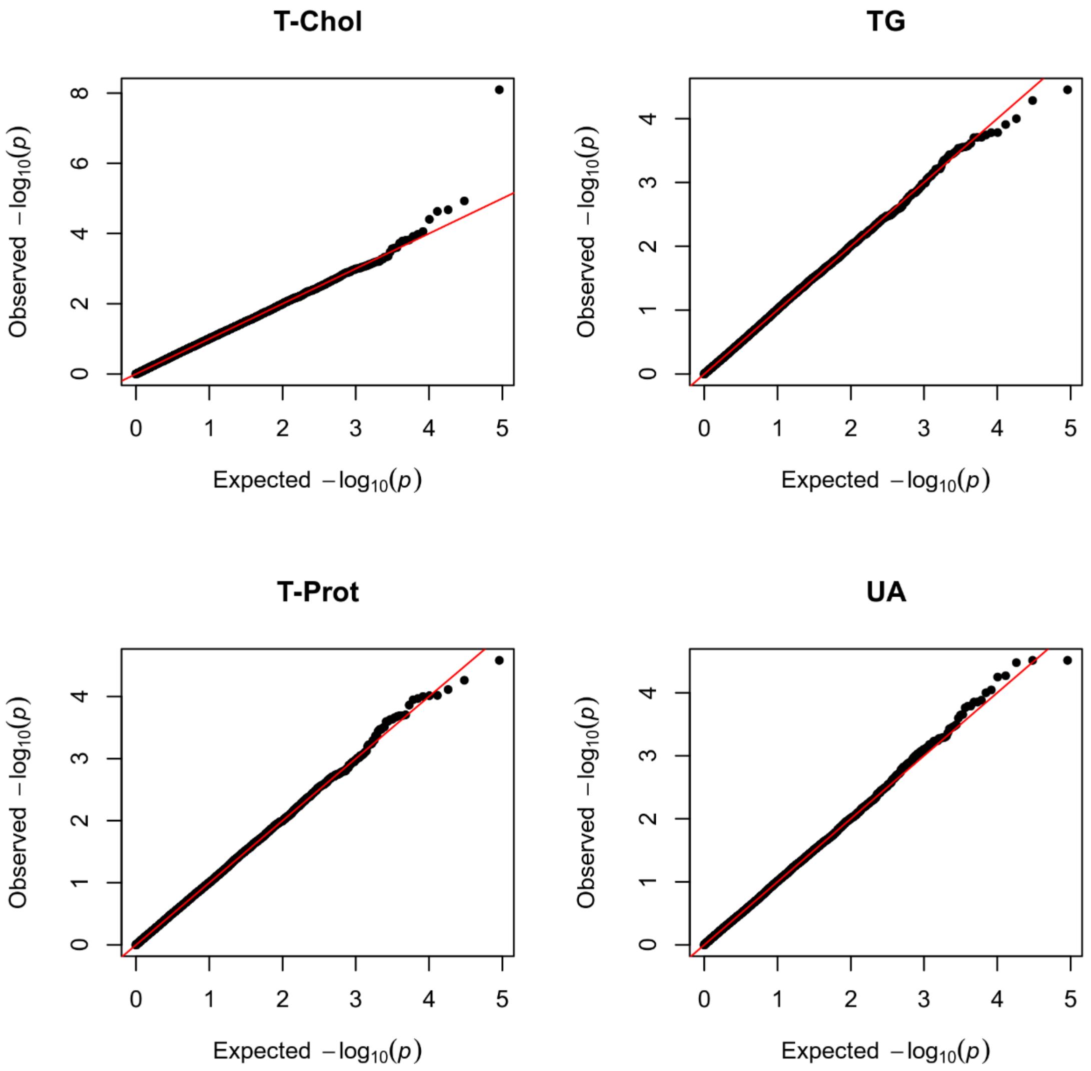

T-Chol
TG
T-Prot
UA
Observed $-\log_{10}(p)$
Expected $-\log_{10}(p)$
